# B-MASTER: scalable Bayesian multivariate regression for master predictor discovery in colorectal cancer microbiome-metabolite profiles

**DOI:** 10.1093/bioinformatics/btag500

**Published:** 2026-08-10

**Authors:** Priyam Das, Tanujit Dey, Christine B Peterson, Sounak Chakraborty

**Affiliations:** Department of Biostatistics, Virginia Commonwealth University, Richmond, VA 23219, United States; Center for Surgery and Public Health, Brigham and Women’s Hospital, Harvard Medical School, Boston, MA 02115, United States; Department of Statistics, Rice University, Houston, TX 77005, United States; Department of Statistics, University of Missouri, Columbia, MI 65211, United States

## Abstract

**Motivation:**

The gut microbiome shapes cancer therapy response through its influence on host metabolism. While prior studies examine pairwise associations between individual genera and metabolites, there is limited methodology for identifying microbial genera that systematically regulate the overall metabolome. Scalable statistical tools are needed to uncover such system-level “master predictors” in high-dimensional microbiome-metabolome data.

**Results:**

We introduce B-MASTER, a scalable Bayesian multivariate regression framework combining $\ell_{1}$ sparsity and $\ell_{2}$ group shrinkage to identify essential cross-metabolite regulators. A Gibbs sampler enables near-linear computational scaling, supporting models with millions of parameters. The method is supported by theoretical guarantees, including posterior contraction and selection consistency. Analysis of colorectal cancer microbiome-metabolome data reveals key microbial genera that govern global and cancer-associated metabolite patterns, highlighting system-level regulatory structure.

**Availability and implementation:**

The B-MASTER code, including demonstration scripts, is available at https://github.com/priyamdas2/B-MASTER. An archived snapshot of the code corresponding to this manuscript is available on Zenodo with DOI: 10.5281/zenodo.20484958.

## 1. Introduction

Advances in sequencing technologies and bioinformatic methods have revealed the critical role of the microbiome in human health and disease (Li 2015, Xu and Knight 2015). In colorectal cancer (CRC), gut microbial communities not only contribute to tumor initiation and progression but also influence therapeutic response (Garrett 2015). High-throughput sequencing now enables detailed analyses of microbial richness and abundance (Caporaso *et al.* 2011), and when combined with metabolomic profiling, offers a window into the microbial metabolites that shape immune regulation, inflammation, and cancer development (Belkaid and Hand 2014, Zhang *et al.* 2021).

**Figure 1:**
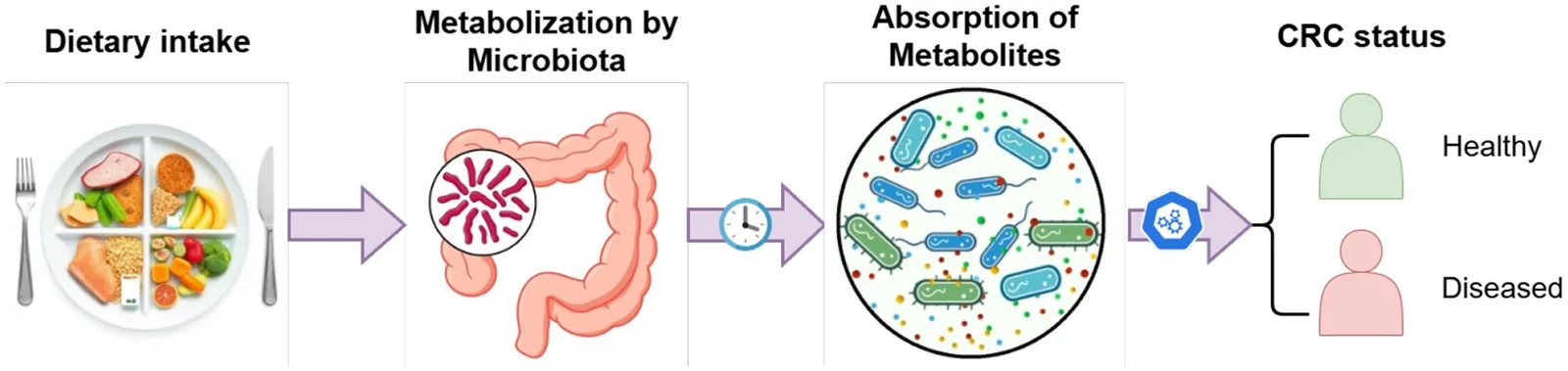
Conceptual illustration of how gut microbial activity influences metabolite production, which in turn may affect colorectal cancer progression.

The microbiome exerts its effects largely through metabolism. Metabolites such as short-chain fatty acids, amino acids, and fermentation products have been linked to both protective and pro-carcinogenic processes (Song *et al.* 2022, Zhang *et al.* 2023, Lu *et al.* 2025). Yet most existing studies focus on isolated pathways or pairwise associations, leaving open the broader question of which microbial genera systematically regulate the overall metabolite profile. This question is particularly important for metabolites that are differentially abundant in CRC, where identifying the microbial drivers may provide mechanistic insight and therapeutic opportunities (Yachida *et al.* 2019). A conceptual illustration of how microbial activity shapes metabolites and their downstream influence on colorectal cancer is provided in Fig. 1.

Our study aims to identify “master predictors”: microbial genera exerting coordinated influence across metabolite pathways. Statistically, this requires selecting regressors affecting multiple outcomes simultaneously while controlling false positives in high-dimensional settings. Variable selection has progressed from Ridge regression (Hoerl and Kennard 1970), LASSO (Tibshirani 1996), and Elastic-Net (Zou and Hastie, 2005) to structured extensions such as group and adaptive Lasso (Yuan and Lin 2006). Bayesian formulations, including the Bayesian Lasso and spike-and-slab approaches (Park and Casella 2008, Rockova and George 2018), further enhanced shrinkage-based inference, with scalable adaptations emerging for modern data (Qian *et al.* 2020). In multivariate regression, methods such as Bayesian group Lasso (Liquet *et al.* 2017), Spike-and-Slab Lasso (Deshpande *et al.* 2019), and reduced-rank regression (Chakraborty *et al.* 2020) improve sparse estimation across correlated outcomes. However, many remain computationally burdensome in ultra-high dimensions or do not explicitly target elimination of non-essential regressors. Frequentist approaches such as remMap (Peng *et al.* 2010) attempt master predictor identification but exhibit scalability limitations and instability under correlated predictors. These challenges motivate a scalable Bayesian framework tailored to high-dimensional microbiome-metabolome integration.

We address these challenges by proposing *Bayesian Multivariate regression Analysis for Selecting Targeted Essential Regressors* (B-MASTER), which reformulates the combined $l_{1}$ and $l_{2}$ penalization strategy within a fully Bayesian framework. Through a hierarchical prior specification, B-MASTER yields a Gibbs sampling algorithm whose computation time remains nearly invariant with sample size, permitting full posterior inference even in ultra-high-dimensional models. Compared to existing approaches, B-MASTER offers several advantages: (i) tuning parameters are automatically adjusted through hyperpriors, avoiding repeated cross-validation; (ii) posterior samples provide direct measures of uncertainty for regression coefficients, eliminating the need for computationally intensive bootstrapping; (iii) computation time is predictable and robust across datasets of similar dimension; and (iv) the algorithm scales efficiently to ultra-high-dimensional models, with demonstrated performance on problems involving up to four million parameters, while maintaining predictable linear computation time. Beyond computational efficiency, B-MASTER is supported by rigorous high-dimensional theory, establishing posterior contraction, selection consistency for master predictors, and robustness to correlated errors and mild model misspecification.

The remainder of the paper is organized as follows. Section 2 introduces the B-MASTER model, the associated Gibbs sampling scheme, and highlights its theoretical properties. Section 3 evaluates performance in simulation studies. Section 5 applies B-MASTER to identify master predictors regulating the metabolomics of CRC subjects. Section 6 concludes with a discussion of implications and future directions.

## 2. B-MASTER

### 2.1 Model

**Figure 2:**
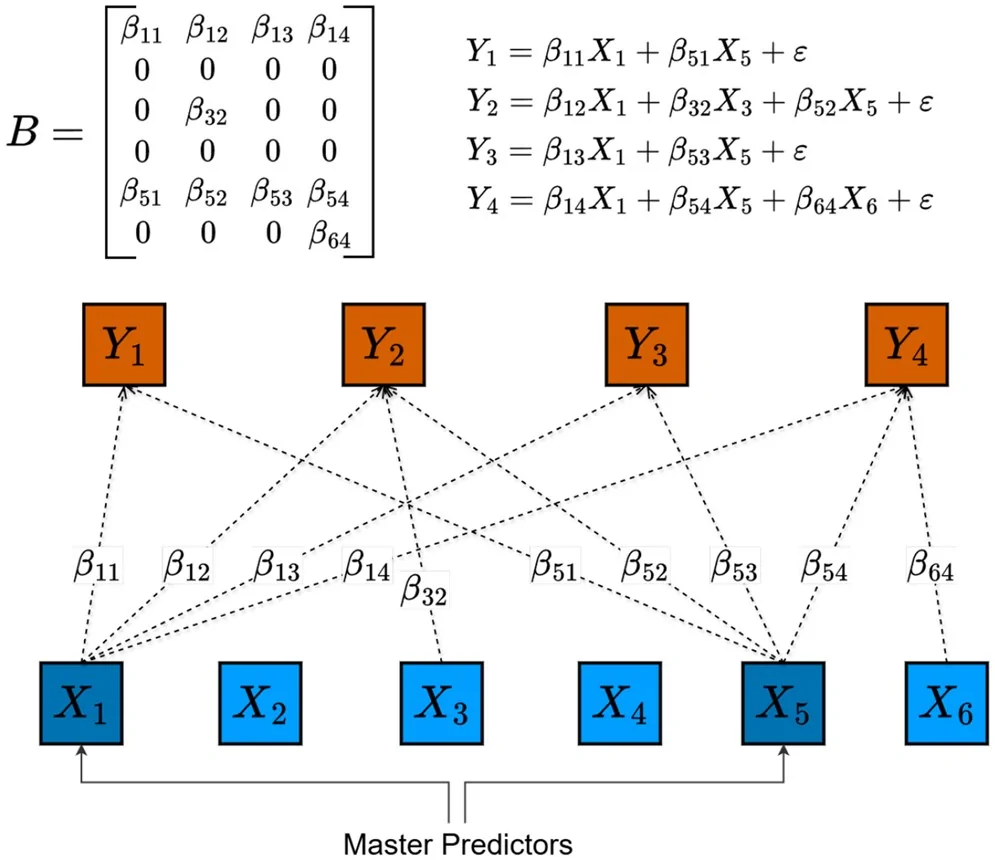
Conceptual diagram illustrating ‘master predictors’: predictors such as $X_{1}$ and $X_{5}$ exert influence on multiple response variables, highlighting their role as master predictors.

Suppose we have *N* observations with data $\left(x^{i},y^{i}\right)$ for i=1,…,N, where $x^{i}=(x_{1}^{i},\ldots ,x_{P}^{i})$ is a 1×P vector of predictors, and $y^{i}=(y_{1}^{i},\ldots ,y_{Q}^{i})$ is a 1×Q vector of responses. The multivariate regression model is given by


(1) $$y^{i}=x^{i}B+\epsilon^{i},\;i=1,\ldots ,N,$$

where $\epsilon^{i}\;\overset{i.i.d.}{∼}\;N_{Q}(0,\text{diag}(\sigma_{1}^{2},\ldots ,\sigma_{Q}^{2}))$. Here, $B=(\beta_{pq})$ denotes the P×Q matrix of regression coefficients. In our case study, we aim to identify the key components of the microbiome that exert the most significant influence on the overall microbiome-metabolite interaction. This hypothesis is grounded in recent studies, such as Liu *et al.* (2022), which explore the relationship between microbiome composition and metabolite production, demonstrating that specific microbiome species substantially affect the types and concentrations of metabolites within a host organism. Consequently, it is essential to impose sparsity on the B-matrix in a structured manner. It is also important to ensure that the proposed model not only achieves overall sparsity but also facilitates the identification of critical predictors, enabling a biologically interpretable understanding of the microbiome-metabolite relationship. A conceptual diagram of ‘master predictors’, which are subsequently identified using B-MASTER, is presented in Fig. 2.

### 2.2 Prior formulation and Gibbs sampling

Extensive research on variable selection has shown that, in high-dimensional settings, predictor-response relationships are often driven by a small subset of influential predictors (Qian *et al.* 2020). Penalized regression methods, such as LASSO (Tibshirani 1996), are designed to capture these sparse structures, even in the presence of multicollinearity. The $l_{1}$-penalty applied to the coefficients of B induces sparsity by shrinking many coefficients to zero. Additionally, to identify predictors with dominant effects across multiple response components, an $l_{2}$-norm can be applied to the coefficient vectors of each predictor (Peng *et al.* 2010). This hybrid penalization balances model parsimony and shrinkage for less significant predictors. Following Kyung *et al.* (2010), we adopt the conditional prior


(2) $$\pi (B|\sigma_{1}^{2},\ldots ,\sigma_{Q}^{2})\propto \;\text{exp}[-\lambda_{1}\sum_{p=1}^{P}\sum_{q=1}^{Q}\frac{\left|\beta_{pq}\right|}{2\sigma_{q}^{2}}-\lambda_{2}\sum_{p=1}^{P}{\left(\sum_{q=1}^{Q}\frac{\beta_{pq}^{2}}{2\sigma_{q}^{2}}\right)}^{1/2}].$$

The parameter $\lambda_{1}$ controls overall coefficient shrinkage, whereas $\lambda_{2}$ penalizes the $\ell_{2}$-norm of coefficients within each predictor across responses. Consequently, the $l_{1}$ component encourages sparsity among individual coefficients $\beta_{pq}$, while the group-wise $l_{2}$ component acts on the entire coefficient vector associated with predictor *p*. Predictors exhibiting weak effects across all responses are therefore shrunk toward zero, whereas predictors with broad influence across multiple responses are more likely to be retained. This structure is particularly appealing for identifying *master predictors*, namely predictors that influence many response variables simultaneously. Although the prior in (2) combines $l_{1}$- and $l_{2}$-type shrinkage, it differs fundamentally from the Elastic-Net penalty (Zou and Hastie, 2005). A detailed discussion of these differences is provided in Supplementary Section 1, available as supplementary data at *Bioinformatics* online.

To further characterize the prior structure, let $\beta^{\left(p\right)}=(\beta_{p1},\ldots ,\beta_{pQ})$ denote the coefficient vector corresponding to predictor *p*. Then (2) factorizes as


(3) $$\begin{array}{c} \pi (B|\sigma_{1}^{2},\ldots ,\sigma_{Q}^{2})=\prod_{p=1}^{P}\pi (\beta^{\left(p\right)}|\sigma_{1}^{2},\ldots ,\sigma_{Q}^{2}),\;\;\text{where},\\ \pi (\beta^{\left(p\right)}|\sigma_{1}^{2},\ldots ,\sigma_{Q}^{2})\propto \;\text{exp}[-\lambda_{1}\sum_{q=1}^{Q}\frac{\left|\beta_{pq}\right|}{2\sigma_{q}^{2}}-\lambda_{2}{\left\{\sum_{q=1}^{Q}\frac{\beta_{pq}^{2}}{2\sigma_{q}^{2}}\right\}}^{1/2}]. \end{array}$$

Although the prior in (2.2) directly encodes the desired sparsity structure, posterior computation under this formulation is not conjugate. Following Xu and Ghosh (2015), we therefore employ an equivalent Gaussian scale-mixture representation obtained through the introduction of latent variance components. This reparameterization preserves the original prior while enabling efficient Gibbs sampling through closed-form conditional updates. Specifically, we introduce latent parameters $T^{2}={\left(\tau_{1}^{2},\ldots ,\tau_{P}^{2}\right)}^{T}={\left(\tau_{pq}^{2}\right)}_{P\times Q}$ and $G^{2}={\left(\gamma_{p}^{2}\right)}_{P\times 1}$. Here, $\tau_{pq}^{2}$ is a coefficient-specific local variance parameter associated with $\beta_{pq}$, whereas $\gamma_{p}^{2}$ is a predictor-level variance parameter shared across all responses for predictor *p*. Small values of $\tau_{pq}^{2}$ encourage shrinkage of individual coefficients, while small values of $\gamma_{p}^{2}$ shrink the entire coefficient vector $\beta^{\left(p\right)}$, thereby reinforcing the master-predictor structure encoded by the prior. Under this hierarchical representation,


$$\beta^{\left(p\right)}|\tau_{p}^{2},\gamma_{p}^{2},\sigma_{1}^{2},\ldots ,\sigma_{Q}^{2}∼N_{Q}(0,\mathrm{diag}\left\{\sigma_{q}^{2}{\left(\frac{1}{\tau_{pq}^{2}}+\frac{1}{\gamma_{p}^{2}}\right)}^{-1}\right\}),$$

The mixing distribution of $\left(\tau_{p}^{2},\gamma_{p}^{2}\right)$, the proof that integrating out the latent variables recovers the prior in (2.2), and the corresponding propriety results are provided in Supplementary Sections 1 and 2, available as supplementary data at *Bioinformatics* online (Lemma S2.1, available as supplementary data at *Bioinformatics* online). We place a non-informative prior on $\sigma_{q}^{2}$, namely $\pi (\sigma_{q}^{2})\propto 1/\sigma_{q}^{2}$ for q=1,…,Q, and Gamma priors on the squared penalty parameters $\lambda_{1}^{2}$ and $\lambda_{2}^{2}$. The latent variables are introduced solely as an equivalent hierarchical representation of the prior and are not parameters of primary scientific interest; their role is to facilitate efficient posterior computation within the Gibbs sampling framework.

Combining the likelihood from model (1) with the aforementioned prior specification yields the full posterior distribution, from which a Gibbs sampling scheme can be derived. While the structure of the posterior and the detailed conditional distributions are deferred to Supplementary Section 1, available as supplementary data at *Bioinformatics* online, we provide below a skeletal outline of the proposed Gibbs sampler:

- Sample $\beta_{q}={\left(\beta_{1q},\ldots ,\beta_{Pq}\right)}^{T}$ for q=1,…,Q, from a multivariate normal distribution.
- Sample $\tau_{pq}^{2}$ for p=1,…,P;q=1,…,Q, such that $\tau_{pq}^{2}=\frac{1}{\delta_{pq}^{\left(1\right)}}$, where $\delta_{pq}^{\left(1\right)}$ is sampled from inverse Gaussian distribution.
- Sample $\gamma_{p}^{2}$ for p=1,…,P, such that $\gamma_{p}^{2}=\frac{1}{\delta_{p}^{\left(2\right)}}$, where $\delta_{p}^{\left(2\right)}$ is sampled from inverse Gaussian distribution.
- Sample $\sigma_{q}^{2}$ for q=1,…,Q, from inverse Gamma distribution.
- Sample $\lambda_{1}^{2}$ and $\lambda_{2}^{2}$ from their Gamma full conditional distributions.

In B-MASTER, variable selection is performed as a post-processing step based on posterior summaries of the regression coefficients. Specifically, genus–metabolite edges are selected using credible-interval-based posterior evidence, rather than by thresholding posterior means directly; details of the selection rule and associated Bayesian posterior *P* value are provided in Supplementary Section 3.1, available as supplementary data at *Bioinformatics* online.

### 2.3 Computational scalability

A major motivation for B-MASTER is the development of a scalable Bayesian framework for identifying master predictors in high-dimensional microbiome–metabolome studies. While predictive performance and variable-selection accuracy are evaluated in the simulation studies of Section 3, an equally important consideration is computational feasibility as the dimensions of the problem increase. To provide context for the scalability results presented later in Section 3.2, we briefly contrast the computational structure of B-MASTER with that of remMap (Peng *et al.* 2010), a widely used frequentist approach for master predictor identification.

**Figure 3:**
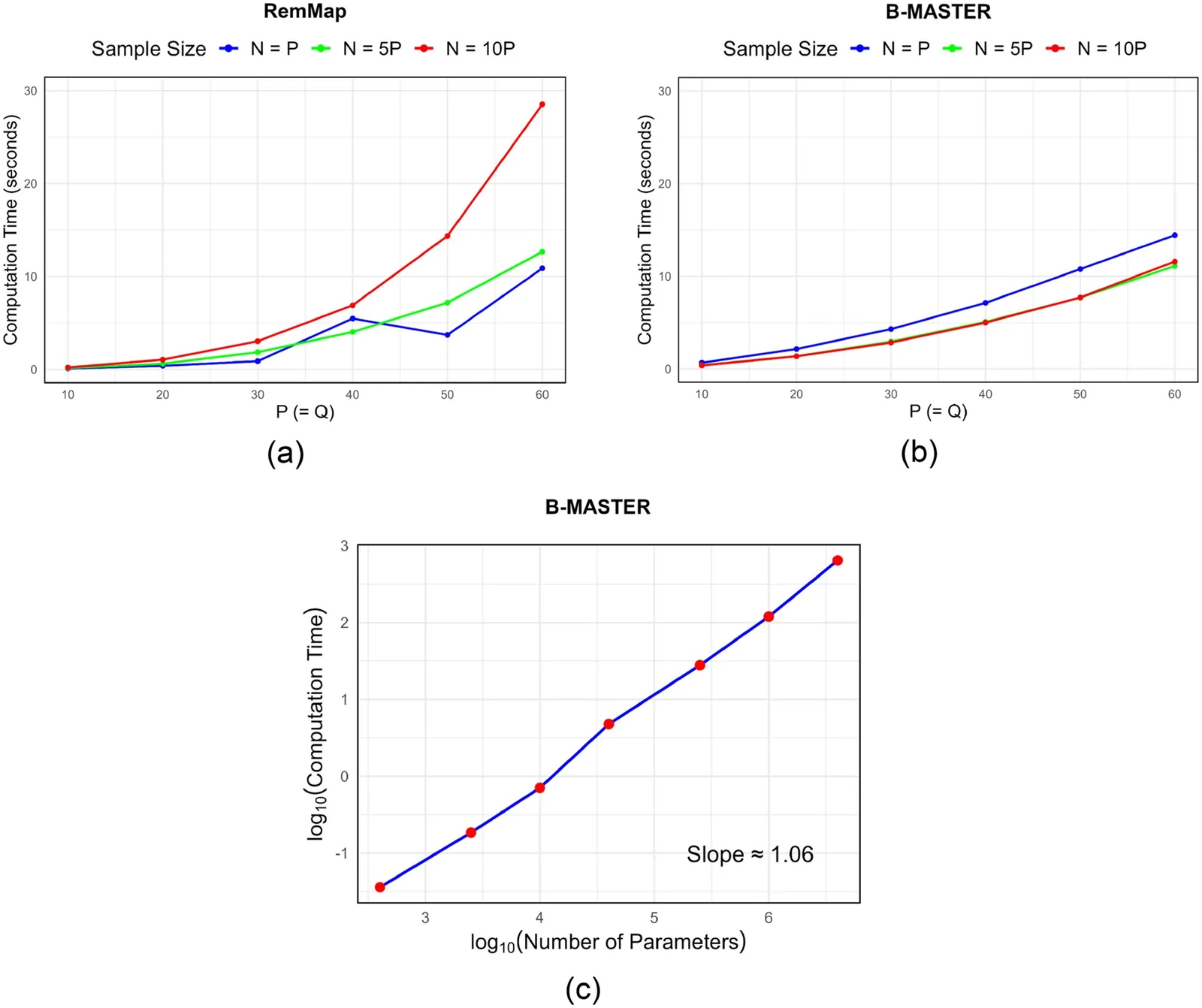
(a) Computation times for remMap with P=Q=10,20,30,40,50,60 and N=P,5P,10P. (b) Computation times for B-MASTER under the same settings. While remMap runtime increases rapidly with *N*, B-MASTER shows near invariance to sample size for fixed (P,Q). (c) Log–log plot of the number of parameters versus computation time for B-MASTER. The slope ≈1.06 indicates near-linear scaling.

RemMap optimizes B via the “active-shooting” algorithm (Peng *et al.* 2009) with computational complexity O(NPQ). As sample size increases, this leads to substantial computational burden. In contrast, the proposed Gibbs sampler performs most updates through parameter-wise conditional distributions, with only limited dataset-dependent operations that are efficiently handled by MATLAB’s multi-threading (MathWorks 2024). Consequently, for fixed *P* and *Q*, computation time remains largely invariant as *N* grows. Figure 3a and b (see Section 3.2) illustrate that remMap runtime scales strongly and irregularly with *N*, whereas B-MASTER exhibits stable and predictable scaling.

**Figure 4:**
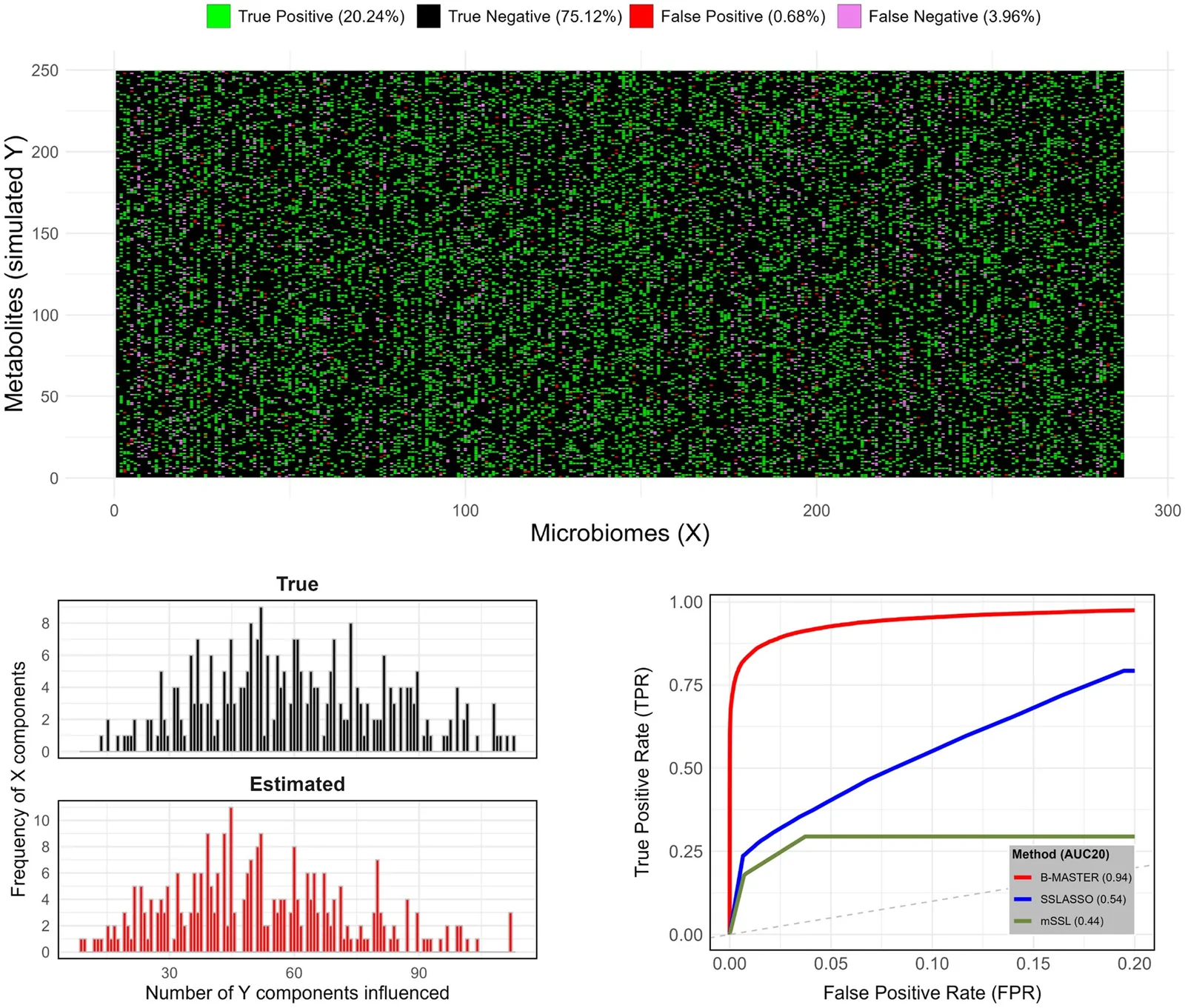
*Top*: Signal detection by B-MASTER, with TP, TN, FP, FN categories. *Bottom-left*: Distribution of number of *Y* influenced per *X*, true vs. B-MASTER estimates. *Bottom-right*: AUC curves up to 20% FPR for B-MASTER, SSLASSO, and mSSL.

Moreover, implementation of remMap typically involves an additional tuning-parameter selection step, often based on cross-validation, while uncertainty assessment may require bootstrap-based procedures, both of which can increase overall computational cost. In contrast, the Bayesian framework underlying B-MASTER provides coefficient estimation and uncertainty quantification within a single model fit. Figure 3a further illustrates that remMap runtime can vary noticeably across datasets even when (P,Q) are held fixed. For example, when P=Q=40, the runtime for N=40 exceeded that for N=200, with similar behavior observed for P=Q=50. By comparison, Fig. 3b shows that B-MASTER exhibits more stable scaling behavior, with only modest runtime variation attributable to implementation-level factors such as increased multi-threading efficiency in higher-dimensional settings (MathWorks 2024). The near-linear scaling observed in Fig. 3c is an empirical property of the proposed implementation. In practice, most updates in the proposed Gibbs sampler involve closed-form conditional distributions together with matrix operations that can be efficiently handled through MATLAB’s optimized multi-threaded linear algebra routines (MathWorks 2024). As a result, the observed wall-clock runtime may benefit substantially from parallel execution of low-level matrix computations, with the degree of improvement depending on the extent to which individual operations can be effectively multi-threaded. These implementation characteristics contribute to the stable computational performance observed across the high-dimensional settings considered in this study.

## 3. Simulation study

In this section, we consider two complementary simulation studies. The first study is a data-driven simulation designed to closely mimic the structural characteristics of the motivating microbiome–metabolome dataset, including the observed predictor dependence and sparsity patterns. The second study is a fully synthetic high-dimensional simulation in which sparse coefficient structures are generated independently under both independent and correlated predictor settings. Together, these studies allow us to assess B-MASTER under realistic data conditions as well as under controlled settings where the true generating mechanism is known.

### 3.1 Data-driven simulation based on the CRC dataset

We base the first simulation study on the Curated Gut Microbiome-Metabolome Data Resource (Muller *et al.* 2022), matching the case study dimensions after preprocessing (P=287, Q=249, N=220). Using B-MASTER, we estimate a coefficient matrix $B^{\prime }$ and treat it as the ground truth. Synthetic responses are generated as $Y_{q}^{i}=X^{i}B_{q}^{\prime }+\epsilon_{iq}$ with $\epsilon_{iq}∼N(0,1)$, using the observed *X* to preserve its empirical covariance structure (see Fig. S9). Ten replicate datasets are generated.

B-MASTER is implemented in MATLAB and run for 1,000 iterations with 100 burn-in (see Supplementary 1.5, available as supplementary data at *Bioinformatics* online for parameter settings). Competing methods include remMap (Peng *et al.* 2010) and univariate/multivariate Spike-and-Slab LASSO (SSLASSO, Rockova and George 2018, mSSL, Deshpande *et al.* 2019) via their R packages. Since the default cross-validation procedures did not terminate within 48 hours for remMap and mSSL under the considered high-dimensional settings, computationally feasible package-supported alternatives were adopted. For remMap, tuning parameters were selected by minimizing the Bayesian Information Criterion (BIC) over an 11×11 grid with $\lambda_{1}\in [1,1000]$ evaluated on a logarithmic scale and $\lambda_{2}\in [0,1000]$ evaluated on a uniform scale, consistent with the parameter-grid construction illustrated in the package demonstration code. For SSLASSO and mSSL, we used the package’s default approximate fitting and tuning strategy, as adopted in the corresponding package demonstrations. Additional details on implementation, computational environment, tuning procedures, evaluation metrics (e.g., MCC and AUC20), and posterior edge selection are provided in Supplementary Section 3, available as supplementary data at *Bioinformatics* online.

Convergence diagnostics and sensitivity analyses are reported in Supplementary Section 4, available as supplementary data at *Bioinformatics* online, including Geweke statistics and MCSE/SD% ratios (Table S5) with supporting trace plots (Fig. S3), robustness to prior hyperparameter choices (Table S6), and sensitivity to the total number of MCMC iterations (Figs. S4–S6 and Table S7, available as supplementary data at *Bioinformatics* online). In the iteration-length analysis, B-MASTER produced nearly identical TPR, FPR, MCC, AUC, AUC20, and sparsity estimates across 500, 1000, and 2000 total iterations, each with 100 burn-in iterations, supporting the stability of the reported posterior selection results. Overall, B-MASTER achieves accurate sparsity recovery with strong signal detection, remains slightly conservative in the number of outcomes influenced per predictor, and consistently outperforms SSLASSO and mSSL, particularly in the low-FPR regime (see Fig. 4 and Table 1).

### 3.2 Higher-dimensional evaluation

To assess B-MASTER in higher-dimensional regimes, we conducted an additional simulation study under settings with P=Q=N ranging from 20 to 2000, with both independent (ρ=0) and correlated (ρ=0.5) predictor structures. The data-generating mechanism was designed to mimic the master-predictor setting, where 20% of predictors influenced all responses, an additional 20% influenced a randomly selected subset of responses, and the remaining 60% had no effect. Full simulation details are provided in Supplementary Section 3.2, available as supplementary data at *Bioinformatics* online.

The results are summarized in Table 2. Across all considered dimensions and correlation structures, B-MASTER achieved consistently high TPR values, near-zero FPR values, and excellent overall discrimination with AUC values. The corresponding MCC values remained high, indicating accurate recovery of the underlying sparse coefficient structure. Estimated sparsity closely matched the true sparsity across all settings, with only a slight tendency toward conservative selection. Although performance was marginally stronger under independent predictors (ρ=0), the degradation under correlated predictors (ρ=0.5) was modest, even at the largest dimensions. Notably, B-MASTER maintained strong variable-selection performance when the coefficient matrix contained up to four million parameters (P=Q=2000), demonstrating its ability to accurately identify master predictors in large-scale multivariate settings. Complete results for all scenarios are reported in Table S3. We further compared B-MASTER with SSLASSO and remMap-bic for the lower-dimensional settings P=Q=N∈{20,50,100,200}; as shown in Table S4, B-MASTER achieved comparable or higher discriminative performance while maintaining substantially lower FPR and more accurate sparsity recovery. The competing methods often attained high TPR, but at the cost of inflated FPR and less precise support recovery.

**Table 1:** Comparative study of B-MASTER, SSLASSO, mSSL, and remMap in the first simulation study based on true positive rate (TPR), false positive rate (FPR), Matthews Correlation Coefficient (MCC), AUC, and AUC20.

Methods	TPR	FPR	MCC	AUC	AUC20	Sparsity (True = 0.76).
B-MASTER	0.84 (0.0012)	0.01 (0.0003)	0.87 (0.0001)	0.98 (0.0004)	0.94 (0.0014)	0.79 (0.0003)
SSLASSO	0.23 (0.0008)	0.01 (0.0004)	0.41 (0.0013)	0.89 (0.0004)	0.54 (0.0007)	0.94 (0.0002)
mSSL-dcpe	0.14 (0.0018)	0.00 (0.0001)	0.31 (0.0024)	0.76 (0.0006)	0.44 (0.0013)	0.96 (0.0005)
remMap-bic	0.00 (0.0001)	0.00 (0.0000)	0.02 (0.0008)	0.58 (0.0015)	0.23 (0.0009)	1.00 (0.0000)

Results are averages over 10 replicates; standard errors are reported in parentheses.

**Table 2:** Performance evaluation of B-MASTER in the higher-dimensional simulation study based on true positive rate (TPR), false positive rate (FPR), Matthews correlation coefficient (MCC), AUC, AUC20, and sparsity recovery.

ρ	*P, Q, N*	TPR	FPR	MCC	AUC	AUC20	Sparsity (True sparsity).
ρ=0	20	0.974	0.000	0.982	0.999	0.999	0.715 (0.708)
	50	0.991	0.000	0.993	1.000	1.000	0.708 (0.705)
	100	0.978	0.012	0.964	0.997	0.986	0.699 (0.700)
	200	0.972	0.026	0.937	0.995	0.980	0.692 (0.702)
	500	0.996	0.029	0.951	0.998	0.990	0.681 (0.699)
	1000	0.998	0.006	0.989	0.999	0.999	0.697 (0.700)
	2000	0.998	0.000	0.998	1.000	1.000	0.702 (0.701)
ρ=0.5	20	0.966	0.000	0.976	1.000	1.000	0.718 (0.708)
	50	0.999	0.002	0.996	1.000	1.000	0.704 (0.705)
	100	0.982	0.025	0.947	0.994	0.970	0.689 (0.700)
	200	0.963	0.033	0.921	0.992	0.969	0.690 (0.702)
	500	0.990	0.021	0.959	0.998	0.991	0.688 (0.699)
	1000	0.984	0.001	0.988	1.000	1.000	0.705 (0.700)
	2000	0.947	0.000	0.963	1.000	0.999	0.717 (0.701)

Results are reported for P=Q=N∈{20,50,100,200,500,1000,2000} under scenarios ρ=0 and ρ=0.5. Estimated sparsity values are reported with the corresponding true sparsity shown in parentheses.

## 4. Theoretical properties

The mathematical framework underlying B-MASTER is presented in detail in Supplementary Section 2, available as supplementary data at *Bioinformatics* online, where all assumptions, lemmas, and proofs are elaborated. Here we provide a concise summary of the key conditions and main results most relevant to the implemented B-MASTER model. The theoretical results are established for the same continuous shrinkage prior on B introduced in Section 2, namely the combined element-wise $\ell_{1}$ and predictor-level $\ell_{2}$ shrinkage prior. The latent variables $T^{2}$ and $G^{2}$ introduced in Section 2 provide an equivalent Gaussian scale-mixture representation of this prior for Gibbs sampling; after marginalizing over these latent variables, the induced prior on B is the prior used in the theoretical analysis. Thus, separate assumptions on $T^{2}$ and $G^{2}$ are not required in (A1)–(A5) noted below, because they are auxiliary variables used to represent the same marginal prior rather than additional scientific components of the model.

The mathematical framework underlying B-MASTER is presented in detail in Supplementary Section 2, available as supplementary data at *Bioinformatics* online, where all assumptions, lemmas, and proofs are elaborated. Here we provide a concise summary of the key conditions and main results most relevant to B-MASTER. For convenience, only in this subsection we adopt the notation $Y=XB_{0}+E$ with $X\in R^{N\times P}$, $Y\in R^{N\times Q}$, coefficient matrix $B_{0}$, and sparsity level $s=\#\{(p,q):\beta_{0,pq}\neq 0\}$. Let $\delta_{N}=\sqrt{s\;\text{log}(PQ)/N}$. The theoretical results rely on standard high-dimensional regularity conditions: (A1) restricted eigenvalue design; (A2) sparsity scaling s log(PQ)=o(N); (A3) bounded diagonal error variances; (A4) bounded eigenvalues with sparse precision under correlated errors; and (A5) hierarchical shrinkage priors, including (i) combined $\ell_{1}$-$\ell_{2}$ continuous shrinkage with Gamma hyperpriors, (ii) Jeffreys or heavy-tailed variance priors, and (iii) sparse precision priors for correlated-error extensions (see Supplementary 2, available as supplementary data at *Bioinformatics* online for precise formulations). The B-MASTER implementation is justified under assumptions (A1)–(A3) and (A5)(i)–(ii), and therefore the implemented method is directly covered by Theorems 1, 2, and 4. Assumptions (A4) and (A5)(iii), by contrast, are not part of the current implementation; they are introduced only for the correlated-error theoretical extension and are used in Theorem 3.

Theorem 1 (Posterior contraction and consistency (diagonal errors)).

Under (A1)–(A3) and (A5)(i)–(ii), for any fixed M>0, as N→∞,

$$\Pi \left(\|B-B_{0}{\|}_{F}>M\delta_{N}|X,Y\right)\;\to \;0\;\text{in}\;P_{B_{0},\Sigma_{0}}-\text{probability}.$$

Theorem 2 (Posterior contraction under misspecification).

Under (A1), (A2), and (A5)(i),(ii), the posterior contracts at rate $\sqrt{s\;\text{log}(PQ)/N}$ even under model misspecification. Specifically, when the true errors are sub-Gaussian with covariance $\Sigma_{0}$ (not necessarily diagonal), the posterior concentrates around the KL-optimal projection $\left(B^{⋆},\Sigma^{⋆}\right)$ of the working diagonal Gaussian model. In the linear-mean setting, $B^{⋆}=B_{0}$, implying consistent recovery of the true coefficient matrix despite covariance misspecification.

Theorem 3 (Posterior contraction under correlated errors).

Under assumptions (A1), (A2), (A4) and (A5)(i),(iii), the posterior contracts jointly for (B,Ω) under correlated errors. Specifically, B contracts at rate $\delta_{N}=\sqrt{s\;\text{log}(PQ)/N}$ and the precision matrix Ω at rate $\varepsilon_{\Omega }≍\sqrt{s_{\Omega }\;\text{log}\;Q/N}$ in Frobenius norm. Thus, even with correlated residuals, estimation of B achieves the same rate as in the diagonal-error case (up to constants), while consistently recovering the sparse precision structure.

Theorem 4 (Sure screening and selection consistency).

Under (A1)–(A3) and (A5)(i)–(ii), let $\tau_{N}:=C\sqrt{\;\text{log}(PQ)/N}$ and $\alpha_{N}:=e^{-cN\tau_{N}^{2}}$ for fixed C,c>0. Then:

- (Sure screening) $S_{\text{row}}\subseteq \hat{S}$ with probability →1.
- (Exact selection under beta-min) If $\beta_{\min}:=\min_{p\in S_{\text{row}}}\|\beta_{0,p\cdot }{\|}_{2}\geq K\sqrt{\;\text{log}(PQ)/N}$ for sufficiently large K, then $\hat{S}=S_{\text{row}}$ with probability →1.

## 5. Master predictors in CRC microbiome-metabolome analysis

**Figure 5:**
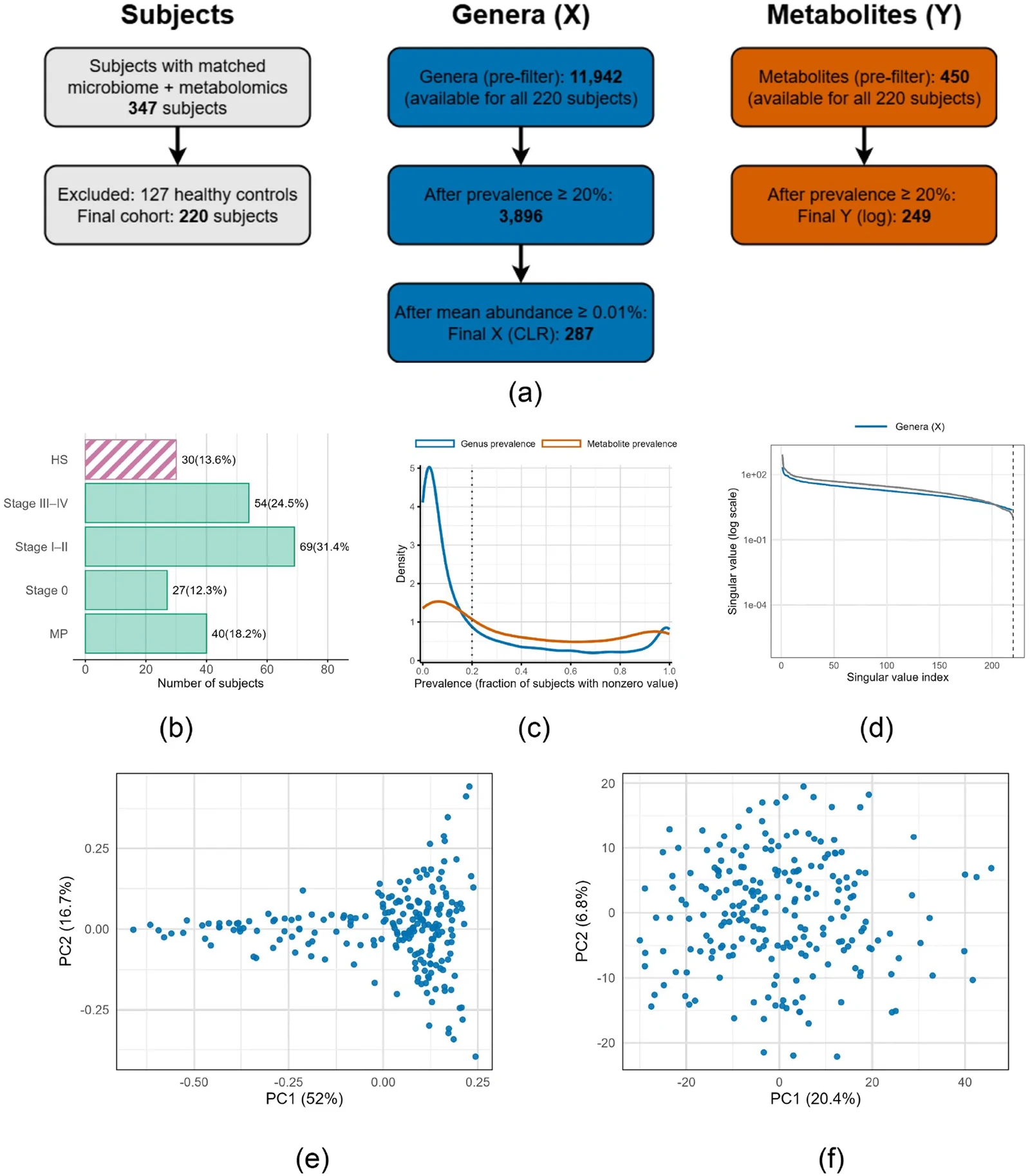
Overview of the CRC microbiome–metabolome dataset. (a) Cohort and filtering workflow. (b) Stage distribution. (c) Density of genus and metabolite prevalence. (d) Scree plot of singular values of *X* (CLR scale). (e and f) First two PCs of genera before/after CLR transformation.

We analyze matched microbiome and metabolome data from 347 subjects recruited at the National Cancer Center Hospital (Tokyo) (Muller *et al.* 2022). After filtering, the final cohort includes 220 CRC subjects, 287 microbial genera, and 249 metabolites (Fig. 5). The cohort spans stages 0–IV, precancerous multiple polypoid adenomas, and post-surgical patients. Microbial abundances were provided on a relative-abundance scale and were filtered to retain genera present in at least 20% of samples with mean abundance exceeding 0.01%. To address compositionality, we applied a centered log-ratio (CLR) transformation; before taking logarithms, exact zero entries were replaced internally by a pseudocount equal to one-half of the smallest positive relative abundance, following the default implementation of the CLR transformation in the microbiome R package and common practice in compositional microbiome analyses. We adopted the CLR transformation as it preserves the original dimension of the input data, making the regression coefficients more interpretable, while controlling for differences in sequencing depth and handling skewness of the input features (Aitchison 1982, Swift *et al.* 2023). Metabolites present in fewer than 20% of samples were removed; remaining zero values were replaced by one-half of the smallest observed positive metabolite value before log transformation. Exploratory analyses reveal typical high-dimensional challenges, including sparsity, non-Gaussianity, compositional dependence in microbiome measurements, and substantial cross-domain correlation. Figs. S7–S9, available as supplementary data at *Bioinformatics* online, further highlight covariate distributions, non-Gaussian metabolite residuals, and cross-domain correlations. To identify key microbiome components influencing metabolites, B-MASTER is fitted with hyper-parameters consistent with the simulation study. Inference is pursued based on 2000 posterior samples, with the first 100 discarded as burn-in.

To provide an interpretable measure of how influence is distributed across metabolites, we introduce a *Fractional Influence Score (FIS)* for each microbial genus identified by B-MASTER. For any metabolite outcome $Y_{q}$, suppose it is influenced by $h_{q}$ genera (as determined by the B-MASTER selection step). Instead of attributing a full unit of influence to every selected genus, we allocate equal shares: each contributing genus receives a score of $1/h_{q}$. Summing these fractional contributions across all metabolites yields the overall score for a genus,


$$\text{FIS}(g)=\sum_{q=1}^{Q}\frac{1\{g\in I_{q}\}}{h_{q}},$$

where $I_{q}$ is the set of influencing genera for metabolite *q*. Because $h_{q}$ appears in the denominator, this score naturally distinguishes genera that uniquely regulate a metabolite from those that appear only as part of larger sets of co-predictors. Posterior uncertainty for selected genus–metabolite associations is summarized through credible-interval-based selection and Bayesian posterior *P-*value, with additional FIS sensitivity summaries reported in Supplementary Section 5.2, available as supplementary data at *Bioinformatics* online. Specifically, the Bayesian posterior *P* value is computed as $2\min({\hat{p}}^{+},{\hat{p}}^{-})$, where ${\hat{p}}^{+}$ and ${\hat{p}}^{-}$ denote the posterior proportions of samples greater than zero and less than or equal to zero, respectively; details are provided in Supplementary Section 3.1, available as supplementary data at *Bioinformatics* online.

**Figure 6:**
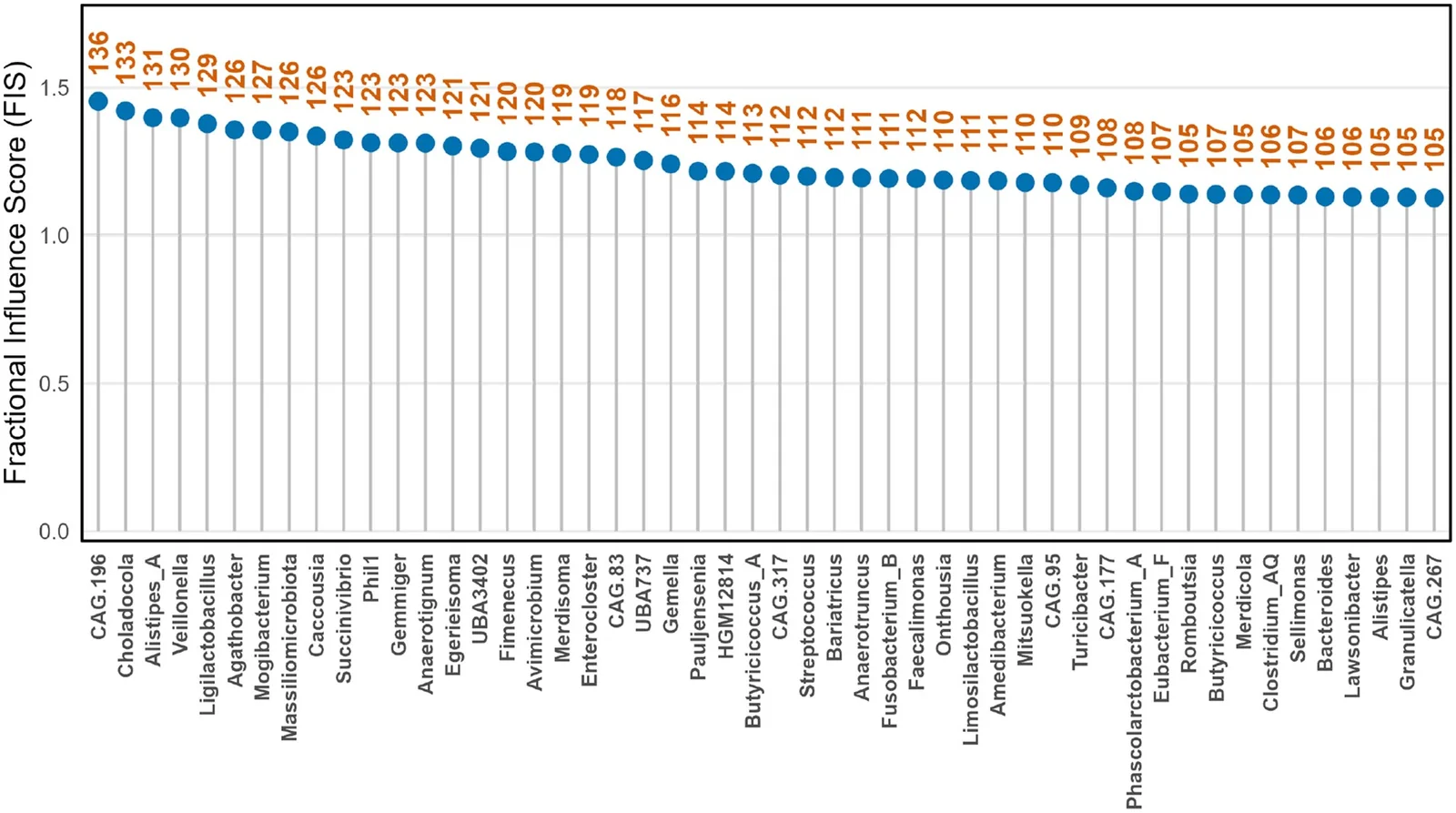
Functional Influence Scores (FIS) for the top 50 genera identified by B-MASTER. Bar height reflects each genus’s FIS; the numbers above the bars indicate, for that genus, the number of metabolites it influences.

**Figure 7:**
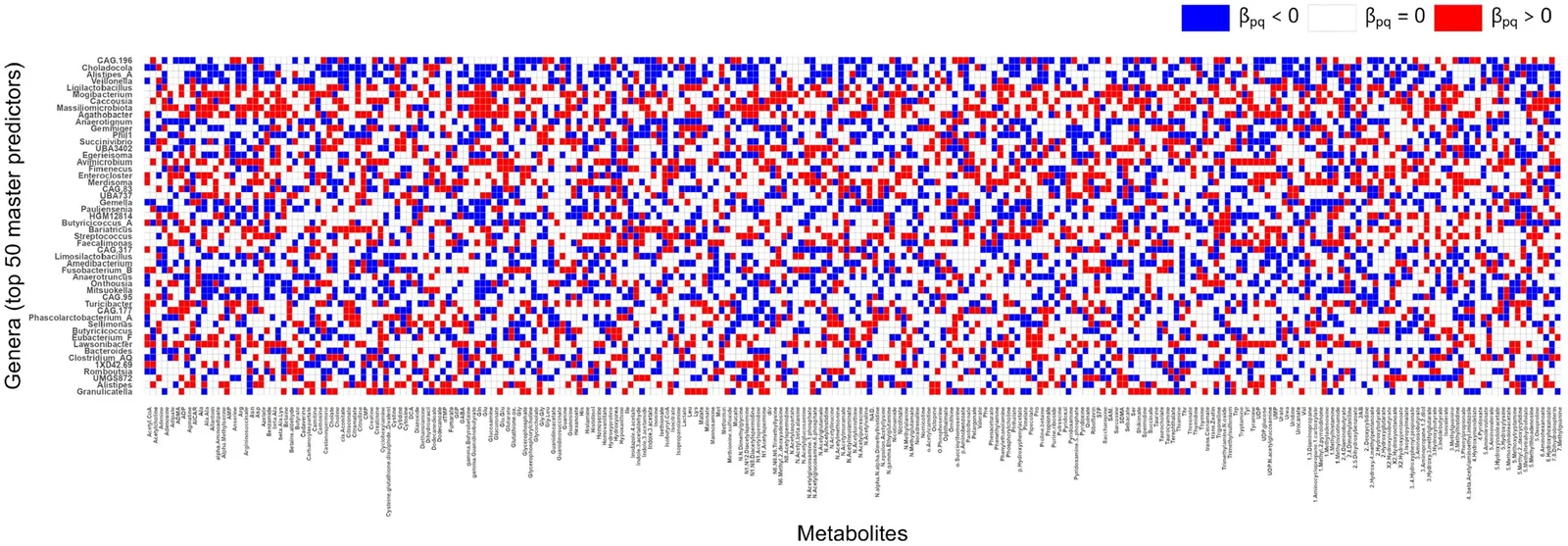
Heatmap illustrating the direction of the impact for each of the identified top 50 master predictors on each metabolite.

Using B-MASTER, we identify the top 50 microbiome genera influencing the largest number of metabolites based on FIS scores (Fig. 6), with the number of metabolites regulated by each genus shown above the bars. The top 50 genera were selected as a visualization-focused subset and together account for approximately 25% of the cumulative FIS across all 287 genera, while the complete ranked FIS distribution is provided in Supplementary Section 5.2, available as supplementary data at *Bioinformatics* online, which further reports the full FIS distribution for all 287 genera, together with rankings under 90% (default), 95%, and 99% posterior selection criteria and median Bayesian posterior *P* value across selected metabolite associations (see Tables S8–S13). These summaries provide additional uncertainty assessment for the FIS rankings and show that the top-50 threshold is used for visualization and interpretability rather than as a strict biological cutoff. Several master predictors align with prior findings. Davar and Zarour (2022) reported *CAG.196* and *Choladocola* (ranks 1–2) as key contributors to microbiome-host metabolism, particularly involving bile acids and short-chain fatty acids (SCFAs). *Alistipes* (rank 3) modulates bile acids and SCFAs (Parker *et al.* 2020), while *Veillonella* (rank 4) and *Ligilactobacillus* (rank 5) participate in lactate, propionate, and bile salt metabolism (Zhang and Huang 2023). Song *et al.* (2022) linked *Mogibacterium* (rank 7) to increased SCFAs and fat with negative correlations to amino acids. *Agathobacter*, *Gemmiger*, and *Anaerotignum* (ranks 6, 12, 13) contribute to fiber degradation and SCFA production, and *Massiliomicrobiota* (rank 8) has been associated with amino acid and energy metabolism (Lu *et al.* 2025). In contrast, genera such as *Caccousia*, *Succinivibrio*, and *Phil1* (ranks 9–11) remain sparsely characterized, highlighting potentially novel regulators. Figure 7 presents a heatmap of the top 50 predictors, illustrating their metabolite-specific effects and directionality.

**Figure 8:**
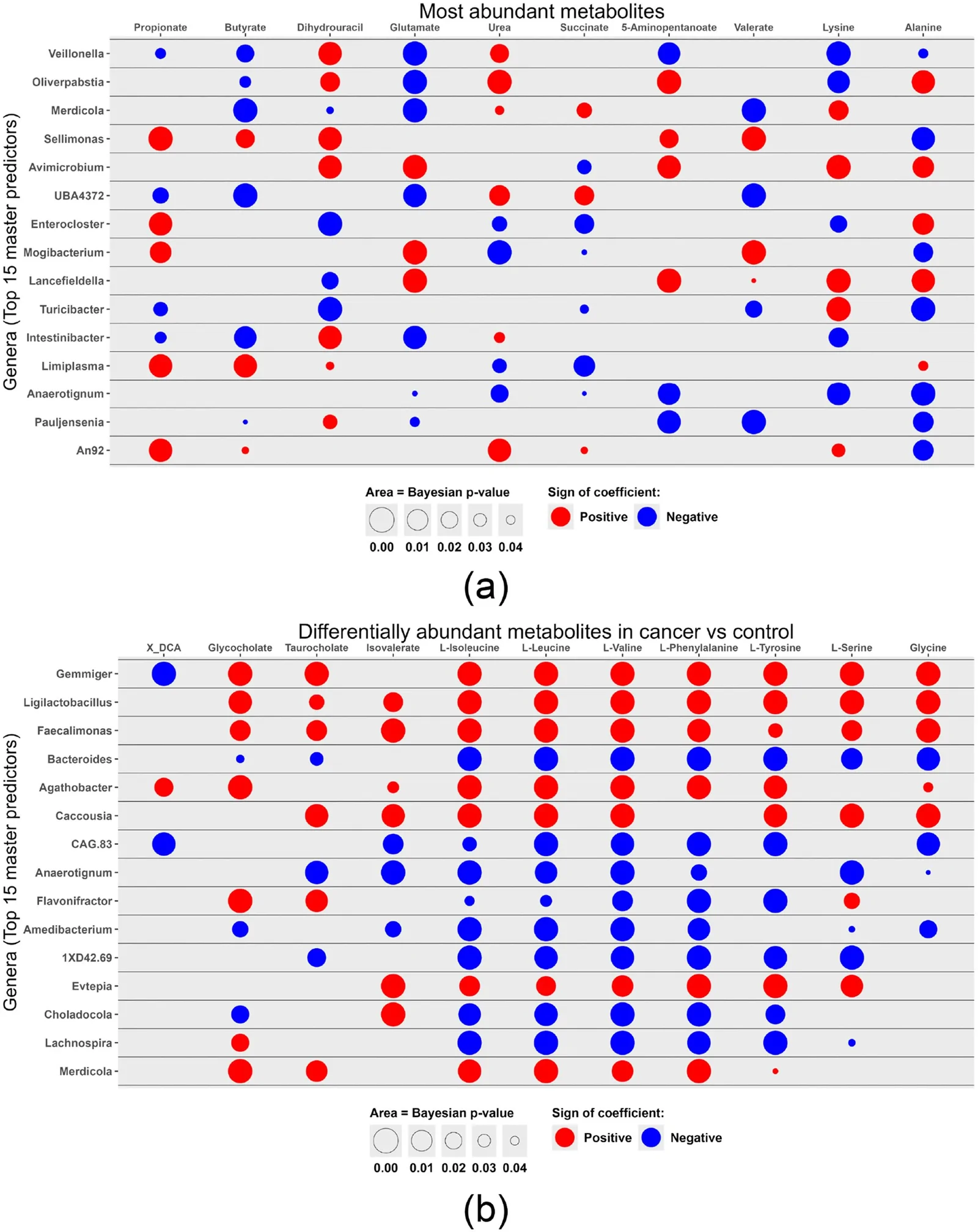
The plot demonstrates the direction and statistical significance of the relationships between (a) the most abundant metabolites (Subset 1) on the corresponding top 15 key genera, and (b) differentially abundant metabolites (Subset 2) in the cancer versus control comparison on the corresponding top 15 key genera, identified via B-MASTER analysis. Progressively larger circles are associated with smaller Bayesian *P* value.

Beyond identifying overall master predictors, we examine key genera affecting two biologically important metabolite subsets. Subset 1 comprises the 10 most abundant metabolites (Yachida *et al.* 2019): *Propionate*, *Butyrate*, *Dihydrouracil*, *Glutamate*, *Urea*, *Succinate*, *5-Aminovalerate*, *Valerate*, *Lysine*, and *Alanine*. Subset 2 includes 11 cancer-associated metabolites (Yachida *et al.* 2019): *X_DCA*, *Glycocholate*, *Taurocholate*, *Isovalerate*, *L-Isoleucine*, *L-Leucine*, *L-Valine*, *L-Phenylalanine*, *L-Tyrosine*, *L-Serine*, and *Glycine*. For each subset, the top 15 master predictor genera were identified, and their directional effects are summarized in Fig. 8a and b, with full results in Tables S14 and S15, available as supplementary data at *Bioinformatics* online. Canonical correlations with cumulatively included predictors are shown in Fig. S11.

For Subset 1, *Veillonella* (rank 1) emerges as a dominant regulator, negatively associated with multiple SCFAs and amino acids while positively linked to *Dihydrouracil* and *Urea*, suggesting a shift toward nitrogen metabolism (Mashima *et al.* 2021, Zhang *et al.* 2023). *Oliverpabstia* (rank 2) and *Merdicola* (rank 3) display similar mixed patterns, with reduced SCFAs but increased nitrogen-related metabolites. *Sellimonas* (rank 4) shows broadly positive effects, whereas *Avimicrobium* (rank 5) positively influences amino acids but negatively associates with *Succinate*. *UBA4372* (rank 6) exhibits reduced SCFAs and increased nitrogen metabolites (Xu *et al.* 2024). *Mogibacterium* and *Lancefieldella* (ranks 8–9) are positively linked to amino acid metabolism, while *Turicibacter*, *Intestinibacter*, and *Anaerotignum* show predominantly negative associations, suggesting suppressive roles in host-associated metabolic pathways.

For Subset 2, a more systemic regulatory pattern emerges, with many genera influencing nearly all cancer-associated metabolites. *Gemmiger* (rank 1) acts as a dominant positive regulator across bile acids and amino acids, including *Glycocholate*, *Taurocholate*, *L-Isoleucine*, *L-Leucine*, *L-Valine*, *L-Phenylalanine*, *L-Tyrosine*, *L-Serine*, and *Glycine*, while uniquely showing a negative association with *X_DCA*. *Ligilactobacillus* (rank 2) and *Faecalimonas* (rank 3) display similarly broad positive effects. In contrast, *Bacteroides* (rank 4) and *CAG.83* (rank 7) exhibit consistent negative associations across bile acids and amino acids. *Agathobacter* (rank 5) and *Caccousia* (rank 6) are uniformly positive, whereas *Anaerotignum* (rank 8) shows widespread negative effects, particularly on branched-chain amino acids. Mixed profiles are observed for *Flavonifractor* (rank 9) and *Lachnospira* (rank 14). Strong positive regulators include *Evtepia* (rank 12) and *Merdicola* (rank 15), while *Amedibacterium*, *1XD42.69*, and *Choladocola* (ranks 10–13) are predominantly negative.

Overall, Subset 1 highlights targeted modulation of SCFA and amino acid pathways, whereas Subset 2 reveals broad regulators of CRC-associated metabolites. To our knowledge, this represents the first genus-level characterization of such coordinated microbiome-metabolite regulation in CRC, extending beyond higher taxonomic analyses (Dueholm *et al.* 2024).

As an additional disease-stratified analysis, Supplementary Section 5.4, available as supplementary data at *Bioinformatics* online applies B-MASTER to the healthy control samples (n=127), using the same posterior selection rule and FIS definition, and compares the resulting master-predictor structures with the CRC cohort. The comparison reveals limited overlap between CRC and healthy top-ranked master predictors, suggesting that the inferred microbiome-metabolite regulatory architecture is largely disease-specific rather than reflecting generic high-abundance microbial effects. In particular, the analysis identifies CRC-enriched and healthy-enriched genera through differential FIS values and percentile rank shifts, while defining target sets consistently as the metabolites selected for each genus under the posterior selection rule (see Fig. S12 and Table S16).

## 6. Discussion

In this article, we introduce B-MASTER, a fully Bayesian framework for identifying essential regressors in multivariate regression via an efficient and scalable Gibbs sampler. Empirically, B-MASTER outperforms existing approaches while maintaining appropriate sparsity and strong signal recovery. Most notably, it scales effectively in high-dimensional settings: unlike remMap and mSSL, which failed to converge for P=287,Q=249, B-MASTER remained stable even for models with up to 4 million parameters (P=Q=2000). Its runtime is nearly invariant to sample size and is observed to increase linearly with the number of parameters. These computational properties, combined with theoretical guarantees of posterior consistency and minimax-rate contraction under sparsity, make B-MASTER well suited for ultra-high-dimensional microbiome-metabolome analysis. While discrete selection priors such as spike-and-slab offer explicit posterior inclusion probabilities, they typically lead to substantially more complex posterior exploration and increased computational burden in large-scale multivariate settings. B-MASTER instead employs a continuous shrinkage formulation that preserves strong sparsity-inducing behavior while enabling efficient Gibbs sampling and scalable uncertainty quantification. Future work could investigate spike-and-slab (George and McCulloch 1993) extensions of B-MASTER for settings where explicit inclusion probabilities are of primary interest, as well as nonlinear or distributionally robust extensions based on spline, kernel, or non-Gaussian response formulations to accommodate complex predictor–response relationships and metabolite distributions encountered in biological systems.

Using the B-MASTER framework, we identify key components of the microbiome that influence overall metabolite profiles. Specifically, we determine the top 50 “master predictors” of gut microbiota in regulating the microbiome-metabolite dependence structure based on colorectal cancer data (Yachida *et al.* 2019). Furthermore, we identify a set of key genera that influence the most abundant metabolites. Our findings align with existing studies in this area. In addition, we examine the set of genera influencing differentially abundant metabolites in cancer versus control cases. Notably, we observe a distinctive regulatory pattern where key genera consistently influence these differential metabolites either positively or negatively. Aligning our results with the findings of Yachida *et al.* (2019), we conclude that elevated levels of *Ligilactobacillus*, *Faecalimonas*, *Agathobacter*, *Caccousia*, *Evtepia*, and *Merdicola* are positively associated with colorectal disease progression. This study represents the first attempt to elucidate the role of key microbiome components at the genus level potentially associated with colorectal cancer by exploring their influence on differentially abundant metabolites of colorectal cancer patients across various stages. We hope that our novel findings provide a foundational basis for further studies on modulating these identified genera as potential targets for colorectal cancer treatment or prevention in the future.

## Supplementary Material

btag500_Supplementary_Data

## Data Availability

The data analyzed in this study are publicly available and were obtained from the colorectal neoplasia cohort originally reported by Yachida *et al.* (2019), comprising subjects undergoing total colonoscopy at the National Cancer Center Hospital, Tokyo, Japan.

## References

[btag500-B1] Aitchison J. The statistical analysis of compositional data. J R Stat Soc 1982;44:139–60.

[btag500-B2] Belkaid Y , Hand W. Role of the microbiota in immunity and inflammation. Cell 2014;157:121–41. 24679531 10.1016/j.cell.2014.03.011 PMC4056765

[btag500-B3] Caporaso JG , Lauber CL, Walters WA et al Global patterns of 16s rrna diversity at a depth of millions of sequences per sample. Proc Natl Acad Sci 2011;108:4516–22. 20534432 10.1073/pnas.1000080107 PMC3063599

[btag500-B4] Chakraborty A , Bhattacharya A, Mallick B. Bayesian sparse multiple regression for simultaneous rank reduction and variable selection. Biometrika 2020;107:205–21. 33100350 10.1093/biomet/asz056 PMC7584295

[btag500-B5] Davar D , Zarour H. Facts and hopes for gut microbiota interventions in cancer immunotherapy. Clin Cancer Res 2022;28:4370–84. 35748749 10.1158/1078-0432.CCR-21-1129 PMC9561605

[btag500-B6] Deshpande SK , Ročková V, George EI. Simultaneous variable and covariance selection with the multivariate spike-and-slab lasso. J. Comput. Graph 2019;28:921–31.

[btag500-B7] Dueholm MKD , Andersen KS, Korntved A-KC et al Midas 5: global diversity of bacteria and archaea in anaerobic digesters. Nat Commun 2024;15:5361. 38918384 10.1038/s41467-024-49641-y PMC11199495

[btag500-B8] Garrett S. Cancer and the microbiota. Science 2015;348:80–6. 25838377 10.1126/science.aaa4972 PMC5535753

[btag500-B9] George E , McCulloch R. Variable selection via gibbs sampling. J Am Stat Assoc 1993;88:881–9.

[btag500-B10] Hoerl A , Kennard R. Ridge regression: biased estimation for nonorthogonal problems. Technometrics 1970;12:55–67.

[btag500-B11] Kyung M , Gill J, Ghosh M. Penalized regression, standard errors, and bayesian lasso. Bayesian Anal 2010;5:369–411.

[btag500-B12] Li H. Microbiome, metagenomics, and high-dimensional compositional data analysis. Annu Rev Stat Appl 2015;2:73–94.

[btag500-B13] Liquet B , Mengersen K, Pettitt AN et al Bayesian variable selection regression of multivariate responses for group data. Bayesian Anal 2017;12:1039–67.

[btag500-B14] Liu J , Tan Y, Cheng H et al Functions of gut microbiota metabolites, current status and future perspectives. Aging Dis 2022;13:1106–26. 35855347 10.14336/AD.2022.0104 PMC9286904

[btag500-B15] Lu Y , Hui F, Zhou G et al MicrobiomeNet: exploring microbial associations and metabolic profiles for mechanistic insights. Nucleic Acids Res 2025;53:D789–96. 39441071 10.1093/nar/gkae944 PMC11701532

[btag500-B16] Mashima I , Liao Y-C, Lin C-H et al Comparative pan-genome analysis of oral *veillonella* species. Microorganisms 2021;9:1175. 34442854 10.3390/microorganisms9081775 PMC8400620

[btag500-B17] MathWorks 2024. Quick start parallel computing in MATLAB. Accessed: 2024-11-19.

[btag500-B18] Muller E , Algavi YM, Borenstein E. The gut microbiome-metabolome dataset collection: a curated resource for integrative meta-analysis. NPJ Biofilms Microbiomes 2022;8:79. 36243731 10.1038/s41522-022-00345-5 PMC9569371

[btag500-B19] Park T , Casella G. The bayesian lasso. J Am Stat Assoc 2008;103:681–6.

[btag500-B20] Parker BJ , Wearsch PA, Veloo ACM et al The genus alistipes: gut bacteria with emerging implications to inflammation, cancer, and mental health. Front Immunol 2020;11:906. 32582143 10.3389/fimmu.2020.00906 PMC7296073

[btag500-B21] Peng J , Wang P, Zhou N et al Partial correlation estimation by joint sparse regression models. J Am Stat Assoc 2009;104:735–46. 19881892 10.1198/jasa.2009.0126 PMC2770199

[btag500-B22] Peng J , Zhu J, Bergamaschi A et al Regularized multivariate regression for identifying master predictors with application to integrative genomics study of breast cancer. Ann Appl Stat 2010;4:53–77. 24489618 10.1214/09-AOAS271SUPP PMC3905690

[btag500-B23] Qian J , Tanigawa Y, Du W et al A fast and scalable framework for large-scale and ultrahigh-dimensional sparse regression with application to the UK biobank. PLoS Genet 2020;16:e1009141. 33095761 10.1371/journal.pgen.1009141 PMC7641476

[btag500-B24] Rockova V , George E. The spike-and-slab lasso. J Am Stat Assoc 2018;113:431–44.

[btag500-B25] Song Y , Chen K, Lv L et al Uncovering the biogeography of the microbial commmunity and its association with nutrient metabolism in the intestinal tract using a pig model. Front Nutr 2022;9:1003763. 36238459 10.3389/fnut.2022.1003763 PMC9552906

[btag500-B26] Swift D , Cresswell K, Johnson R et al A review of normalization and differential abundance methods for microbiome counts data. Wiley Interdiscip Rev 2023;15:e1586.

[btag500-B27] Tibshirani R. Regression shrinkage and selection via the lasso. J R Stat Soc B 1996;58:267–88.

[btag500-B28] Xu X , Ghosh M. Bayesian variable selection and estimation for group lasso. Bayesian Anal 2015;10:909–36.

[btag500-B29] Xu Z , Knight R. Dietary effects on human gut microbiome diversity. Brit J Nutr 2015;113:S1–5. 10.1017/S0007114514004127 PMC4405705 25498959

[btag500-B30] Xu Y , Feng T, Ding Z et al Age-related compositional and functional changes in the adult and breastfed buffalo rumen microbiome. Front Microbiol 2024;15:1342804. 38881655 10.3389/fmicb.2024.1342804 PMC11177756

[btag500-B31] Yachida S , Mizutani S, Shiroma H et al Metagenomic and metabolomic analyses reveal distinct stage-specific phenotypes of the gut microbiota in colorectal cancer. Nat Med 2019;25:968–76. 31171880 10.1038/s41591-019-0458-7

[btag500-B32] Yuan M , Lin Y. Model selection and estimation in regression with grouped variables. J R Stat Soc B 2006;68:49–67.

[btag500-B33] Zhang S , Huang S. The commensal anaerobe veillonella dispar reprograms its lactate metabolism and short-chain fatty acid production during the stationary phase. Microbiol Spectr 2023;11:e03558–22. 36975840 10.1128/spectrum.03558-22 PMC10100942

[btag500-B34] Zhang W , An Y, Qin X et al Gut microbiota-derived metabolites in colorectal cancer: the bad and the challenges. Front Oncol 2021;11:739648. 34733783 10.3389/fonc.2021.739648 PMC8558397

[btag500-B35] Zhang L , Zi L, Kuang T et al Investigating causal associations among gut microbiota, metabolites, and liver diseases: a mendelian randomization study. Front Endocrinol 2023;14:1159148. 10.3389/fendo.2023.1159148 PMC10354516 37476494

[btag500-B36] Zou H , Hastie T. Regularization and variable selection via the elastic net. J R Stat Soc B 2005;67:301–20.

