# Supplementary material for: B-MASTER: scalable Bayesian multivariate regression for master predictor discovery in colorectal cancer microbiome-metabolite profiles
Source: Bioinformatics. 2026 Aug 10;42(8):btag500. doi: 10.1093/bioinformatics/btag500 (PMC13461372; doi:10.1093/bioinformatics/btag500)
Supplement: Supplementary file 1 [file btag500_supplementary_data.pdf]

Supplementary Material for "B-MASTER: Scalable Bayesian Multivariate Regression for Master Predictor Discovery in Colorectal Cancer Microbiome-Metabolite Profiles"

Priyam Das
Department of Biostatistics, Virginia Commonwealth University

Tanujit Dey
Center for Surgery and Public Health, Brigham and Women's Hospital,
Harvard Medical School

Christine B. Peterson
Department of Statistics, Rice University

and

Sounak Chakraborty
Department of Statistics, University of Missouri

Contents

S1 Model, Full Posterior and Gibbs Sampling **3**

S1.1 Comparison with Elastic Net 3

S1.2 Scalability: B-MASTER vs remMap 4

S1.3 Posterior Distribution 5

S1.4 Gibbs Sampling 5

S1.5 Prior on Tuning Parameters and Gibbs Sampling 6

S2 Theoretical results and proofs **8**

S2.1 Main contraction theorem (diagonal Σ_0) 13

S2.2 Correlated errors: sparse-precision extension 15

S2.3 Misspecification robustness . . . 16
S2.4 Row-support (master-predictor) recovery . . . 18

S3 Simulation Studies **19**
S3.1 Real-data Based Simulation Study . . . 20
S3.2 Evaluation of B-MASTER in Higher Dimensions . . . 24

S4 Convergence Diagnostics and Hyperparameter Sensitivity **28**
S4.1 B-MASTER Convergence Diagnostics . . . 28
S4.2 Sensitivity Analysis for Hyper–Priors . . . 30
S4.3 Sensitivity to MCMC Iteration Length . . . 31

S5 B-MASTER Case Study **36**
S5.1 Descriptive statistics and diagnostics . . . 36
S5.2 Fractional Influence Score (FIS) . . . 40
S5.3 Case Study Inference . . . 48
S5.4 Disease-stratified comparison of master predictors . . . 52

Notation

For convenience, we summarize here the key notation used throughout the Supplement.

Symbol	Description
$X \in \mathbb{R}^{N\times P}$	Design matrix with N subjects and P predictors
$Y \in \mathbb{R}^{N\times Q}$	Response matrix with Q outcomes
$B = [\beta_{pq}] \in \mathbb{R}^{P\times Q}$	Regression coefficient matrix
$\varepsilon_i \sim \mathcal{N}_Q(0, \Sigma)$	Error term for subject i
$\Sigma \in \mathbb{R}^{Q\times Q}$	Error covariance matrix
T^2, G^2	Local shrinkage parameters for rows and groups, respectively
τ^2_{pq}	Local element–specific shrinkage parameter for β_{pq}
γ^2_p	Group–specific shrinkage parameter for predictor p
λ_1, λ_2	Global shrinkage hyperparameters
s	Sparsity level (number of nonzero entries in B)
$\|\cdot\|_F$	Frobenius norm (square root of the sum of squared entries)
$\|\cdot\|_{\text{op}}$	Operator norm (largest singular value)

Table S1: Key notation used in the Supplement.

S1 Model, Full Posterior and Gibbs Sampling

S1.1 Comparison with Elastic Net

At first glance, the conditional prior in equation (3) of the main draft may appear similar to the Elastic Net penalty (Zou & Hastie 2005), which also combines l_1- and l_2-type regularization. However, there are key differences. First, the Elastic Net applies an l_1 penalty on individual coefficients and an l_2 penalty on the entire coefficient vector, whereas our formulation applies the l_2 penalty at the level of predictor-specific coefficient vectors $(\beta_{p1}, \ldots, \beta_{pQ})$. This design induces selection of predictors with shared effects across multiple responses, a property not inherent to the Elastic Net. Second, our Bayesian formulation introduces hierarchical priors on the tuning parameters (λ_1, λ_2), allowing them to be adaptively learned from the data rather than fixed by cross-validation. Taken together, these differences demonstrate that the B-MASTER prior is tailored to multivariate response settings with structured sparsity, rather than being a direct analogue of Elastic Net penalization.

S1.2 Scalability: B-MASTER vs remMap

It is important to note several caveats regarding analogous frequentist approaches for optimizing the coefficient matrix $\boldsymbol{B}$ within a penalized likelihood framework, as discussed in (Peng et al. 2010; referred to as 'remMap'). First, the remMap method uses the "active-shooting" algorithm (Friedman et al. 2008, Peng et al. 2009) to optimize $\boldsymbol{B}$ for a given λ_1, with computational complexity $O(NPQ)$. As sample size increases, this results in substantial computational burden. In contrast, the proposed Gibbs sampler algorithm maintains nearly constant computation time, as only a small portion involves dataset-related operations efficiently handled by MATLAB's multi-threading. As N grows, higher levels of multi-threading mitigate computation time increases (MathWorks 2024). For fixed P and Q, the Gibbs sampler's computation time remains largely invariant with sample size. Figure 5 (in the main draft; see Section S3.2 for data generation) shows that remMap's computation time depends strongly on sample size, while B-MASTER exhibits near invariance. Second, selecting an optimal λ_1 via cross-validation intensifies computational demands. If uncertainty quantification is required, bootstrapping may result in prohibitively long computation times. A detailed examination of remMap's performance limitations in high-dimensional settings is provided in the simulation study section. These challenges can be alleviated using a Bayesian framework, where model estimation occurs once, yielding both optimal coefficients and their uncertainty measures. Lastly, the computational time per iteration of the proposed Gibbs sampler is independent of dataset characteristics, assuming P and Q are fixed. In contrast, remMap's "active-shooting" algorithm exhibits strong data dependence, with convergence time varying significantly across datasets of the same dimensionality. For instance, as shown in Figure 5 (left) (main draft), with $P = Q = 40$, the computation time for $N = 40$ exceeded that for $N = 200$, and for $P, Q = 50$, $N = 50$. B-MASTER, in contrast, shows predictable scaling (Figure 5 (right) in the main draft). It is observed that B-MASTER takes slightly more time for $N = P$ scenarios compared to $N = 5P$ and $N = 10P$ scenarios, likely due to increased multi-threading activation in MATLAB for high-dimensional matrix operations (MathWorks 2024).

S1.3 Posterior Distribution

We introduce latent parameters $\boldsymbol{T}^2 = (\boldsymbol{\tau}_1^2, \ldots, \boldsymbol{\tau}_P^2)^T = \left(\tau_{pq}^2\right)_{P\times Q}$ and $\boldsymbol{G}^2 = \left(\gamma_p^2\right)_{P\times 1}$. This formulation yields the following hierarchical representation of the B-MASTER prior (Xu & Ghosh 2015):

$$\boldsymbol{\beta}^{(p)}|\boldsymbol{\tau}_p^2, \gamma_p^2, \sigma_1^2, \ldots, \sigma_Q^2 \sim N_Q(\mathbf{0}, \boldsymbol{V}_p),\ \boldsymbol{V}_p = diag\left\{\sigma_q^2\left(\frac{1}{\tau_{pq}^2} + \frac{1}{\gamma_p^2}\right)^{-1}\right\} \text{ for } q = 1, \ldots, Q,$$

$$\pi(\boldsymbol{\tau}_p^2, \gamma_p^2) \propto C_p^*(\lambda_1^2, \lambda_2^2)\prod_{q=1}^{Q}\left[(\tau_{pq}^2)^{-1/2}\left(\frac{1}{\tau_{pq}^2} + \frac{1}{\gamma_p^2}\right)^{-1/2}\right](\gamma_p^2)^{-1/2}\exp\left\{-\frac{\lambda_1^2}{2}\sum_{q=1}^{Q}\tau_{pq}^2 - \frac{\lambda_2^2}{2}\gamma_p^2\right\} \tag{1}$$

where $C_p^*(\lambda_1^2, \lambda_2^2)$ is a normalizing constant involving both penalty terms. It is introduced solely to ensure propriety of the hierarchical prior representation. The propriety of the prior distribution introduced in (1) is established in Lemma S2.1. We put a non-informative prior on σ_q^2, $\pi(\sigma_q^2) \propto \frac{1}{\sigma_q^2}$, for $q = 1, \ldots, Q$. Hereafter, combining the likelihood from the model (1) of the main paper along with the aforementioned prior setup, we obtain the joint posterior distribution:

$$\begin{aligned}\pi(\boldsymbol{B}, \boldsymbol{T}^2, \boldsymbol{G}^2, \boldsymbol{\sigma}^2, |\ \boldsymbol{X}, \boldsymbol{Y}) \propto &\prod_{q=1}^{Q}\left[(\sigma_q^2)^{-\frac{(N+P)}{2}-1}\exp\left\{-\frac{(\boldsymbol{y}_q - \boldsymbol{X}\boldsymbol{\beta}_q)'(\boldsymbol{y}_q - \boldsymbol{X}\boldsymbol{\beta}_q) + \boldsymbol{\beta}_q' D_{\tau_q}\boldsymbol{\beta}_q}{2\sigma_q^2}\right\}\right]. \\ &\prod_{p=1}^{P}\left[C_p^*(\lambda_1^2, \lambda_2^2)\prod_{q=1}^{Q}\left\{(\tau_{pq}^2)^{-1/2}\left(\frac{1}{\tau_{pq}^2} + \frac{1}{\gamma_p^2}\right)^{-1/2}\right\}(\gamma_p^2)^{-1/2}\right]. \\ &\exp\left\{-\frac{\lambda_1^2}{2}\sum_{q=1}^{Q}\tau_{pq}^2 - \frac{\lambda_2^2}{2}\gamma_p^2\right\},\end{aligned} \tag{2}$$

where $\boldsymbol{y}_q = (y_q^1, y_q^2, \ldots, y_q^N)$ denotes the q-th component (column) of the response matrix $\boldsymbol{Y}$ ($N \times Q$ matrix of response); $\boldsymbol{x}^i = (x_1^i, x_2^i, \ldots, x_P^i)$ denotes the i-th row of the covariate matrix $\boldsymbol{X}$ ($N \times P$ matrix of covariates); and D_{τ_q} is a $P \times P$ diagonal matrix such that $D_{\tau_q}(p,p) = \frac{1}{\tau_{pq}^2} + \frac{1}{\gamma_p^2}$ for $p = 1, \ldots, P$.

S1.4 Gibbs Sampling

From the joint posterior distribution (2), we derive a Gibbs sampling algorithm to generate posterior samples of $\boldsymbol{B} = \left(\beta_{pq}\right)_{P\times Q}$, $\boldsymbol{T}^2 = (\boldsymbol{\tau}_1^2, \ldots, \boldsymbol{\tau}_P^2)^T = \left(\tau_{pq}^2\right)_{P\times Q}$, $\boldsymbol{G}^2 = \left(\gamma_p^2\right)_{P\times 1}$, and $\boldsymbol{\sigma}^2 = (\sigma_1^2, \sigma_2^2, \ldots, \sigma_Q^2)$ from their full conditional posteriors as follows,

- Sample $\boldsymbol{\beta}_q = (\beta_{1q}, \ldots, \beta_{Pq})^T$ for $q = 1, \ldots, Q$, from

$$\boldsymbol{\beta}_q | \boldsymbol{T}^2, \boldsymbol{G}^2, \sigma_q^2 \sim N_p\left((\boldsymbol{X}'\boldsymbol{X} + D_{\tau_q})^{-1}\boldsymbol{X}'\boldsymbol{y}_q, \sigma_q^2(\boldsymbol{X}'\boldsymbol{X} + D_{\tau_q})^{-1}\right).$$

- Sample τ_{pq}^2, for $p = 1, \ldots, P; q = 1, \ldots, Q$, such that $\tau_{pq}^2 = \frac{1}{\delta_{pq}^{(1)}}$, where

$$\delta_{pq}^{(1)} | \beta_{pq}, \sigma_q^2, \lambda_1^2 \sim \text{Inv-Gaussian}\left(\sqrt{\frac{\lambda_1^2\sigma_q^2}{\beta_{pq}^2}}, \lambda_1^2\right).$$

- Sample γ_p^2 for $p = 1, \ldots, P$, such that $\gamma_p^2 = \frac{1}{\delta_p^{(2)}}$, where

$$\delta_p^{(2)} | \boldsymbol{B}, \boldsymbol{\sigma}^2, \lambda_2^2 \sim \text{Inv-Gaussian}\left(\sqrt{\frac{\lambda_2^2}{\sum_{q=1}^{Q}(\beta_{pq}^2/\sigma_q^2)}}, \lambda_2^2\right).$$

- Sample σ_q^2 for $q = 1, \ldots, Q$, from

$$\sigma_q^2 | \boldsymbol{\beta}_q, \boldsymbol{T}^2, \boldsymbol{G}^2 \sim \text{Inv-Gamma}\left(\frac{N+P}{2}, \frac{(\boldsymbol{y}_q - \boldsymbol{X}\boldsymbol{\beta}_q)'(\boldsymbol{y}_q - \boldsymbol{X}\boldsymbol{\beta}_q) + \boldsymbol{\beta}_q' D_{\tau_q}\boldsymbol{\beta}_q}{2}\right).$$

Note that the tuning parameters in (2) are incorporated as fixed values. Thus, this proposed sampling scheme can be used to perform posterior inference of B-MASTER parameters for given fixed values of the tuning parameters λ_1 and λ_2. We further delve into putting appropriate hyper-prior on the prior parameters in the following sub-section.

S1.5 Prior on Tuning Parameters and Gibbs Sampling

The Gibbs sampling scheme outlined in the previous subsection is conceptually analogous to the sparse model estimation process within the frequentist framework. In the frequentist setting, for fixed values of the tuning parameters, the model is fitted, and optimal tuning parameter values are subsequently selected through methods such as cross-validation or approaches based on Stein's unbiased risk estimate (Tibshirani 1996). In contrast, the Bayesian framework circumvents this extended cross-validation process by assigning suitable hyper-priors to λ_1 and λ_2 (Park & Casella 2008, Kyung et al. 2010, Roy & Chakraborty 2017). This facilitates a fast and efficient mechanism for sampling λ_1 and λ_2 simultaneously

with other model parameters. Following the recommendations of Kyung et al. (2010) and Xu & Ghosh (2015), we assign the following joint prior distribution to λ_1^2 and λ_2^2:

$$\pi(\lambda_1^2, \lambda_2^2) \propto C(\lambda_1^2, \lambda_2^2)(\lambda_1^2)^{a_1} \exp\left(-b_1\lambda_1^2\right)(\lambda_2^2)^{a_2} \exp -(b_2\lambda_2^2), \tag{3}$$

where $C(\lambda_1^2, \lambda_2^2) = \left(\prod_{p=1}^{P} C_p^*(\lambda_1^2, \lambda_2^2)\right)^{-1}$ is the induced normalizing constant from the hierarchical prior construction, and a_1, b_1, a_2, b_2 are some positive constants.

Such a prior structure is well-studied in the context of Bayesian group lasso and sparse graphical models (Xu & Ghosh 2015, Das et al. 2020). Following their recommendation, to impose a stronger penalization structure as the total number of regression coefficients increases, hyper-prior parameter values are chosen as $a_1 = PQ + r_1$, $a_2 = \frac{PQ}{2} + r_2$, $b_1 = \frac{1}{2}\sum_{p=1}^{P}\sum_{q=1}^{Q}\tau_{pq}^2 + \delta_1$ and $b_2 = \frac{1}{2}\sum_{p=1}^{P}\gamma_p^2 + \delta_2$, where $r_1, r_2, \delta_1, \delta_2$ are positive constants. In B-MASTER analyses, we set $r_1 = r_2 = 1$ and $\delta_1 = \delta_2 = 0.1$. This prior structure is found to perform well across a broad range of P and Q values, maintaining excellent control over the true positive rate (TPR) and false positive rate (FPR), without issues in both low and high-dimensional scenarios, as detailed in the simulation study section of the main paper. The propriety of the prior introduced in (3) is discussed in detail in Lemma S2.1. The conditional posterior distributions of the tuning parameters are given as follows:

$$\lambda_1^2|\boldsymbol{T}^2 \sim \text{Gamma}\left(PQ + r_1, \frac{1}{2}\sum_{p,q}\tau_{pq}^2 + \delta_1\right), \tag{4}$$

$$\lambda_2^2|\boldsymbol{G}^2 \sim \text{Gamma}\left(\frac{PQ}{2} + r_2, \frac{1}{2}\sum_{p=1}^{P}\gamma_p^2 + \delta_2\right). \tag{5}$$

Note that the full conditional posterior distributions of λ_1^2 and λ_2^2 do not involve any computation of $C(\lambda_1^2, \lambda_2^2)$ resulting in substantial improvement in computational efficiency. By integrating the sampling steps for λ_1^2 and λ_2^2 outlined above with the Gibbs sampling procedure detailed in Section S1.4, the complete Gibbs sampling framework for B-MASTER is established.

S2 Theoretical results and proofs

Throughout, let $X \in \mathbb{R}^{N\times P}$ collect the row vectors $x_i^\top$, $Y \in \mathbb{R}^{N\times Q}$ collect $y_i^\top$, and consider

$$y_i \;=\; x_i B_0 + \varepsilon_i, \qquad \varepsilon_i \overset{\text{i.i.d.}}{\sim} \mathcal{N}_Q(0, \Sigma_0), \quad i = 1, \ldots, N,$$

where $B_0 \in \mathbb{R}^{P\times Q}$ is the true coefficient matrix and $\Sigma_0 \in \mathbb{R}^{Q\times Q}$ the true error covariance. Denote by $\|\cdot\|_F$ the Frobenius norm and by $\|\cdot\|_{\text{op}}$ the operator norm. For a matrix $B = [\beta_{pq}]$, define its sparsity level

$$s \;:=\; \#\{(p,q) : \beta_{pq} \neq 0\}.$$

We adopt the continuous shrinkage prior of the main text together with Gamma hyperpriors on $(\lambda_1^2, \lambda_2^2)$; for the error scales we keep the implementation's prior (see (A5)(ii)).

Status of assumptions in B-MASTER. The implementation fits the Gaussian multivariate regression with a *diagonal* working covariance $\Sigma = \text{diag}(\sigma_1^2, \ldots, \sigma_Q^2)$, Jeffreys priors $\pi(\sigma_q^2) \propto 1/\sigma_q^2$, and the continuous shrinkage prior on B from (A5)(i). Under this setup:

- *Directly applicable to the implementation.* Lemma S2.1 (posterior propriety and $N^{-1/2}$ concentration of $\{\sigma_q^2\}$) and Theorem S2.3 (posterior contraction for B at rate $\delta_N = \sqrt{s\log(PQ)/N}$) both apply under (A1)–(A3),(A5)(i)–(ii). The row-support recovery result, Theorem S2.8, also applies under the same diagonal-Σ conditions (with the stated beta-min requirement for exact selection).

- *Theory-only robustness without changing the sampler.* If the *true* errors are not Gaussian/diagonal but are mean-zero sub-Gaussian with covariance Σ_0, Theorem S2.5 shows that the B-MASTER posterior for B still contracts at the same rate around the KL projection $B^\star$ of the true model onto the working Gaussian family; the Gibbs sampler is unchanged.

- *Alternative model requiring a different prior/sampler.* The correlated-error result, Theorem S2.4, assumes a *sparse precision* prior on $\Omega = \Sigma^{-1}$ (A5)(iii) and yields joint contraction for (B, Ω). This is not the model currently implemented (which fixes Ω to be diagonal), so Theorem S2.4 does *not* directly describe the output of the present

sampler; it applies if one augments the model to include Ω and samples (or integrates) it.

All results rely on standard design regularity (A1) and the sparsity regime $s\log(PQ) = o(N)$. The diagonal-Σ_0 case (A3) matches the implementation; (A4) is only invoked in the correlated-error extension.

Assumptions.

- (A1) **Design condition (compatibility/Restricted Eigenvalue).** Let $G_N := X^\top X/N$. There exists $\kappa > 0$ such that for all matrices $\Delta \in \mathbb{R}^{P\times Q}$ obeying $\operatorname{supp}(\Delta) \subseteq S$ with $|S| \le s + s'$ (for some $s' = o(N)$),

$$\frac{1}{N}\sum_{q=1}^{Q} \|X\Delta_{\cdot q}\|_2^2 \;\ge\; \kappa\|\Delta\|_F^2.$$

  This holds, for example, if $\lambda_{\min}(G_N) \ge \kappa$ or under standard restricted eigenvalue conditions used in high-dimensional regression. In addition, we assume a uniform upper spectral bound on the Gram matrix: $\|G_N\|_{\mathrm{op}} \le \bar{\kappa} < \infty$ (e.g., after column standardization).

- (A2) **Sparsity and growth.** Define

$$\delta_N \;:=\; \sqrt{\frac{s\,\log(PQ)}{N}}.$$

  We assume the product condition $s\,\log(PQ) = o(N)$ (equivalently, $\delta_N \to 0$). This allows P and Q to grow with N; e.g., if $\log(PQ) \asymp \log N$ it suffices that $s = o(N/\log N)$.

- (A3) **Error covariance (diagonal case).** In Theorem S2.3 we assume $\Sigma_0 = \operatorname{diag}(\sigma_{01}^2, \dots, \sigma_{0Q}^2)$ with $0 < \underline{\sigma}^2 \le \sigma_{0q}^2 \le \overline{\sigma}^2 < \infty$.

- (A4) **Error covariance (general case).** In Theorem S2.4 we allow general $\Sigma_0 \in \mathbb{S}_{++}^Q$ with eigenvalues bounded between positive constants and *sparse precision* $\Omega_0 := \Sigma_0^{-1}$:

$$s_\Omega \;:=\; \#\{(j,k) : j<k,\ (\Omega_0)_{jk} \neq 0\} \qquad \text{with} \quad s_\Omega = o\left(\frac{N}{\log Q}\right).$$

- (A5) **Priors.**

  (i) *Coefficient prior.* Conditionally on $\sigma_1^2, \dots, \sigma_Q^2$, the prior density on B is

  $$\pi(B \mid \sigma_1^2, \dots, \sigma_Q^2) \;\propto\; \exp\Big[-\lambda_1 \sum_{p,q} \frac{|\beta_{pq}|}{2\sigma_q^2} - \lambda_2 \sum_{p=1}^{P} \Big\{\sum_{q=1}^{Q} \frac{\beta_{pq}^2}{2\sigma_q^2}\Big\}^{1/2}\Big],$$

  with independent hyperpriors $\lambda_1^2, \lambda_2^2 \sim \mathrm{Gamma}(a_j, b_j)$, $a_j, b_j \in [c_-, c_+]$ fixed constants. This prior is proper and has exponentially decaying tails.

  (ii) *Error variances (implementation prior).* For each q, the sampler uses the improper Jeffreys prior $\pi(\sigma_q^2) \propto 1/\sigma_q^2$. Lemma S2.1 shows the resulting posterior in $(B, \sigma_1^2, \dots, \sigma_Q^2)$ is proper under mild conditions. All contraction proofs for B go through for this choice and, more generally, for any proper heavy-tailed scale prior (e.g., half-t_ν on σ_q or scaled-inverse-χ^2); see Remark S2.2.

  (iii) *Precision matrix (general case).* For Theorem S2.4, we place a sparse precision prior on $\Omega = \Sigma^{-1}$ supported on $\mathbb{S}_{++}^Q$ that yields posterior contraction at rate $\varepsilon_\Omega \asymp \sqrt{s_\Omega \log Q/N}$ in Frobenius norm (e.g., graphical-model–based priors as in Banerjee & Ghosal 2014).

Lemma S2.1 (Posterior propriety and tightness of $\{\sigma_q^2\}$ under Jeffreys)**.** *Assume (A1)–(A3) and a proper coefficient prior (A5)(i). Then, under the improper variance prior* $\pi(\sigma_q^2) \propto 1/\sigma_q^2$ *used in* B-MASTER*:*

(a) The joint posterior $\pi(B, \sigma_1^2, \dots, \sigma_Q^2 \mid X, Y)$ *is proper provided* $N \geq 1$ *and, almost surely,* $\sum_{i=1}^N \|y_i - x_i B\|_2^2 > 0$ *for all* B *in a set of prior probability one (which holds under any nondegenerate data-generating process).*

(b) If, in addition, Σ_0 *is diagonal with eigenvalues bounded as in (A3), then for every* q *and any fixed compact interval* $I_q \ni \sigma_{0q}^2$, $\Pi(\sigma_q^2 \in I_q \mid X, Y) \to 1$ *in* P_{B_0,Σ_0}*-probability as* $N \to \infty$*; in particular, the posterior of* σ_q^2 *is tight and does not affect the rate for* B*.*

Proof of Lemma S2.1. Write $y_q = (y_{1q}, \dots, y_{Nq})^\top$ and $\beta_q := B_{\cdot q} \in \mathbb{R}^P$. Under the model

with diagonal $\Sigma = \mathrm{diag}(\sigma_1^2, \dots, \sigma_Q^2)$, the likelihood factorizes over q:

$$p(Y \mid B, \sigma_1^2, \dots, \sigma_Q^2, X) \;=\; \prod_{q=1}^{Q} (2\pi\sigma_q^2)^{-N/2} \exp\Big(- \tfrac{1}{2\sigma_q^2}\, \mathrm{RSS}_q(B) \Big),$$

where $\mathrm{RSS}_q(B) := \|y_q - X\beta_q\|_2^2$. With the Jeffreys prior $\pi(\sigma_q^2) \propto (\sigma_q^2)^{-1}$ and the coefficient prior $\pi(B \mid \sigma_1^2, \dots, \sigma_Q^2)$ from (A5)(i), the joint posterior kernel is proportional to

$$\prod_{q=1}^{Q} (\sigma_q^2)^{-(N/2+1)} \exp\Big\{ - \tfrac{\mathrm{RSS}_q(B)}{2\sigma_q^2} - \tfrac{\lambda_1}{2} \tfrac{\sum_p |\beta_{pq}|}{\sigma_q^2} - \lambda_2 \sum_{p=1}^{P} \Big(\tfrac{1}{2} \sum_{j=1}^{Q} \tfrac{\beta_{pj}^2}{\sigma_j^2} \Big)^{1/2} \Big\}.$$

(a) Posterior propriety. Fix q. Under (A5)(i), the joint posterior has kernel

$$\pi(B, \sigma^2 \mid X, Y) \;\propto\; \prod_{q=1}^{Q} (\sigma_q^2)^{-(N/2+1)} \exp\Big\{ - \tfrac{\mathrm{RSS}_q(B)}{2\sigma_q^2} - \tfrac{\lambda_1}{2} \tfrac{\sum_p |\beta_{pq}|}{\sigma_q^2} \Big\} \exp\Big\{ -\lambda_2 \sum_{p=1}^{P} \Big(\tfrac{1}{2} \sum_{j=1}^{Q} \tfrac{\beta_{pj}^2}{\sigma_j^2} \Big)^{1/2} \Big\}.$$

For each fixed B, the factor in σ_q^2 is bounded above by

$$(\sigma_q^2)^{-(N/2+1)} \exp\Big(- \tfrac{c_{2q}(B)}{\sigma_q^2} - \tfrac{c_{1q}(B)}{\sigma_q} \Big),$$

with $c_{2q}(B) = \frac{1}{2}\{\mathrm{RSS}_q(B) + \lambda_1 \sum_p |\beta_{pq}|\} \geq 0$ and $c_{1q}(B) = (\lambda_2/\sqrt{2}) \sum_p |\beta_{pq}| \geq 0$, since $\sqrt{\sum_j a_{pj}} \geq \sqrt{a_{pq}}$ for each p. Let $u = \sigma_q^2$. For fixed B the integrand is

$$u^{-(N/2+1)} \exp\Big(- \frac{c_{2q}(B)}{u} - \frac{c_{1q}(B)}{\sqrt{u}} \Big), \qquad u \in (0, \infty).$$

Split the integral at 1. On $(0, 1]$, if $c_{2q}(B) > 0$ then $\exp\{-c_{2q}(B)/u\}$ dominates any polynomial and the integral is finite; if $c_{2q}(B) = 0$ but $c_{1q}(B) > 0$, then $\exp\{-c_{1q}(B)/\sqrt{u}\}$ still ensures integrability at 0. On $[1, \infty)$ the exponential factor is ≤ 1, so $\int_1^\infty u^{-(N/2+1)} du < \infty$ for all $N \geq 1$. Hence the inner integral in $u = \sigma_q^2$ is finite whenever $(c_{1q}(B), c_{2q}(B)) \neq (0, 0)$. The event $c_{1q}(B) = c_{2q}(B) = 0$ requires simultaneously $\beta_{pq} = 0$ for all p and $\mathrm{RSS}_q(B) = 0$, which would imply $y_q = 0$ almost surely under the Gaussian error model. Thus with probability one under the data generating process, the inner integrals in σ_q^2 are finite for every B.

Moreover, standard bounds for the above integrals yield polynomial growth in $\sum_p |\beta_{pq}|$ and $\mathrm{RSS}_q(B)$; because the prior in (A5)(i) has exponentially decaying tails in B (cf. Castillo

et al. 2015), the outer integral over B is finite. Therefore the overall normalizing constant of the posterior is finite.

Let Π_X denote the orthogonal projector onto $\mathcal{C}(X)$. Finally, if $\operatorname{rank}(X) < N$, then $\inf_{\beta_q} \mathrm{RSS}_q(B) = \|(I - \Pi_X)y_q\|_2^2 > 0$ almost surely, which makes the bound immediate. If $\operatorname{rank}(X) = N$, then $\{B : \mathrm{RSS}_q(B) = 0\}$ lies on a lower-dimensional affine set of Lebesgue measure zero, to which the absolutely continuous prior (A5)(i) assigns probability zero. Thus the integrand's singularities are supported on a π-null set, and the Lebesgue integral ignores them. Combining these arguments establishes that the posterior is proper almost surely.

(b) Tightness and $N^{-1/2}$–scale concentration of σ_q^2. Fix q and consider the marginal posterior of σ_q^2 after integrating out B and the other variances:

$$\pi(\sigma_q^2 \mid X, Y) \;\propto\; (\sigma_q^2)^{-(N/2+1)}\, M_q(\sigma_q^2), \qquad M_q(\sigma_q^2) = \int \exp\Big(-\tfrac{1}{2\sigma_q^2}\|y_q - X\beta_q\|_2^2\Big)\pi_q(\beta_q)\, d\beta_q,$$

where π_q is the marginal prior on β_q induced by (A5)(i). The coefficient prior contributes additional exponential terms such as $\exp\{-\lambda_1 \sum_p |\beta_{pq}|/(2\sigma_q^2) - \lambda_2 \sum_p (\sum_j \beta_{pj}^2/\sigma_j^2)^{1/2}\}$, but these are of order $O_p(1)$ uniformly for σ_q^2 in $N^{-1/2}$-neighborhoods of σ_{0q}^2 and therefore do not alter the leading likelihood curvature; cf. the general argument for variance parameters in van der Vaart (1998, Ch. 10).

Let $\widehat{\beta}_q$ be a least-squares minimizer and $\mathrm{RSS}_q^{\min} = \|y_q - X\widehat{\beta}_q\|_2^2 = \|(I - \Pi_X)y_q\|_2^2$. A quadratic expansion around $\widehat{\beta}_q$ gives

$$\|y_q - X\beta_q\|_2^2 = \mathrm{RSS}_q^{\min} + (\beta_q - \widehat{\beta}_q)^\top (X^\top X)(\beta_q - \widehat{\beta}_q).$$

Using (A1) and continuity of π_q, a Laplace approximation for $M_q(\sigma_q^2)$ shows that, up to a random factor C_q bounded away from 0 and ∞ with high probability,

$$\pi(\sigma_q^2 \mid X, Y) \;=\; (1 + o_{P_0}(1))\, (\sigma_q^2)^{-(N/2+1)} \exp\Big(-\tfrac{\mathrm{RSS}_q^{\min}}{2\sigma_q^2}\Big) \cdot C_q.$$

Under the true model with diagonal Σ_0 (A3), $\mathrm{RSS}_q^{\min}$ has distribution $\sigma_{0q}^2\, \chi^2_{N-\operatorname{rank}(X)}$, so $\mathrm{RSS}_q^{\min}/N \to \sigma_{0q}^2$ and $\sqrt{N}(\mathrm{RSS}_q^{\min}/N - \sigma_{0q}^2) = O_{P_0}(1)$. Therefore the log-posterior of σ_q^2

satisfies

$$\ell_q(\sigma_q^2) - \ell_q(\sigma_{0q}^2) = -\tfrac{N}{2}\Big\{ \log(\sigma_q^2/\sigma_{0q}^2) + \sigma_{0q}^2/\sigma_q^2 - 1 \Big\} + O_{P_0}(1),$$

which equals the Kullback–Leibler divergence between $\mathcal{N}(0, \sigma_{0q}^2)$ and $\mathcal{N}(0, \sigma_q^2)$ up to $O(1)$ error (cf. Ghosal et al. 2000). A quadratic expansion of the KL divergence shows that, for some $c > 0$,

$$\log(\sigma_q^2/\sigma_{0q}^2) + \sigma_{0q}^2/\sigma_q^2 - 1 \;\geq\; c\frac{(\sigma_q^2 - \sigma_{0q}^2)^2}{\sigma_{0q}^4} \quad \text{whenever } |\sigma_q^2 - \sigma_{0q}^2| \text{ is small.}$$

Hence the posterior mass outside $|\sigma_q^2 - \sigma_{0q}^2| > M/\sqrt{N}$ decays at rate $\exp(-c'M^2)$ uniformly in M. It follows that

$$\Pi(\sigma_q^2 \in I_q \mid X, Y) \;\to\; 1 \quad \text{for every fixed compact } I_q \ni \sigma_{0q}^2,$$

and the posterior of σ_q^2 concentrates at the $N^{-1/2}$ scale. Since (A3) ensures uniform eigenvalue bounds across q, the collection $\{\sigma_q^2\}$ is tight and their fluctuations are $O_{P_0}(N^{-1/2})$, which does not affect the contraction rate for B. □

Remark S2.2 (On variance priors). Our analysis does not rely on the variance prior being proper. The proofs below only use: (i) posterior propriety (Lemma S2.1); and (ii) that the posterior for $\{\sigma_q^2\}$ is tight in neighborhoods of the true values under (A3). Consequently, the contraction results for B are identical whether one uses the implementation's Jeffreys prior $\pi(\sigma_q^2) \propto 1/\sigma_q^2$ or any proper, heavy-tailed alternative (e.g., half-t/scaled-inverse-χ^2). See also Simpson et al. (2017) for a discussion of robust scale priors.

S2.1 Main contraction theorem (diagonal Σ_0)

Theorem S2.3 (Posterior contraction and consistency (diagonal errors))**.** *Assume* (A1)–(A3) *and* (A5)(i)–(ii)*. Let* $\delta_N := \sqrt{\frac{s\log(PQ)}{N}}$*. Then, for any fixed* $M > 0$*,*

$$\Pi(\, \|B - B_0\|_F \;>\; M\,\delta_N \,|\, X, Y) \;\to\; 0 \quad \textit{in } P_{B_0,\Sigma_0}\textit{-probability as } N \to \infty.$$

In particular, $\Pi(\|B - B_0\|_F > \varepsilon \mid X, Y) \to 0$ *for every fixed* $\varepsilon > 0$ *(posterior consistency).*

Proof. We verify the three standard ingredients for posterior contraction (Ghosal et al.

2000): (i) prior thickness in Kullback–Leibler (KL) neighborhoods; (ii) exponentially powerful tests; (iii) sieve entropy bound.

(i) Prior thickness. Write $\theta = (B, \sigma_1^2, \ldots, \sigma_Q^2, \lambda_1^2, \lambda_2^2)$ and $\theta_0 = (B_0, \sigma_{01}^2, \ldots, \sigma_{0Q}^2, \lambda_{10}^2, \lambda_{20}^2)$, with $(\lambda_{10}^2, \lambda_{20}^2)$ any fixed values in the support of the Gamma hyperpriors. Under (A3), the per–observation Kullback–Leibler divergence between two Gaussian regressions with diagonal covariance satisfies

$$\mathrm{KL}(\theta_0 \,\|\, \theta) \;\lesssim\; \frac{1}{N}\sum_{q=1}^{Q} \frac{\|X(B-B_0)_{\cdot q}\|_2^2}{\sigma_{0q}^2} + C\sum_{q=1}^{Q} \big|\log\sigma_q^2 - \log\sigma_{0q}^2\big| \;\lesssim\; \|B-B_0\|_F^2 + \sum_{q=1}^{Q} \big|\log\sigma_q^2 - \log\sigma_{0q}^2\big|,$$

since $\frac{1}{N}\sum_{q=1}^{Q}\|X(B-B_0)_{\cdot q}\|_2^2 = \sum_{q=1}^{Q}(B-B_0)_{\cdot q}^\top G_N (B-B_0)_{\cdot q} \le \|G_N\|_{\mathrm{op}}\|B-B_0\|_F^2$, and $\|G_N\|_{\mathrm{op}}$ is bounded by (A1). Hence, for any small $\rho > 0$, the set

$$\mathcal{K}_N(\rho) \;:=\; \Big\{(B, \sigma_1^2, \ldots, \sigma_Q^2) : \; \|B-B_0\|_F \le \delta_N, \; \max_{1\le q\le Q}\big|\log\sigma_q^2 - \log\sigma_{0q}^2\big| \le \rho\Big\}$$

is contained in a KL-ball of radius $C\,\delta_N^2$ (per observation), so that the full-sample KL is $\le C\,N\delta_N^2$.

To lower bound the prior mass of $\mathcal{K}_N(\rho)$, first note that (A5)(ii) allows any proper heavy-tailed scale prior on each σ_q^2 (e.g., half-t/scaled-inverse-χ^2) without changing the contraction for B. Under any such proper choice, $\Pi\big(\max_q|\log\sigma_q^2 - \log\sigma_{0q}^2| \le \rho\big) \ge c_\rho > 0$. If Jeffreys' (improper) prior in (A5)(ii) is used, we replace it by a truncated version on $\sigma_q^2 \in I_q$ (a fixed compact interval around σ_{0q}^2) to define a proper prior; Lemma S2.1(a) guarantees the normalizing constant is finite, and the same lower bound holds with a constant independent of N. Since posterior contraction is insensitive to such local truncation, the rate for B is unchanged. For the coefficient matrix, by continuity/positivity of the prior density and standard small-ball bounds for continuous shrinkage around an s-sparse truth,

$$\Pi(\|B-B_0\|_F \le \delta_N) \;\ge\; \exp\{-C_1 N\delta_N^2 - C_2 s\log(1/\delta_N)\},$$

cf. Castillo et al. (2015, Sec. 2). Under polynomial growth of PQ in N (so $\log N \lesssim \log(PQ)$), the second term is absorbed into $N\delta_N^2$, hence $\Pi(\mathcal{K}_N(\rho)) \ge \exp\{-C'' N\delta_N^2\}$. This verifies the prior thickness requirement at rate δ_N.

(ii) Tests. Testing $H_0 : B = B_0$ vs. $H_1 : \|B-B_0\|_F > M\delta_N$ with σ_q^2 in compact

neighborhoods reduces to the log-likelihood ratio

$$\Lambda_N(B) \;=\; \sum_{q=1}^{Q} \frac{1}{2\sigma_{0q}^2}\Big\{\|Y_{\cdot q} - XB_{0,\cdot q}\|_2^2 - \|Y_{\cdot q} - XB_{\cdot q}\|_2^2\Big\}.$$

By Gaussian calculations and (A1), $\mathbb{E}_0\Lambda_N(B) \lesssim -N\|B - B_0\|_F^2$ and $\mathrm{Var}_0(\Lambda_N(B)) \lesssim N\|B - B_0\|_F^2$. The test $\phi_N := \mathbb{I}\{\Lambda_N(B) < -cN\delta_N^2\}$ satisfies $\mathbb{E}_0\phi_N \leq e^{-c_1 N\delta_N^2}$ and $\sup_{\|B-B_0\|_F > M\delta_N} \mathbb{E}_B(1-\phi_N) \leq e^{-c_2 N\delta_N^2}$; see van der Vaart (1998, Ch. 7) and Ghosal et al. (2000).

(iii) Sieve and entropy. Let

$$\mathcal{F}_N \;:=\; \Big\{B \in \mathbb{R}^{P\times Q} :\; \|B\|_\infty \leq M_N,\; \sum_{p=1}^{P} \|\beta_{p\cdot}\|_2 \leq R_N\Big\}.$$

Choose $M_N \asymp \log(PQ)$ and $R_N \asymp s\log(PQ)$. By the exponential tails of (A5)(i) on both entrywise ℓ_1 and rowwise ℓ_2 terms (cf. Castillo et al. 2015, Sec. 2),

$$\Pi(\mathcal{F}_N^c) \;\leq\; \Pi(\|B\|_\infty > M_N) + \Pi\Big(\sum_p \|\beta_{p\cdot}\|_2 > R_N\Big) \;\leq\; \exp\{-C_3\, s\log(PQ)\} \;=\; \exp\{-C_3 N\delta_N^2\}.$$

Moreover, the metric entropy of $\mathcal{F}_N$ under $\|\cdot\|_F$ satisfies

$$\log N(\epsilon, \mathcal{F}_N, \|\cdot\|_F) \;\lesssim\; \frac{R_N^2}{\epsilon^2}\log\big(eP\big) \;+\; s\log\Big(\frac{M_N}{\epsilon}\Big) \;\lesssim\; s\log(PQ) + s\log(1/\epsilon),$$

so with $\epsilon = \delta_N$ and $s\log(PQ) \lesssim N\delta_N^2$ we have $\log N(\delta_N, \mathcal{F}_N, \|\cdot\|_F) \leq C_4 N\delta_N^2$. Combining (i)–(iii) via Ghosal et al. (2000, Thm. 2.1) yields the claim.

□

S2.2 Correlated errors: sparse-precision extension

Theorem S2.4 (Posterior contraction under correlated errors)**.** *Assume* (A1)*,* (A2)*,* (A4)*, and* (A5)(i)*. Equip the residual precision $\Omega = \Sigma^{-1}$ with a sparse-precision prior as in* (A5)(iii) *that yields contraction at $\varepsilon_\Omega \asymp \sqrt{s_\Omega \log Q/N}$ in Frobenius norm. Then, for any fixed $M > 0$ and some constant $C > 0$,*

$$\Pi(\,\|B - B_0\|_F \;>\; M\,\delta_N \,|\, X, Y) \to 0, \qquad \Pi(\,\|\Omega - \Omega_0\|_F \;>\; C\,\varepsilon_\Omega \,|\, X, Y) \to 0,$$

in P_{B_0,Σ_0}-probability as $N \to \infty$. In particular, the contraction rate for B matches the diagonal-Σ_0 case up to constants.

Proof. By (A5)(iii) and results for sparse precision priors, the marginal posterior for Ω contracts at rate ε_Ω in Frobenius norm; see Banerjee & Ghosal (2014). Hence

$$\Pi\big(\|\Omega - \Omega_0\|_F \le \varepsilon_\Omega \,\big|\, X, Y\big) \;\to\; 1.$$

On $\{\|\Omega - \Omega_0\|_{\rm op} \le c\varepsilon_\Omega\}$ and by continuity of $A \mapsto A^{1/2}$ on $\mathbb{S}^Q_{++}$, $\|\Omega^{1/2} - \Omega_0^{1/2}\|_{\rm op} \lesssim \varepsilon_\Omega$. Consider

$$Y^\star \;=\; Y\Omega^{1/2} \;=\; X\,(B\Omega^{1/2}) \;+\; E\Omega^{1/2},$$

which has identity covariance when $\Omega = \Omega_0$. Conditional on Ω in an $O(\varepsilon_\Omega)$-ball of Ω_0, the testing/entropy argument for $B^\star := B\Omega^{1/2}$ gives rate δ_N. Finally,

$$\|B - B_0\|_F \;\le\; \|B\Omega^{1/2} - B_0\Omega_0^{1/2}\|_F \cdot \|\Omega^{-1/2}\|_{\rm op} \;+\; \|B_0(\Omega_0^{1/2} - \Omega^{1/2})\|_F \cdot \|\Omega^{-1/2}\|_{\rm op},$$

and spectral bounds in (A4) yield the claim since $\varepsilon_\Omega = o(1)$ while δ_N is the dominating rate under (A2). □

S2.3 Misspecification robustness

Theorem S2.5 (Posterior contraction under misspecification)**.** *Assume* (A1) *and* (A2), *and that the data satisfy $y_i = x_i B_0 + \varepsilon_i$ with ε_i i.i.d. mean-zero and sub-Gaussian with covariance Σ_0 (not necessarily diagonal or correctly modeled). Consider the working Gaussian family $\{p_{(B,\Sigma)}\}$ with* diagonal $\Sigma = \mathrm{diag}(\sigma_1^2, \dots, \sigma_Q^2)$, *and define the pseudo-true (KL) projection*

$$(B^\star, \Sigma^\star) \in \arg \min_{B,\, \Sigma \in \mathbb{S}^Q_{++} \cap \mathrm{diag}} \mathrm{KL}\big(P_0 \,\|\, p_{(B,\Sigma)}\big).$$

Assume (A5)(i) *for the coefficient prior, and* either *of the following specifications for Σ:*

(S1) Fixed working covariance. Σ is fixed a priori (e.g., a given diagonal matrix). Then only (A5)(i) *is used.*

(S2) Unknown diagonal covariance. Equip $\Sigma = \mathrm{diag}(\sigma_1^2, \dots, \sigma_Q^2)$ with any proper *heavy-tailed scale prior on each σ_q having a continuous, strictly positive density in a neigh-*

borhood of $\sigma_q^\star$ (e.g., half-t_ν or scaled-inverse-χ^2), as allowed in (A5)(ii)*. This ensures the prior-mass condition in neighborhoods of $\Sigma^\star$ required by Kleijn & van der Vaart (2006, Thm. 2.1).*

Then, for any fixed $M > 0$,

$$\Pi\left(\|B - B^\star\|_F > M\sqrt{\frac{s\log(PQ)}{N}} \,\middle|\, X, Y \right) \to 0 \quad \text{in } P_0\text{-probability as } N \to \infty.$$

In the present linear-mean setting we have $B^\star = B_0$ (hence the posterior contracts around B_0).

Proof. **Step 1: Identify $B^\star$.** In the linear-mean model $E_{P_0}(y_i \mid X) = x_i B_0$. For any diagonal $\Sigma \in \mathbb{S}_{++}^Q$,

$$\mathbb{E}_{P_0}\big[(y_i - x_iB)^\top \Sigma^{-1}(y_i - x_iB)\big] = (x_i(B - B_0))^\top \Sigma^{-1}(x_i(B - B_0)) + \operatorname{tr}(\Sigma^{-1}\Sigma_0),$$

so the population risk is minimized at $B = B_0$ regardless of Σ. Hence $B^\star = B_0$.

Step 2: Prior thickness and KL neighborhoods. Under (A5)(i), the coefficient prior density is continuous and strictly positive in neighborhoods of $B^\star$, and has exponentially decaying tails (for sieve control), cf. Castillo et al. (2015). In case (S1), Σ is fixed, so the parameter is B only. In case (S2), the prior on Σ is *proper* with positive continuous density near $\Sigma^\star$, guaranteeing positive prior mass on every KL neighborhood of $(B^\star, \Sigma^\star)$ as required by Kleijn & van der Vaart (2006, Thm. 2.1). (We do not invoke diagonal truth (A3) here.)

Step 3: Tests. With sub-Gaussian errors and design condition (A1), the likelihood-ratio statistic for testing $H_0 : B = B^\star$ versus $H_1 : \|B - B^\star\|_F > M\delta_N$ (computed under the working Gaussian model) yields exponentially powerful tests: its mean separates by a constant multiple of $N\|B - B^\star\|_F^2$ and its fluctuations are sub-exponential, so the type I and II errors are bounded by $\exp\{-cN\delta_N^2\}$ uniformly over H_1, exactly as in the diagonal case (see the construction in Theorem S2.3 and van der Vaart 1998, Ch. 10 for the general LAN/BvM context).

Step 4: Sieve and entropy. Use the same sieve $\mathcal{F}_N = \{B : \|B\|_\infty \le M_N,\ |\text{supp}(B)| \le s\}$ with $M_N \asymp s\log(PQ)$; by the exponential tails in (A5)(i), $\Pi(\mathcal{F}_N^c) \le \exp\{-C\, s\log(PQ)\} =$

$\exp\{-C\,N\delta_N^2\}$, and

$$\log N(\epsilon, \mathcal{F}_N, \|\cdot\|_F) \;\lesssim\; s\log(PQ) + s\log(1/\epsilon),$$

so with $\epsilon = \delta_N$ we have $\log N(\delta_N, \mathcal{F}_N, \|\cdot\|_F) \le C' N\delta_N^2$, cf. Ghosal et al. (2000).

Step 5: Apply misspecified contraction theorem. The ingredients of Kleijn & van der Vaart (2006, Thm. 2.1)—prior mass in KL neighborhoods of $(B^\star, \Sigma^\star)$, existence of exponentially powerful tests, and suitable entropy bounds—are thus verified (in S1 with respect to B only; in S2 jointly in (B, Σ)). It follows that the posterior for B contracts at rate $\delta_N = \sqrt{s\log(PQ)/N}$ around $B^\star$ in P_0-probability. □

Remark S2.6 (Which (A5) parts are used). The theorem always uses (A5)(i) (coefficient prior). If Σ is treated as fixed (S1), neither (A5)(ii) nor (A5)(iii) is used. If Σ is assigned a prior (S2), the result uses (A5)(ii) *with a proper heavy-tailed scale prior* to ensure prior mass near $\Sigma^\star$; (A5)(iii) (sparse precision prior) is *not* used in this theorem.

Remark S2.7 (On credible intervals under misspecification). Under misspecification, credible sets for individual entries target $B^\star$ and are not guaranteed to provide frequentist coverage for B_0 (Kleijn & van der Vaart 2006). In the present linear-mean setting $B^\star = B_0$, so credible sets still target the true mean coefficients; however, coverage can still be affected by variance misspecification. Our row-exceedance selector (Theorem S2.8) relies on posterior events for $\|\beta_{p\cdot}\|_2$ and is valid under either specification or misspecification.

S2.4 Row-support (master-predictor) recovery

Define the set of master predictors $S_{\text{row}} := \{p \in \{1, \ldots, P\} : \|\beta_{0,p\cdot}\|_2 > 0\}$ and let $s_{\text{row}} := |S_{\text{row}}|$. For a sequence $\tau_N > 0$, consider the posterior row-exceedance selector

$$\widehat{S} \;:=\; \Big\{p:\; \Pi\big(\,\|\beta_{p\cdot}\|_2 > \tau_N \,\big|\, X, Y\big) \;>\; 1 - \alpha_N\Big\}$$

for some $\alpha_N \downarrow 0$.

Theorem S2.8 (Sure screening and selection consistency)**.** *Under the conditions of Theorem S2.3 or Theorem S2.4, let* $\tau_N := C\sqrt{\log(PQ)/N}$ *and* $\alpha_N := e^{-cN\tau_N^2}$ *for fixed* $C, c > 0$. *Then:*

(i) (Sure screening) $S_{\text{row}} \subseteq \widehat{S}$ *with probability* $\to 1$.

(ii) (Exact selection under beta-min) If in addition

$$\beta_{\min} := \min_{p \in S_{\text{row}}} \|\beta_{0,p\cdot}\|_2 \ \geq \ K\sqrt{\log(PQ)/N}$$

for sufficiently large K*, then* $\widehat{S} = S_{\text{row}}$ *with probability* $\to 1$.

Proof. By Theorem S2.3 (or S2.4), the posterior concentrates in an $O(\delta_N)$-ball around B_0 in Frobenius norm. Hence for $p \in S_{\text{row}}$,

$$\Pi\big(\|\beta_{p\cdot}\|_2 \leq \tau_N \mid X, Y\big) \ \leq \ \Pi\big(\|\beta_{p\cdot} - \beta_{0,p\cdot}\|_2 \geq \beta_{\min} - \tau_N \mid X, Y\big) \ \to \ 0$$

provided $\beta_{\min} \gg \tau_N$. Thus $\Pi(\|\beta_{p\cdot}\|_2 > \tau_N \mid X, Y) \to 1$, so $p \in \widehat{S}$ for all $p \in S_{\text{row}}$ with probability $\to 1$. For $p \notin S_{\text{row}}$ we have $\|\beta_{0,p\cdot}\|_2 = 0$ and thus, for any B,

$$\|\beta_{p\cdot}\|_2 \ \leq \ \|B - B_0\|_F.$$

Hence,

$$\Pi\big(\, \|\beta_{p\cdot}\|_2 > \tau_N \,\big|\, X, Y\big) \ \leq \ \Pi\big(\, \|B - B_0\|_F > \tau_N \,\big|\, X, Y\big).$$

By the same test–entropy argument used in Theorem S2.3 with $\epsilon = \tau_N$ (cf. Ghosal et al. 2000, Thm. 2.1), there exist constants $c, c' > 0$ such that

$$\mathbb{E}_0\Big[\Pi\big(\, \|B - B_0\|_F > \tau_N \,\big|\, X, Y\big)\Big] \ \leq \ e^{-cN\tau_N^2} \quad \Rightarrow \quad \Pi\big(\, \|B - B_0\|_F > \tau_N \,\big|\, X, Y\big) \ \leq \ e^{-c'N\tau_N^2}$$

in P_{B_0,Σ_0}-probability. Taking $\alpha_N := e^{-c'N\tau_N^2}$ and using $N\tau_N^2 = C^2 \log(PQ)$, a union bound over $p \notin S_{\text{row}}$ shows that, for C large enough, no noise row is selected with probability $\to 1$. Together with the sure-screening part and the beta-min condition, this proves (i)–(ii).

□

S3 Simulation Studies

We consider two simulation studies. In the first study, we compare the performance of the proposed B-MASTER method with a few existing methods using a synthetic dataset

generated mimicking the real dataset. This simulation study allows us to benchmark B-MASTER against other existing methods while keeping X unchanged, thereby retaining the original covariance structure of the dataset. This setup enables us to investigate relative performances in a more practical and relevant context, aligned with our ultimate goal of analyzing the real dataset. In the second study, we focus primarily on evaluating the computational performance of B-MASTER in a different set up as the dimensions of X and Y increase.

S3.1 Real-data Based Simulation Study

The real dataset (Yachida et al. 2019), described and analyzed later in this paper, consists of $P = 287$ microbiome features and $Q = 249$ metabolite features from $N = 220$ subjects. Using B-MASTER, the optimal B' is first estimated and then considered the true coefficient matrix for the simulation study. Then, we generate

$$Y_q^i = X^i B_q' + \epsilon_{iq}$$

for $i = 1, \ldots, N$, where $B_q' = (\beta_{pq}')_{p=1}^P$ denotes the q-th column of B' and $\epsilon_{iq} \sim N(0, \sigma^2)$; we take $\sigma = 1$. Ten sets of synthetic Y are generated, followed by the performance evaluation of B-MASTER and other methods using the actual X (as in the original dataset) and B'.

B-MASTER is implemented in MATLAB. The values of the model parameters provided in Section S1.5. B-MASTER is run for 1,000 iterations discarding the first 100 posterior samples as burn-in. It is observed that the results obtained using B-MASTER under the aforementioned setup are not noticeably different from those obtained after 100 iterations, discarding the first 10 iterations as burn-in. However, for the comparative study, the analysis results are reported under the first setup (i.e., 1,000 iterations with 100 burn-in).

To identify the non-significant coefficients, we construct a two-sided symmetric 90% posterior credible interval for each β_{pq} based on the posterior samples. Coefficients β_{pq}s whose posterior credible intervals contain 0 are discarded as non-significant. To calculate the area under the Receiver Operating Characteristic (ROC) curve (AUC), Bayesian p-values corresponding to each β_{pq} are computed as follows. Suppose $\{\beta_{pq}^{(1)}, \ldots, \beta_{pq}^{(M)}\}$ denote the posterior sample values of coefficient β_{pq}. Let $\hat{p}^+$ denote the proportion of β_{pq} samples

which are > 0; and let $\hat{p}^-$ denote the proportion of β_{pq} samples which are ≤ 0. Then

$$\text{Bayesian p-value} = 2 * \min(\hat{p}^+, \hat{p}^-).$$

We take an equidistant grid of cut-off points of length 501 over the interval $[0, 0.5]$. For each cut-off point, we calculate the true positive rate (TPR) and false positive rate (FPR) based on Bayesian p-values. Additionally, we compute AUC20 (i.e., AUC20 = 5×Area under the ROC curve up to 20% FPR) and Mathew's Correlation Coefficient (MCC) (Chicco & Jurman 2020), which is given by

$$\text{MCC} = \frac{TP \cdot TN - FP \cdot FN}{\sqrt{(TP + FP)(TP + FN)(TN + FP)(TN + FN)}},$$

where TP, TN, FP, FN denote true positives, true negatives, false positives and false negatives, respectively.

For the comparative study, we consider a few existing methods, namely Regularized Multivariate regression for identifying master predictors (Peng et al. 2010), univariate and multivariate Spike-and-Slab LASSO (Rockova & George 2018, Deshpande et al. 2019); hereafter referred to as remMap, SSLASSO and mSSL, respectively. SSLASSO and mSSL employ spike-and-slab LASSO in the frequentist framework, which, unlike remMap or B-MASTER, does not impose an additional L2 penalty to discourage non-essential regressors. RemMap is implemented using the R package `remMap` (Peng et al. 2010). It should be noted that the `remMap` package provides two ways to estimate the coefficient matrix. The first method involves using the function `remMap.CV` to perform cross-validation over a predefined grid of tuning parameter values. This is followed by fitting the model using the function `remMap` with the optimal tuning parameters determined in the first step. The alternative function, `remMap.BIC` selects the best model using the Bayesian Information Criterion (BIC, Schwarz (1978)) over a grid of given values for the tuning parameters. The authors noted that the second method is computationally more feasible. They also pointed out that this alternative BIC-based option tends to select models that are too small when the actual design matrix is far from orthogonal. For both `remMap` and `remMap.BIC`, we use a grid of λ_1 values that are exponential of an equidistant grid of length 11, spanning over the range from 0 and $log(1000)$. λ_2 values are taken to be an equidistant grid of length 11, spanning over the range from 0 and 1000. We opt for 5-fold cross-validation. SSLASSO and mSSL are

Methods	TPR	FPR	MCC	AUC	AUC20	Sparsity (True = 0.76)
B-MASTER	0.84 (0.0012)	0.01 (0.0003)	0.87 (0.0001)	0.98 (0.0004)	0.94 (0.0014)	0.79 (0.0003)
SSLASSO	0.23 (0.0008)	0.01 (0.0004)	0.41 (0.0013)	0.89 (0.0004)	0.54 (0.0007)	0.94 (0.0002)
mSSL-dcpe	0.14 (0.0018)	0.00 (0.0001)	0.31 (0.0024)	0.76 (0.0006)	0.44 (0.0013)	0.96 (0.0005)
remMap-bic	0.00 (0.0001)	0.00 (0.0000)	0.02 (0.0008)	0.58 (0.0015)	0.23 (0.0009)	1.00 (0.0000)

Table S2: A comparative study of B-MASTER, SSLASSO, mSSL, and remMap based on the true positive rate (TPR), false positive rate (FPR), Matthew's Correlation Coefficient (MCC), Area under the ROC curve (AUC), and AUC20 from 10 simulation experiments is presented. Mean measure values are provided, with standard errors in parentheses.

implemented using R packages `SSLASSO` (Rockova & George 2018) and `mSSL` (Deshpande et al. 2019), respectively. SSLASSO is fit using the `SSLASSO` function within the package of the same name. The R package `mSSL` offers two model-fitting options, namely `mSSL_dpe` and `mSSL_dcpe`, respectively. `mSSL_dpe` performs the Expectation-Conditional Maximization (ECM) algorithm, which targets the MAP estimates of the coefficient matrix. `mSSL_dcpe` is proposed as a faster approximate alternative of `mSSL_dpe`. We opt for the default option to select the grid of tuning parameter values. Note that, for all the above-mentioned methods, the results corresponding to the respective grids are later used to calculate AUC and AUC20.

All simulation experiments are performed on a desktop running the Windows 10 Enterprise operating system desktop with 32 GB RAM and the following processor characteristics: 12th Gen Intel(R) Core(TM) i7-12700, 2100 Mhz, 12 Cores(s), 20 Logical Processor(s). We observe that `mSSL_dpe` and `remMap.CV` did not terminate or produce results within 48 hours, possibly due to the high number of parameters considered (P*Q $= 249 \times 287 = 71463$) in the simulation settings. We report only the results corresponding to the methods mSSL and remMap, using the functions `mSSL_dcpe` (from the R package `mSSL`) and `remMap.BIC` (from the R package `remMap`). The calculated TPR, FPR, MCC, AUC, AUC20, and sparsity proportion of the estimated coefficient matrices under different methods are provided in Table S2. The standard errors for all considered measures are noted in parentheses. We observe that B-MASTER outperforms SSLASSO, mSSL, and RemMap by a large margin. It is noted that, except for B-MASTER, the other methods overly shrink the coefficients, resulting in a very low TPR. In terms of AUC, SSLASSO performed the best among the other methods. We would like to point out to the readers that, since the original functions for mSSL and RemMap did not converge within 48 hours, we were only able to use their approximate, faster alternative versions, which are susceptible to the over-shrinking issue,

as mentioned by the corresponding authors.

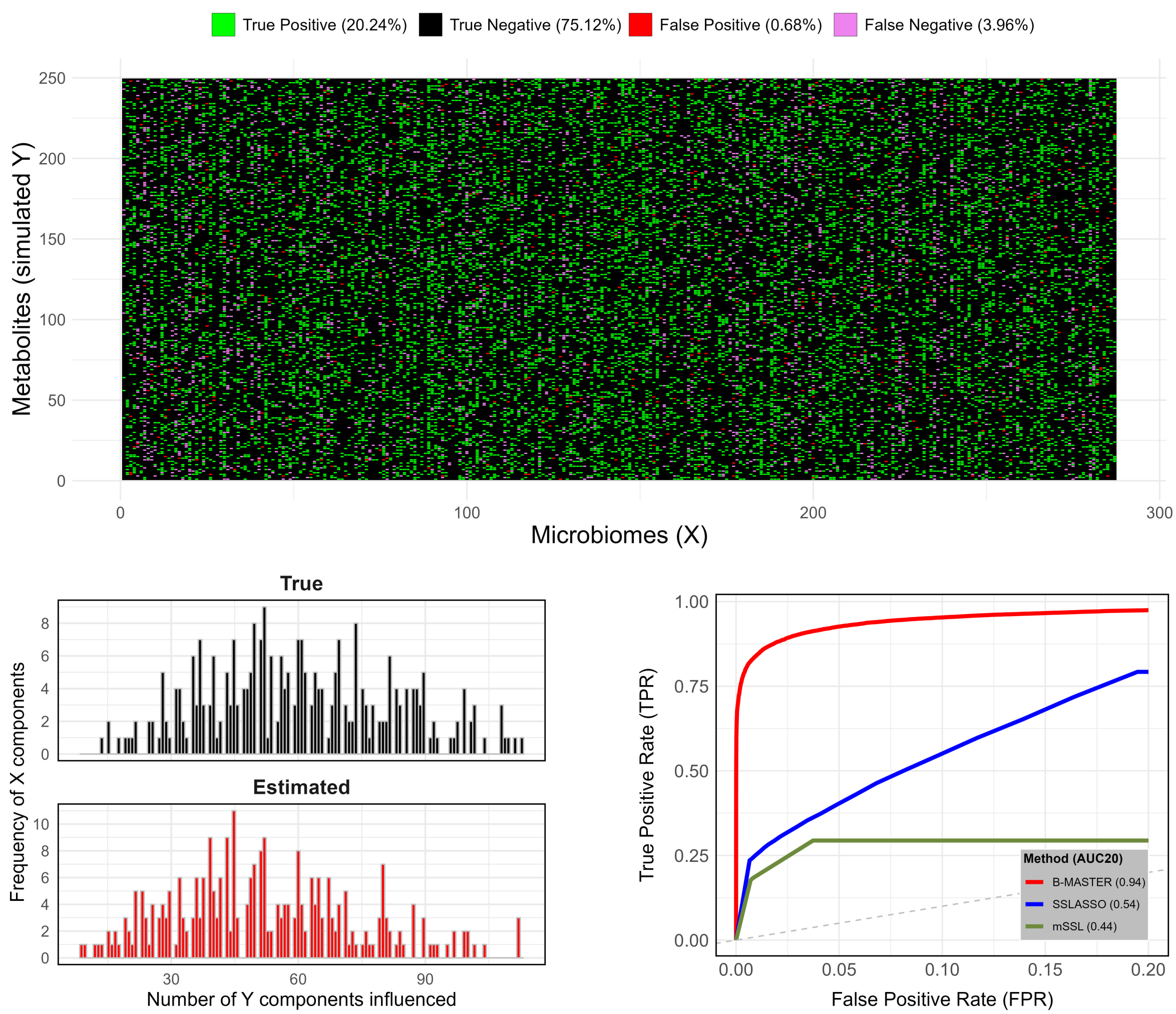

Figure S1: *Up:* Signal detection performance of B-MASTER is evaluated based on Simulation Study 1. True and B-MASTER estimated coefficient signals are depicted, divided into categories: true positives (TP), true negatives (TN), false positives (FP), and false negatives (FN). *Bottom-left:* True and B-MASTER estimated numbers of Y components influenced by X components are depicted. *Bottom-right:* The AUC curve is plotted up to a false positive rate of 20% for the B-MASTER, SSLASSO, and mSSL methods. AUC20 values are provided in parentheses within the legend.

In Figure S1 (*up*), the true and B-MASTER estimated coefficient signals are depicted, divided into four categories: TP, TN, FP, and FN. The number of Y components influenced by X components under both true and B-MASTER estimated scenarios is shown as a histogram in Figure S1 (*bottom-left*). It appears that B-MASTER is slightly conservative in selecting variables, which creates a slight leftward shift in the histograms under the B-MASTER estimated scenario. Figure S1 (*bottom-right*) shows that the AUC curve of

B-MASTER dominates over SSLASSO and mSSL across the FPR range from 0% to 20%, by a large margin. We chose to display the AUC curve up to the 20% FPR zone because the grid points of FPR and TPR obtained from the SSLASSO and mSSL model fittings (with grids taken as default options) were very sparse for FPR values greater than 20%. We refrain from plotting the AUC curve for remMap, as Table S2 already indicates its under-performance in the considered experiment.

S3.2 Evaluation of B-MASTER in Higher Dimensions

To evaluate the performance and required computation times in higher-dimensional settings for B-MASTER, we conduct another simulation study. We generate N realizations of X from $MVN(\mathbf{0}, \Sigma)$, where $\Sigma = \big(\rho_{i,j}\big)_{P \times P}$ with $\rho_{i,j} = \rho^{|i-j|}$. We then generate $\boldsymbol{B}_0$ (a matrix of dimension $P \times Q$), where each element of $\boldsymbol{B}_0$ is sampled from $U\big([-5, -1] \cup [1, 5]\big)$. Hereafter, we generate an indicator matrix to impose sparse structure over the coefficient matrix adhering to the following presumptions:

- 20% of the X components influence all Y components.
- Another 20% of the X components influence $100s\%$ of the Y components selected at random, where $s \sim U(.4, .6)$.
- The remaining 60% X components are assumed to have no effect on Y.

We construct an indicator matrix I_0 of dimension $P \times Q$, where 1s and 0s are assigned based on the aforementioned assumptions. Finally, we compute $\boldsymbol{B} = \boldsymbol{B}_0 \circ I_0$, where $\circ$ denotes element-wise product. Then, we generate

$$Y_q^i = X^i B_q + \epsilon_{iq}$$

for $i = 1, \ldots, N$, where $B_q = (\beta_{pq})_{p=1}^{P}$ denotes the q-th column of $\boldsymbol{B}$ and $\epsilon_{iq} \sim N(0, \sigma^2)$; we take $\sigma = 0.1$. For all considered scenarios, we set $P = Q = N$ inspired by the fact that in the real-dataset, the values of P, Q, N are similar ($P = 287, Q = 249, N = 220$). The performance of B-MASTER is evaluated for $P = 20, 50, 100, 200, 500, 1000, 2000$. All these sub-scenarios considered taking ρ to be 0 (independent components) and 0.5 (highly correlated components), separately. The simulation results are provided in Table S3. For each case, along with TPR, FPR, MCC, AUC, AUC20, estimated sparsity, and true sparsity (in parentheses), we also report the computation times. Although the results are based on 100

iterations with 20 burn-in, the projected time to complete 1000 iterations is also provided. Since each iteration takes a similar amount of time for a given dimensional setting, the projected time to complete 1000 iterations is approximately 10 times the time spent performing 100 iterations. Note that, for the experiments considered, we did not observe any further noticeable improvement when using 1000 iterations instead of 100. It was observed that for both cases, across all considered dimensions, B-MASTER performed well, yielding near-perfect TPR, FPR, AUC, MCC, and AUC20. The estimated sparsity was also very close to the true sparsity, showing a slight trend toward being conservative, which resulted in the estimated $\boldsymbol{B}$ being marginally sparser than the true model. The B-MASTER results for the independent X scenario are slightly better than those for the correlated case. Furthermore, as the dimension of the parameter space increases, the computation time increases linearly, making it highly scalable for higher-dimensional models. This scalability enables us to evaluate B-MASTER performance in a model with up to 4 million coefficients within a reasonable amount of time. In Figure S2, we demonstrate the log-log plot (with base 10) of the number of parameters vs. computation time (in minutes) for B-MASTER in the $\rho = 0.5$ scenario, revealing a linear dependence relationship. The approximate value of the slope is noted to be 1.06. The plot for the $\rho = 0$ scenario is nearly identical, and therefore, we refrain from plotting it.

To further contextualize the higher-dimensional simulation results, we additionally compared B-MASTER with SSLASSO and remMap-bic for the lower-dimensional sub-scenarios $P = Q = N \in \{20, 50, 100, 200\}$; these results are summarized in Table S4. Across both the independent and correlated predictor settings, B-MASTER consistently achieved substantially lower FPR and higher MCC than the competing methods, while maintaining comparable or superior AUC and AUC20 values. Although SSLASSO and remMap-bic often attained high TPR, this came at the cost of noticeably inflated FPR and less accurate sparsity recovery, suggesting reduced precision in support identification. We also evaluated mSSL-dcpe, but it repeatedly produced degenerate, non-informative solutions in these settings, typically corresponding to nearly all-zero coefficient estimates; hence, we omitted it from the reported comparison.

ρ	P, Q, N	TPR	FPR	MCC	AUC	AUC20	Sparsity (true sparsity)	Number of parameters	Number of parameter folds	Comp. time for 100 iters. (in mins)	Comp.time folds	Comp. time for 1000 iters. (projected, in mins)
$\rho = 0$	20	0.974	0.000	0.982	0.999	0.999	0.715 (0.708)	400	1x	0.04	1x	0.37
	50	0.991	0.000	0.993	1.000	1.000	0.708 (0.705)	2500	6.25x	0.19	5.24x	1.94
	100	0.978	0.012	0.964	0.997	0.986	0.699 (0.700)	10000	25x	0.74	19.97x	7.39
	200	0.972	0.026	0.937	0.995	0.980	0.692 (0.702)	40000	100x	4.68	126.38x	46.76
	500	0.996	0.029	0.951	0.998	0.990	0.681 (0.699)	250000	625x	26.68	721.03x	266.78
	1000	0.998	0.006	0.989	0.999	0.999	0.697 (0.700)	1000000	2500x	108.34	2928.19x	1083.43
	2000	0.998	0.000	0.998	1.000	1.000	0.702 (0.701)	4000000	10000x	603.65	16314.87x	6036.50
$\rho = 0.5$	20	0.966	0.000	0.976	1.000	1.000	0.718 (0.708)	400	1x	0.04	1x	0.36
	50	0.999	0.002	0.996	1.000	1.000	0.704 (0.705)	2500	6.25x	0.19	5.14x	1.85
	100	0.982	0.025	0.947	0.994	0.970	0.689 (0.700)	10000	25x	0.71	19.64x	7.07
	200	0.963	0.033	0.921	0.992	0.969	0.690 (0.702)	40000	100x	4.78	132.72x	47.78
	500	0.990	0.021	0.959	0.998	0.991	0.688 (0.699)	250000	625x	27.84	773.39x	278.42
	1000	0.984	0.001	0.988	1.000	1.000	0.705 (0.700)	1000000	2500x	119.65	3323.53x	1196.47
	2000	0.947	0.000	0.963	1.000	0.999	0.717 (0.701)	4000000	10000x	644.97	17915.75x	6449.67

Table S3: Performance evaluation of B-MASTER: The values of P ($P = Q = N$), TPR, FPR, MCC, AUC, AUC20, sparsity (with true sparsity in parentheses), the number of parameters, relative folds of the number of parameters, computation time for 100 iterations (in minutes), relative folds of computation times, and the projected computation time for 1000 iterations (in minutes) are provided taking $P = Q = N = 20, 50, 100, 200, 500, 1000, 2000$ under scenarios $\rho = 0, 0.5$.

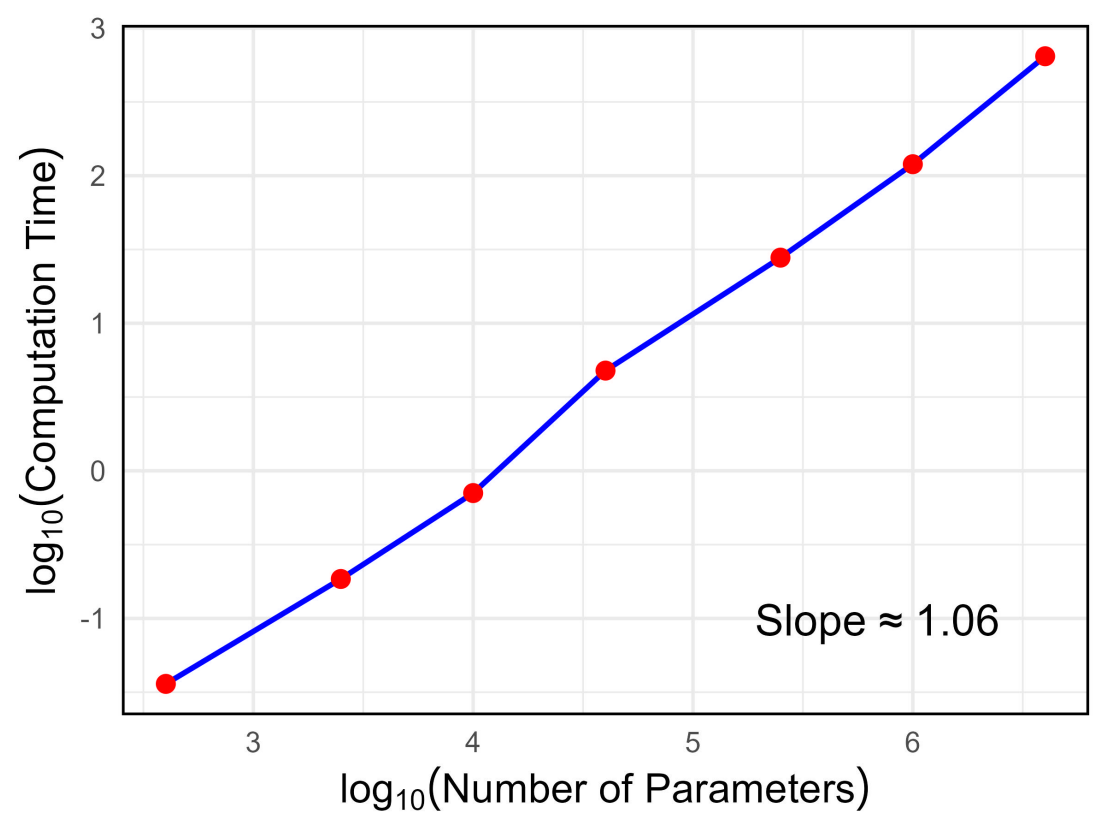

Figure S2: The plot shows log_{10}(Number of Parameters) versus log_{10}(Computation Time) for B-MASTER applied across scenarios with $P = Q = N = (20, 50, 100, 200, 500, 1000, 2000)$ and $\rho = 0.5$. Computation time is measured in minutes.

ρ	P (P=Q=N)	Method	TPR	FPR	MCC	AUC	AUC20	Sparsity (true sparsity)
$\rho = 0$	20	B-MASTER	0.974	0.000	0.982	1.000	0.999	0.715 (0.708)
		SSLASSO	0.983	0.124	0.807	0.959	0.826	0.625 (0.708)
		remMap-bic	0.983	0.124	0.807	0.956	0.810	0.625 (0.708)
	50	B-MASTER	0.991	0.000	0.993	1.000	1.000	0.708 (0.705)
		SSLASSO	1.000	0.109	0.841	0.959	0.819	0.628 (0.705)
		remMap-bic	1.000	0.109	0.841	0.961	0.804	0.628 (0.705)
	100	B-MASTER	0.978	0.012	0.964	0.997	0.986	0.699 (0.700)
		SSLASSO	1.000	0.131	0.815	0.961	0.804	0.609 (0.700)
		remMap-bic	1.000	0.131	0.815	0.965	0.826	0.608 (0.700)
	200	B-MASTER	0.972	0.026	0.937	0.995	0.980	0.692 (0.702)
		SSLASSO	1.000	0.143	0.800	0.959	0.793	0.601 (0.702)
		remMap-bic	1.000	0.143	0.800	0.975	0.876	0.601 (0.702)
$\rho = 0.5$	20	B-MASTER	0.966	0.000	0.976	1.000	1.000	0.718 (0.708)
		SSLASSO	0.983	0.148	0.777	0.941	0.740	0.608 (0.708)
		remMap-bic	0.983	0.148	0.777	0.936	0.711	0.608 (0.708)
	50	B-MASTER	0.999	0.002	0.996	1.000	1.000	0.704 (0.705)
		SSLASSO	0.988	0.154	0.777	0.931	0.678	0.600 (0.705)
		remMap-bic	0.988	0.154	0.776	0.925	0.650	0.600 (0.705)
	100	B-MASTER	0.982	0.025	0.947	0.994	0.970	0.689 (0.700)
		SSLASSO	0.996	0.198	0.737	0.929	0.653	0.563 (0.700)
		remMap-bic	0.996	0.198	0.737	0.945	0.733	0.563 (0.700)
	200	B-MASTER	0.963	0.033	0.921	0.992	0.969	0.690 (0.702)
		SSLASSO	1.000	0.195	0.742	0.931	0.654	0.565 (0.702)
		remMap-bic	1.000	0.195	0.742	0.961	0.805	0.565 (0.702)

Table S4: Comparative study of B-MASTER, SSLASSO, and remMap-bic in the second simulation study based on true positive rate (TPR), false positive rate (FPR), Matthews Correlation Coefficient (MCC), AUC, AUC20, and sparsity recovery. Results are reported for $P = Q = N \in \{20, 50, 100, 200\}$ under independent ($\rho = 0$) and correlated ($\rho = 0.5$) predictor scenarios. Estimated sparsity values are reported with the corresponding true sparsity shown in parentheses.

S4 Convergence Diagnostics and Hyperparameter Sensitivity

S4.1 B-MASTER Convergence Diagnostics

To evaluate MCMC convergence, we monitored Monte Carlo error and stationarity diagnostics for the two global shrinkage parameters (λ_1^2, λ_2^2) and five representative B coefficients corresponding to the largest absolute entries of B. Figure S3 displays trace plots with running mean overlays for these seven quantities. For each series, we report the ratio of Monte Carlo standard error to posterior standard deviation (MCSE/SD%) as a measure of numerical accuracy and Geweke's diagnostic z-statistic, which compares the first 10% of the chain to the last 50%. Table S5 summarizes these diagnostics. Across λ_2^2 and the B entries, MCSE/SD ratios were consistently below 5% and all Geweke statistics lay within $|z| \leq 2$, supporting satisfactory mixing and stationarity. The behavior of λ_1^2 was distinct: this parameter rapidly decayed to values near machine precision, yielding an MCSE/SD ratio slightly above 5% but with a Geweke statistic of essentially zero. This reflects strong shrinkage toward zero rather than poor mixing, and is consistent with the posterior concentrating on negligible contribution from λ_1^2. This convergence analysis was conducted for the real data–based simulation study considered in Section 4.1 (main draft), using 1000 total iterations and discarding the first 100 iterations as burn-in.

Quantity	MCSE/SD (%)	Geweke z
λ_1^2	8.77	< 0.01
λ_2^2	0.65	-0.57
$B[1]$	3.30	-0.04
$B[2]$	3.17	-1.22
$B[3]$	3.66	0.67
$B[4]$	2.68	0.56
$B[5]$	3.24	-0.42

Table S5: MCMC diagnostics (MCSE/SD% and Geweke z) for λ_1^2, λ_2^2, and five representative B entries (corresponding to the top five absolute values of B).

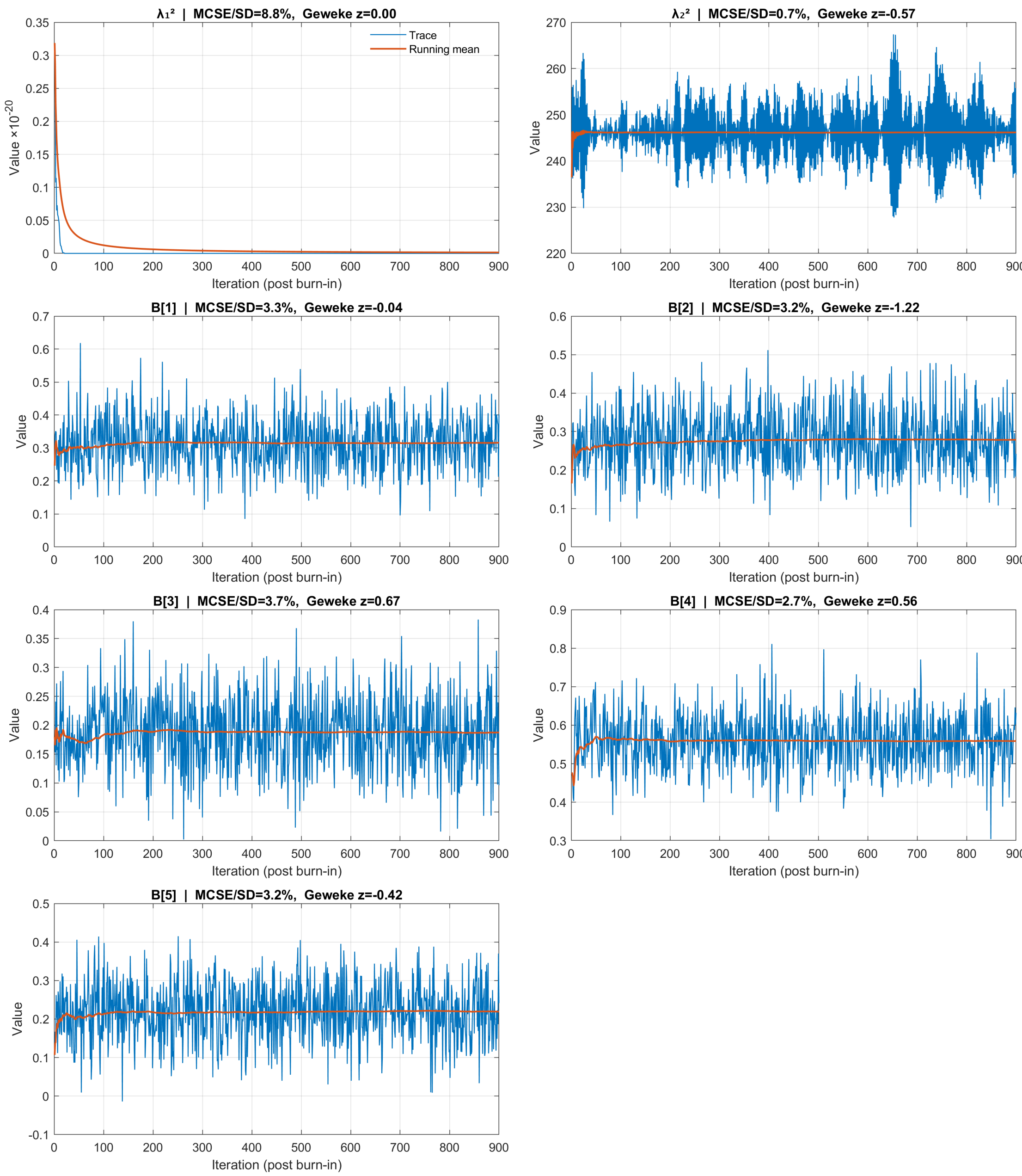

Figure S3: Trace plots and running means for λ_1^2, λ_2^2, and five representative B entries ($B[1]$–$B[5]$), chosen as the five largest absolute values of B. For each parameter, we report the Monte Carlo standard error relative to posterior variability (MCSE/SD%) and Geweke's stationarity z-score. Low MCSE/SD values ($< 10\%$) and Geweke statistics within $|z| \leq 2$ provide evidence of satisfactory convergence.

S4.2 Sensitivity Analysis for Hyper–Priors

The hyper–prior specification for the global shrinkage parameters λ_1^2 and λ_2^2 involves four constants $r_1,\ r_2,\ \delta_1,\ \delta_2$. In our implementation, we set $r_1 = r_2 = 1$ and examine sensitivity primarily with respect to (δ_1, δ_2), which directly control the effective shrinkage strength through the Gamma rate parameters. Specifically, the conditional posterior distributions are given by

$$\lambda_1^2 \mid \boldsymbol{T}^2 \sim \text{Gamma}\left(PQ + r_1,\ \frac{1}{2}\sum_{p=1}^{P}\sum_{q=1}^{Q}\tau_{pq}^2 + \delta_1\right),$$

and

$$\lambda_2^2 \mid \boldsymbol{G}^2 \sim \text{Gamma}\left(\frac{PQ}{2} + r_2,\ \frac{1}{2}\sum_{p=1}^{P}\gamma_p^2 + \delta_2\right).$$

Because the shape parameters scale with the total coefficient dimension PQ, the additive constants $r_1 = r_2 = 1$ contribute negligibly in the high–dimensional settings considered in this paper. Consequently, the posterior behavior is driven primarily by the latent shrinkage quantities $\{\tau_{pq}^2\}$ and $\{\gamma_p^2\}$ together with the rate parameters (δ_1, δ_2). Empirically, varying r_1 and r_2 over small positive values produced nearly identical posterior summaries and selection results. Therefore, throughout the manuscript we fix $r_1 = r_2 = 1$ and focus sensitivity analyses on (δ_1, δ_2).

In contrast, the Gamma rates,

$$b_1 = \tfrac{1}{2}\sum_{p,q}\tau_{pq}^2 + \delta_1, \qquad b_2 = \tfrac{1}{2}\sum_{p=1}^{P}\gamma_p^2 + \delta_2,$$

directly control the global shrinkage through λ_1^2 and λ_2^2. We therefore assess sensitivity to (δ_1, δ_2) using the real data-based simulation of Section 4.1 (main draft): for each setting we run 1,000 iterations, discard the first 100 as burn–in, and summarize performance over 10 replicates using TPR, FPR, MCC, AUC, AUC20, and sparsity. We consider the default $(\delta_1, \delta_2) = (0.1, 0.1)$ together with four alternatives $(0.01, 0.01)$, $(0.01, 1)$, $(1, 0.01)$, and $(1, 1)$. The results are reported in Table S6, in the same format as Table 1 of the main text (means with standard errors in parentheses). Across all (δ_1, δ_2) pairs, TPR, FPR, MCC, AUC, AUC20, and sparsity remain essentially unchanged within Monte Carlo uncertainty, with the default $(0.1, 0.1)$ performing on par with the alternatives. This indicates that posterior inference and variable–selection behavior of B-MASTER are robust

(δ_1, δ_2)	TPR	FPR	MCC	AUC	AUC20	Sparsity (True = 0.76)
Default (0.1, 0.1)	0.84 (0.0012)	0.01 (0.0003)	0.87 (0.0001)	0.98 (0.0004)	0.94 (0.0014)	0.79 (0.0003)
(0.01, 0.01)	0.84 (0.0003)	0.01 (0.0001)	0.87 (0.0002)	0.98 (0.0001)	0.94 (0.0002)	0.79 (0.0001)
(0.01, 1)	0.84 (0.0003)	0.01 (0.0001)	0.87 (0.0003)	0.98 (0.0002)	0.94 (0.0004)	0.79 (0.0001)
(1, 0.01)	0.84 (0.0004)	0.01 (0.0000)	0.87 (0.0003)	0.98 (0.0001)	0.94 (0.0002)	0.79 (0.0001)
(1, 1)	0.84 (0.0005)	0.01 (0.0001)	0.87 (0.0004)	0.98 (0.0001)	0.94 (0.0003)	0.79 (0.0001)

Table S6: Sensitivity analysis of B-MASTER under different hyperparameter choices for (δ_1, δ_2). Mean measure values are provided, with standard errors in parentheses, based on 10 simulation experiments.

to moderate perturbations of the global–shrinkage hyper–rates δ_1 and δ_2, while the shape hyperparameters $r_1 = r_2 = 1$ are effectively non–informative in the regimes studied.

S4.3 Sensitivity to MCMC Iteration Length

To further assess whether the reported performance is sensitive to the total number of MCMC iterations, we repeated the real data–based simulation study under three sampling lengths: 500, 1000, and 2000 total iterations, each with the first 100 iterations discarded as burn-in. This analysis directly evaluates whether the default sampling regime used in the main simulation study is sufficient for stable variable-selection performance.

Num. MCMC iters	TPR	FPR	MCC	AUC	AUC20	Sparsity (True = 0.76)
500	0.835	0.009	0.870	0.981	0.936	0.791
1000	0.836	0.009	0.871	0.982	0.937	0.791
2000	0.836	0.008	0.870	0.981	0.936	0.791

Table S7: Sensitivity analysis for the total number of MCMC iterations in the B-MASTER simulation study. Results are reported for MCMC iteration lengths of 500, 1000, and 2000, each using a fixed burn-in of 100 iterations. The metrics include true positive rate (TPR), false positive rate (FPR), Matthews correlation coefficient (MCC), area under the ROC curve (AUC), partial AUC restricted to low false positive regions (AUC20), and estimated sparsity of the coefficient matrix. The true sparsity level in the simulated setting was 0.76.

Figures S4–S6 show trace plots for the two global shrinkage parameters, λ_1^2 and λ_2^2, and five representative regression coefficients corresponding to the largest absolute entries of the estimated coefficient matrix. Across the three iteration lengths, the displayed chains show stable post–burn-in behavior. The global shrinkage parameter λ_1^2 rapidly concentrates near zero, reflecting strong shrinkage rather than lack of convergence, while λ_2^2 and the representative regression coefficients fluctuate around stable posterior regions. The MCSE/SD

ratios and Geweke diagnostics reported in the figure panels provide additional evidence that the posterior summaries are not driven by poor mixing.

Table S7 summarizes the corresponding variable-selection performance. The performance metrics are nearly unchanged across 500, 1000, and 2000 iterations: TPR remains approximately 0.835–0.836, FPR remains approximately 0.008–0.009, MCC remains approximately 0.870–0.871, AUC remains approximately 0.981–0.982, AUC20 remains approximately 0.936–0.937, and the estimated sparsity remains 0.791 across all three settings. These results indicate that the posterior edge-selection results are stable with respect to the total number of MCMC iterations within the range considered. Thus, the computational advantages reported in the main text do not appear to be an artifact of an insufficient sampling length.

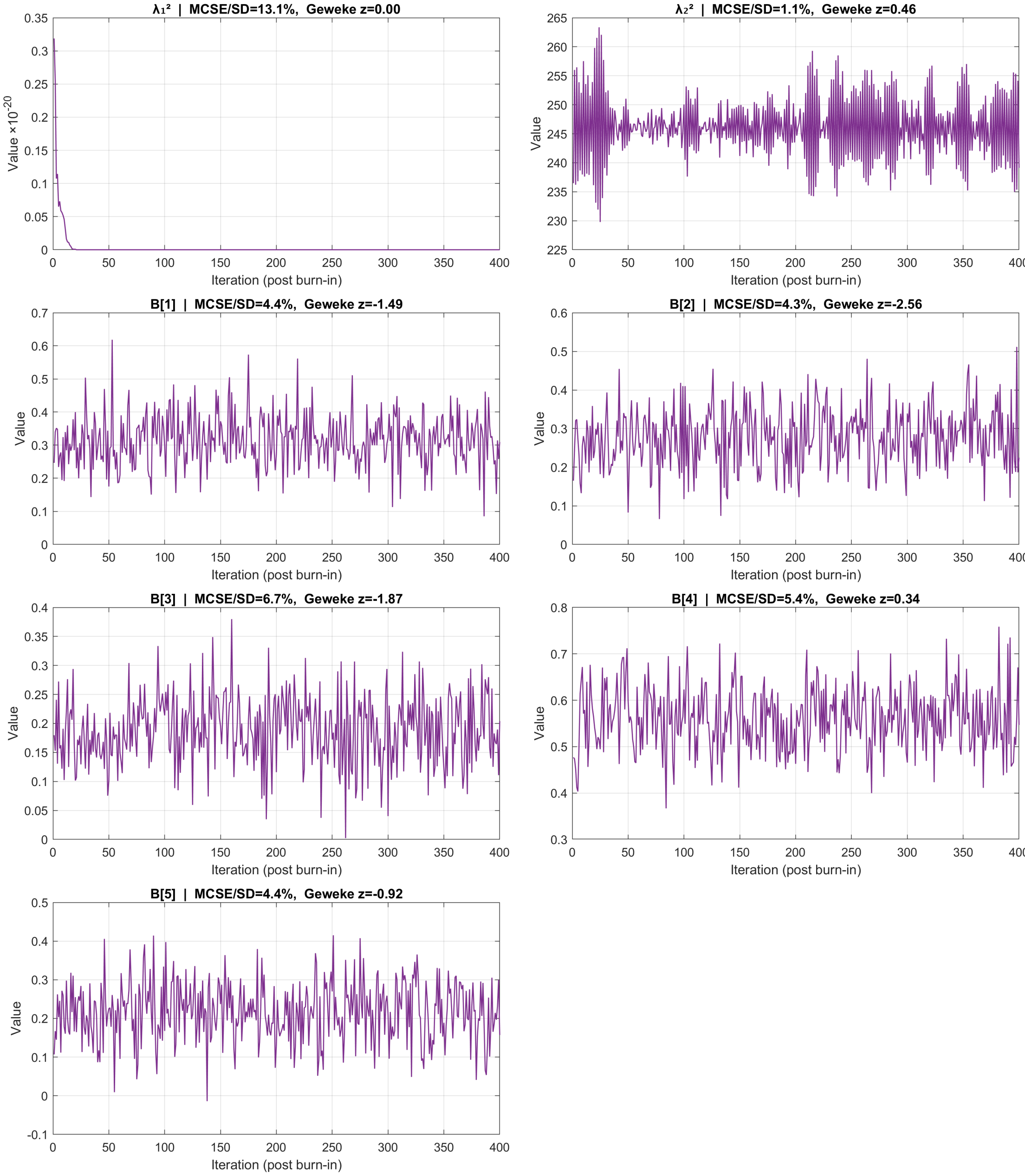

Figure S4: Trace plots from the B-MASTER real-data analysis using 500 total MCMC iterations with the first 100 iterations discarded as burn-in. Shown are the chains for λ_1^2, λ_2^2, and five representative regression coefficients ($B[1]$–$B[5]$), selected as the five largest absolute entries of the estimated coefficient matrix B. For each parameter, the Monte Carlo standard error relative to posterior variability (MCSE/SD%) and Geweke stationarity diagnostic z-score are reported in the panel titles. Across most displayed parameters, the chains exhibit stable post–burn-in behavior, relatively low MCSE/SD values ($< 10\%$), and Geweke statistics close to the commonly used range $|z| \leq 2$, indicating generally satisfactory mixing and convergence behavior for this shorter MCMC run.

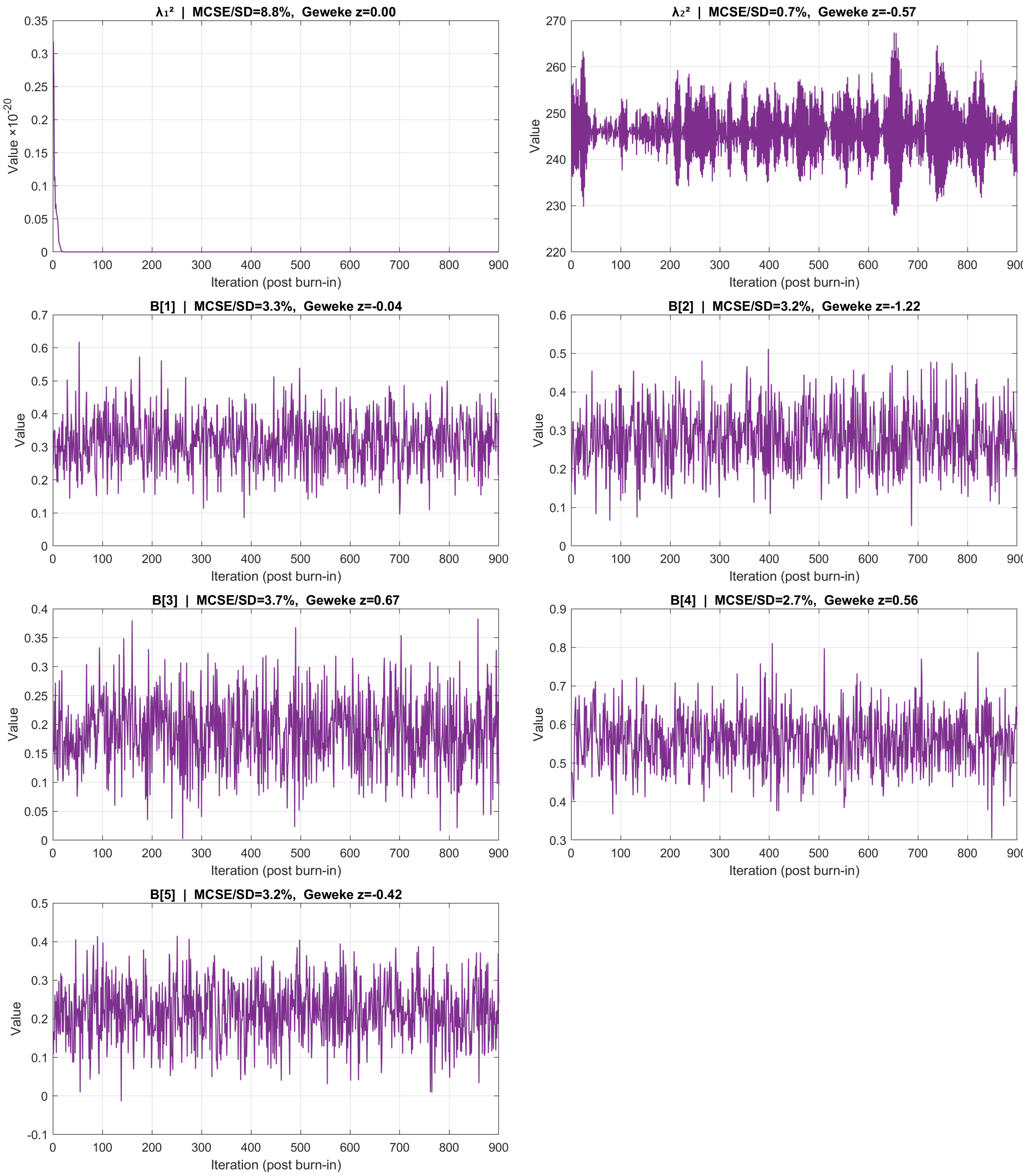

Figure S5: Trace plots from the B-MASTER real-data analysis using 1000 total MCMC iterations with the first 100 iterations discarded as burn-in. Shown are the chains for λ_1^2, λ_2^2, and five representative regression coefficients ($B[1]$–$B[5]$), selected as the five largest absolute entries of the estimated coefficient matrix B. For each parameter, the Monte Carlo standard error relative to posterior variability (MCSE/SD%) and Geweke stationarity diagnostic z-score are reported in the panel titles. Across all displayed parameters, the chains exhibit stable post–burn-in behavior, low relative MCSE values, and Geweke statistics within the commonly used range $|z| \leq 2$, supporting satisfactory mixing and convergence of the Gibbs sampler.

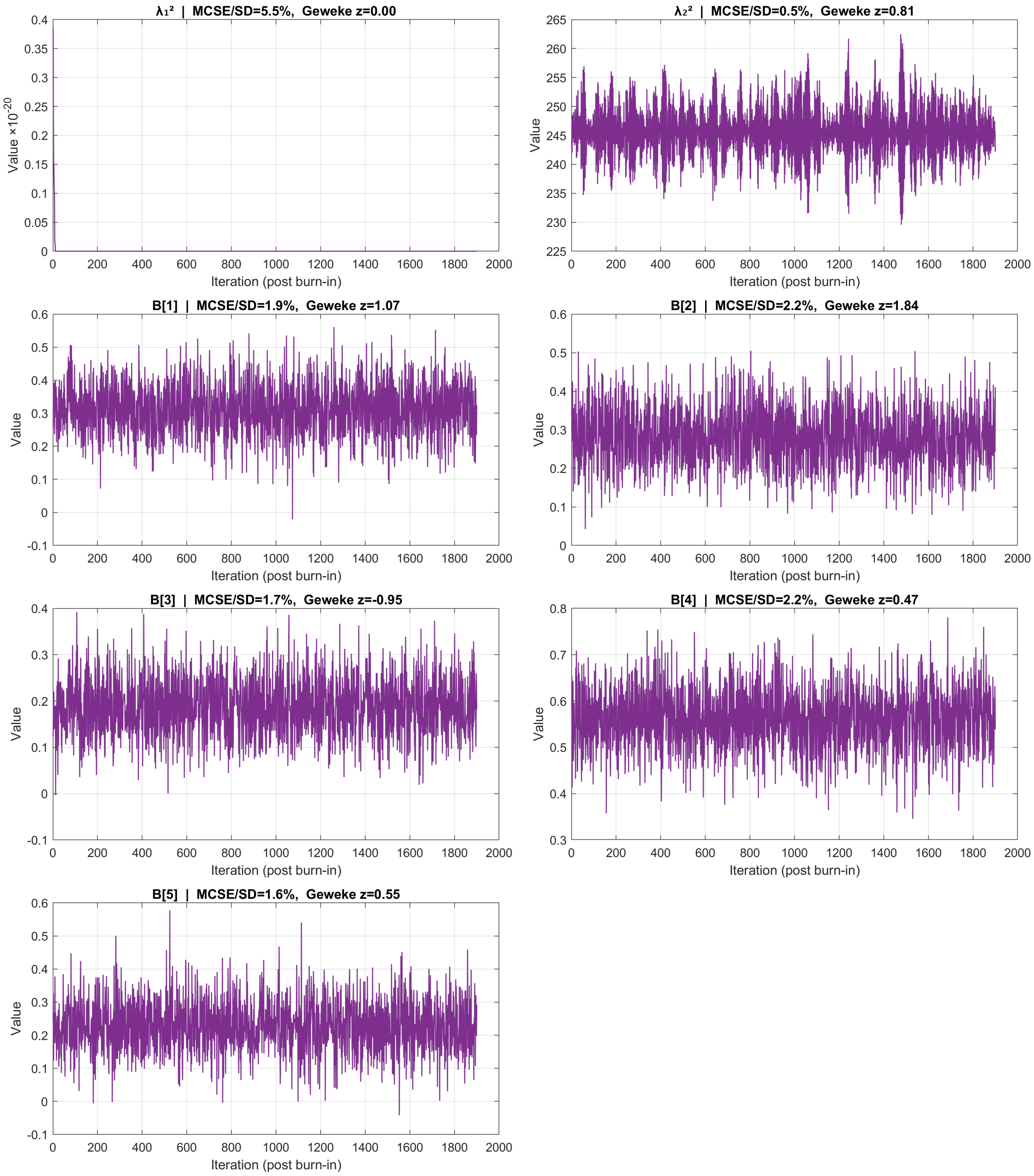

Figure S6: Trace plots from the B-MASTER real-data analysis using 2000 total MCMC iterations with the first 100 iterations discarded as burn-in. Shown are the chains for λ_1^2, λ_2^2, and five representative regression coefficients ($B[1]$–$B[5]$), selected as the five largest absolute entries of the estimated coefficient matrix B. For each parameter, the Monte Carlo standard error relative to posterior variability (MCSE/SD%) and Geweke stationarity diagnostic z-score are reported in the panel titles. Across all displayed parameters, the chains exhibit stable post–burn-in behavior, low relative MCSE values ($< 10\%$), and Geweke statistics within the commonly used range $|z| \leq 2$, supporting satisfactory mixing and convergence of the Gibbs sampler.

S5 B-MASTER Case Study

S5.1 Descriptive statistics and diagnostics

The CRC case study cohort consisted of $N = 220$ subjects with paired microbiome and metabolomic measurements, including $P = 287$ microbial genera and $Q = 249$ metabolites. Supplementary Figure S7 summarizes the distributions of key demographic and lifestyle variables. The cohort exhibited broad age and BMI distributions, while the sex distribution was moderately balanced. Smoking and alcohol-consumption variables also showed substantial heterogeneity across subjects. Supplementary Figure S8 presents residual normality diagnostics obtained from preliminary marginal regression analyses. The pooled QQ-plot in Figure S8a demonstrates noticeable departures from Gaussian behavior in the tails, while the metabolite-wise kurtosis distribution in Figure S8b indicates frequent excess kurtosis across outcomes. These observations suggest that the metabolomic responses exhibit moderate heavy-tailed behavior and dependence heterogeneity.

To further investigate dependence structures within the dataset, Supplementary Figure S9 displays correlation heatmaps among metabolites and between microbiome and metabolomic features. Figure S9a reveals strong block-wise correlation structures among metabolites, suggesting the presence of coordinated metabolic pathways and substantial cross-response dependence. Figure S9b similarly demonstrates nontrivial cross-domain association patterns between microbial genera and metabolites, with localized clusters of elevated correlations. These observations motivate a joint multivariate modeling strategy rather than independent univariate analyses across metabolites. Supplementary Figure S10 presents principal component analysis (PCA) score plots for the microbiome and metabolomic feature spaces. The PCA projections suggest substantial variability and heterogeneity across subjects without strong low-dimensional separation patterns dominating the dataset. This diffuse structure supports the need for sparse high-dimensional modeling approaches capable of identifying localized association structures embedded within complex multivariate dependence patterns, rather than relying solely on low-rank global latent representations.

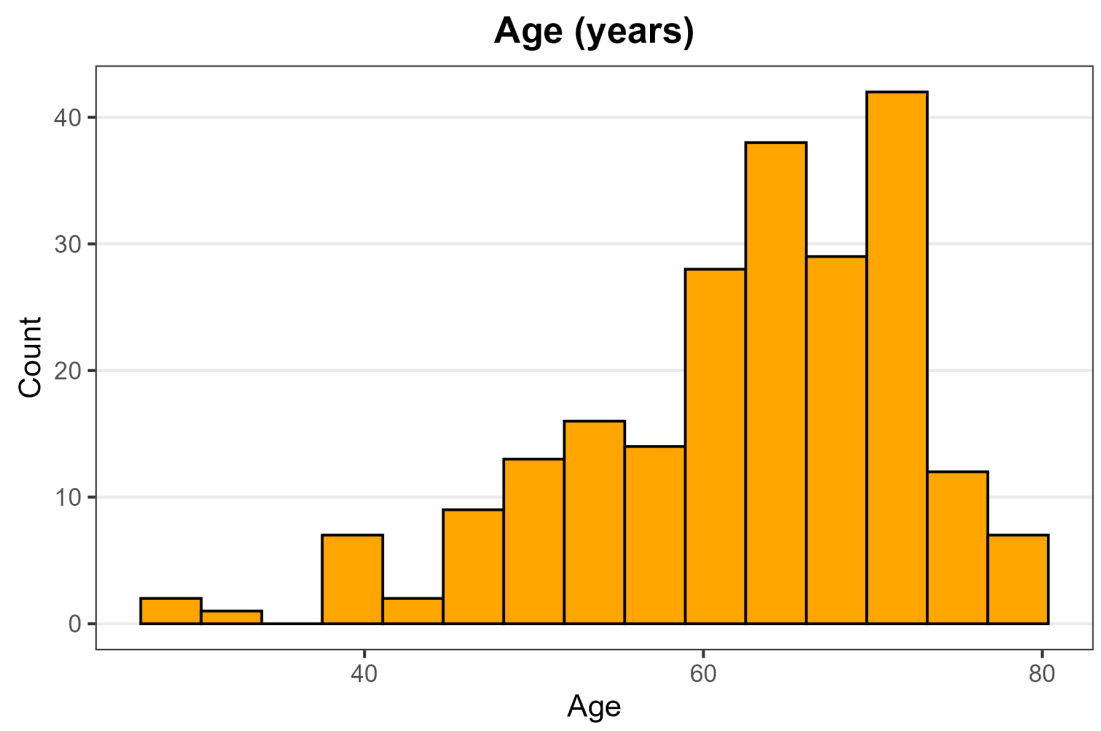

(a) Age distribution

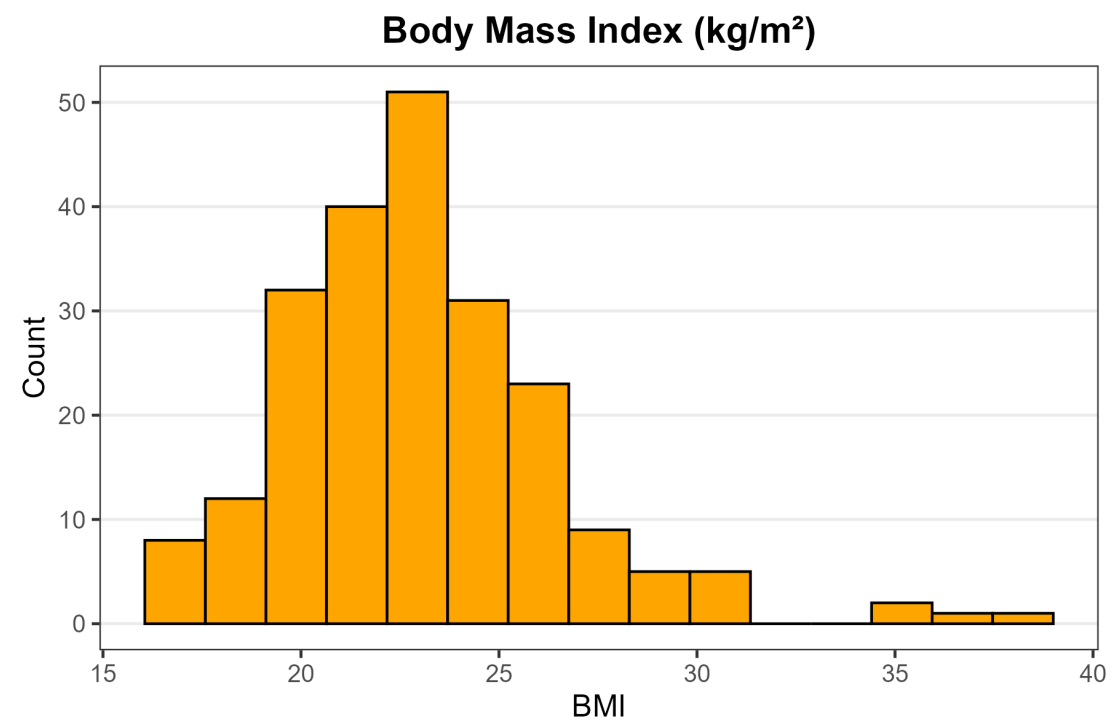

(b) Body Mass Index (BMI)

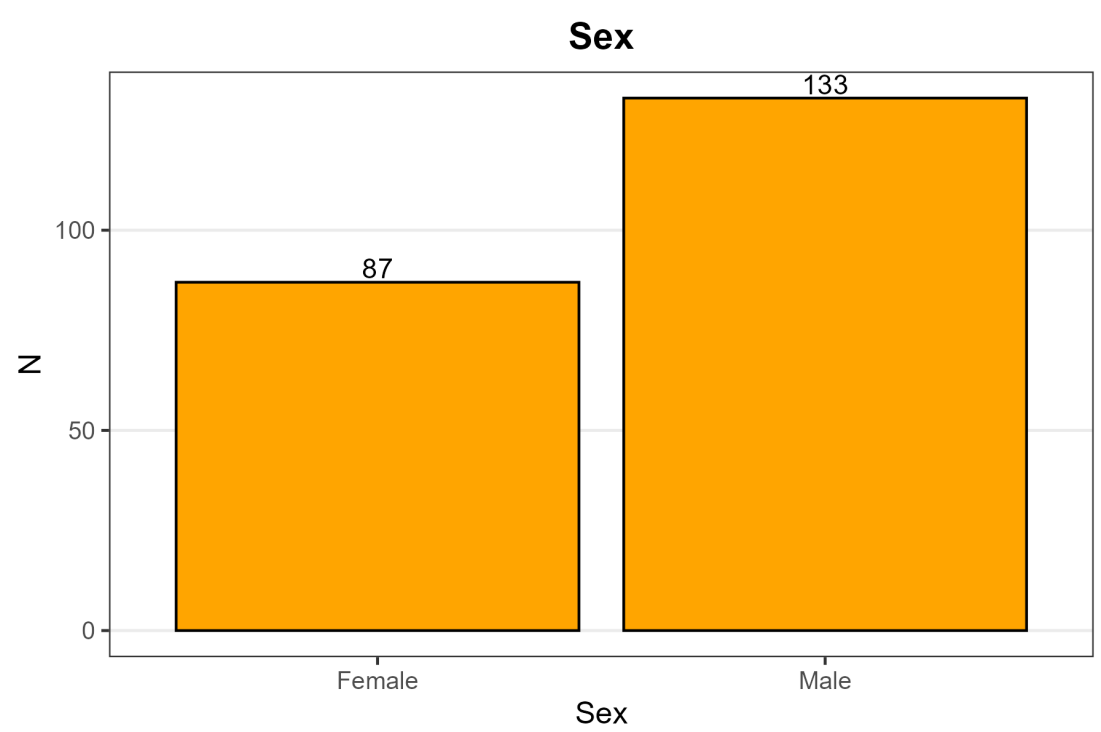

(c) Sex distribution

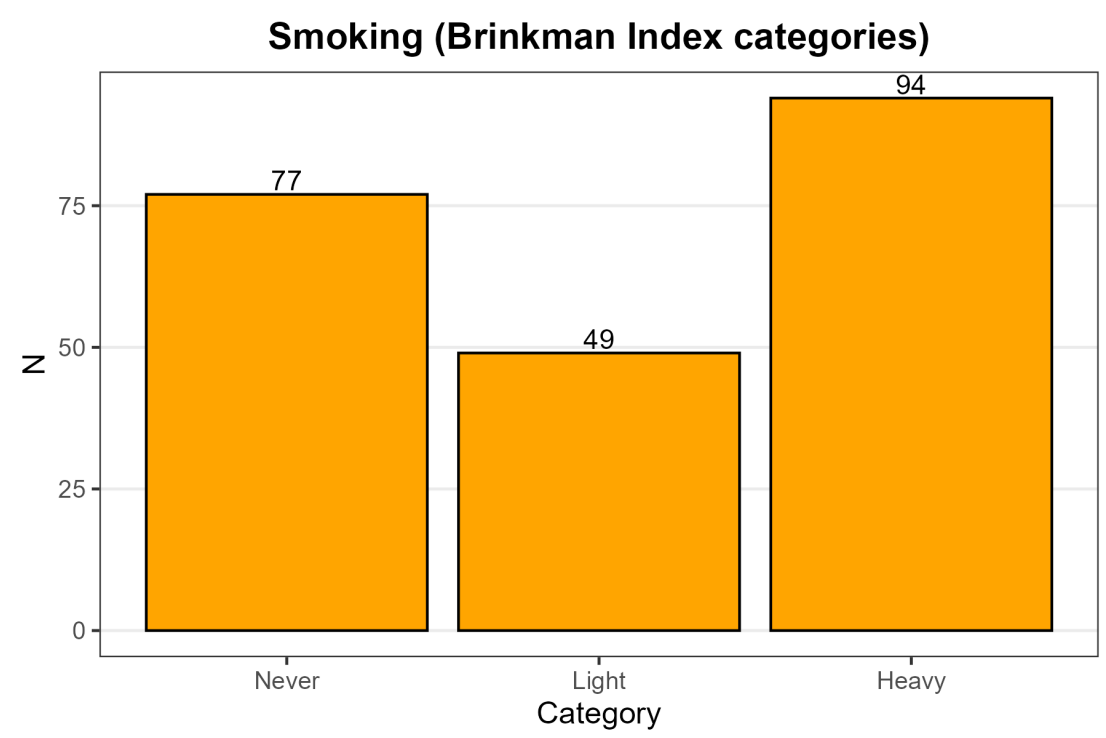

(d) Smoking categories

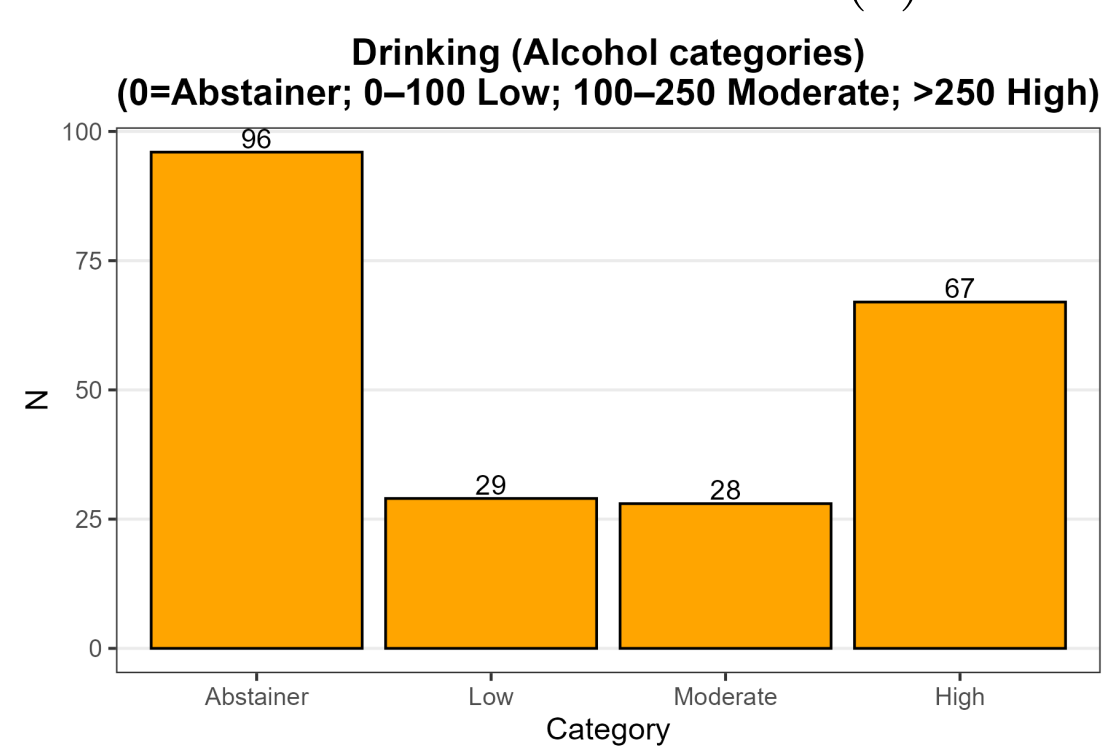

(e) Alcohol consumption categories

Figure S7: Supplementary descriptive plots of patient characteristics in the case study cohort. Distributions are shown for (a) age, (b) BMI, (c) sex, (d) smoking, and (e) drinking.

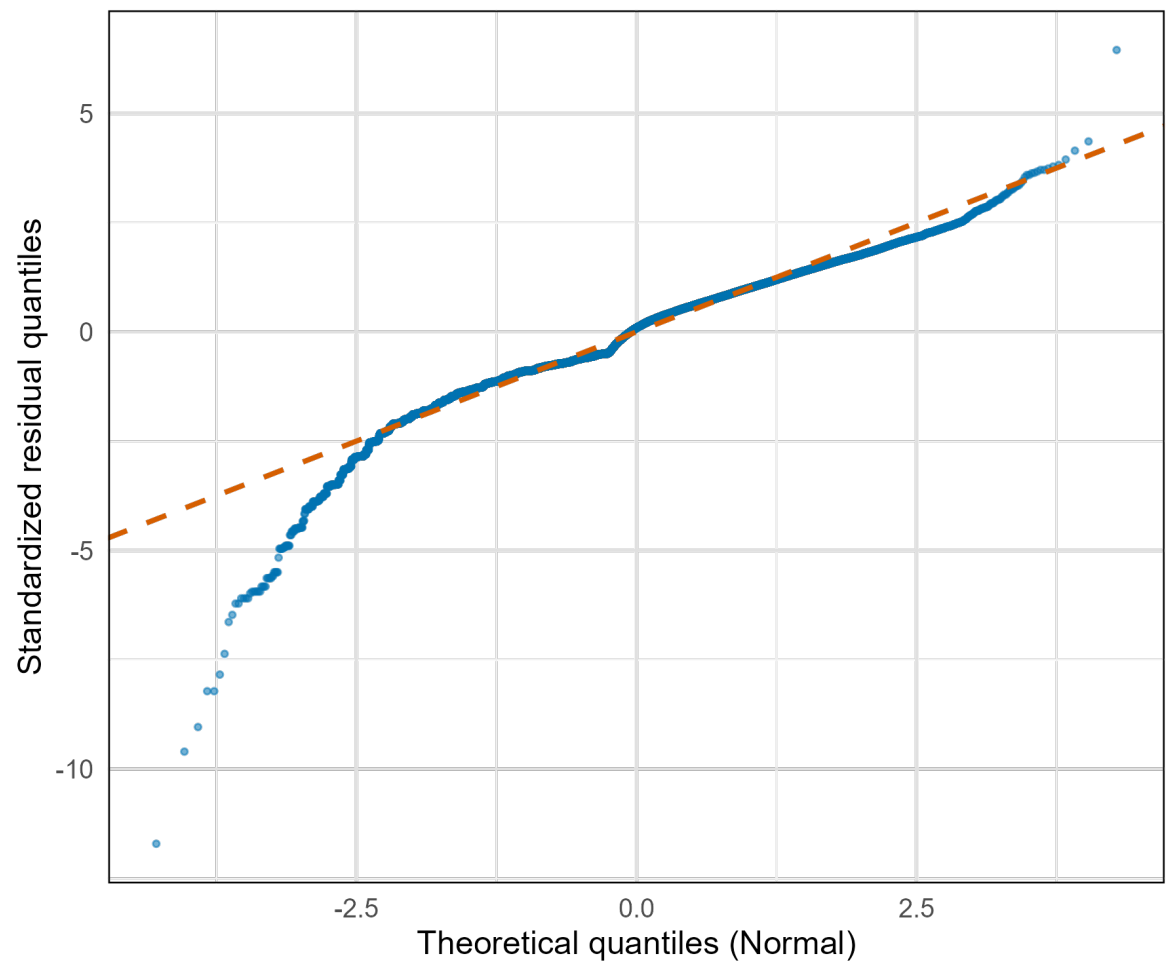

(a) QQ-plot of standardized residuals showing heavy tails relative to Gaussian.

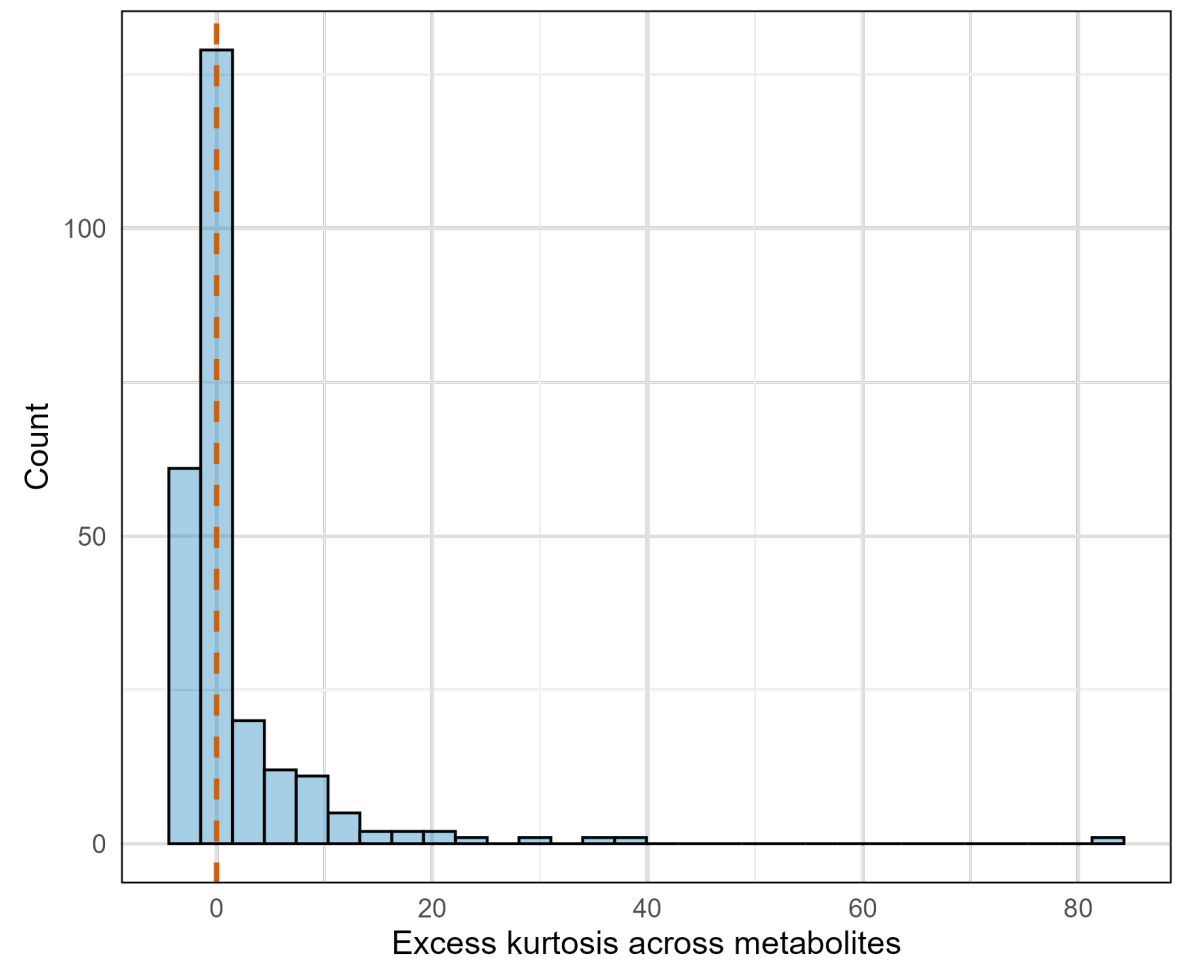

(b) Histogram of residual kurtosis across metabolites.

Figure S8: Normality diagnostics of residuals. Panel (a) highlights deviations from normality in a pooled QQ-plot, while panel (b) shows excess kurtosis across metabolites.

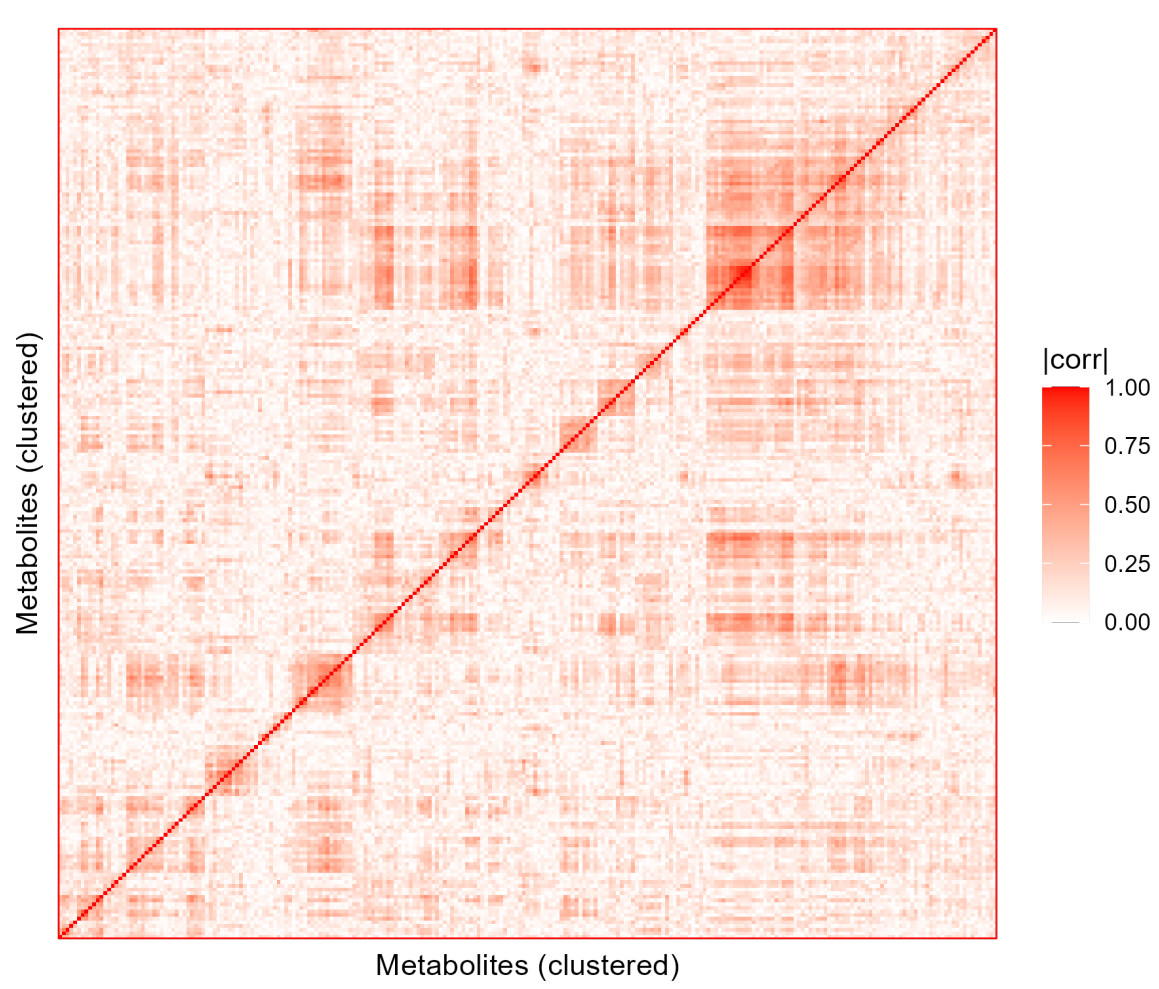

(a) Absolute correlation heatmap among all metabolites (Y).

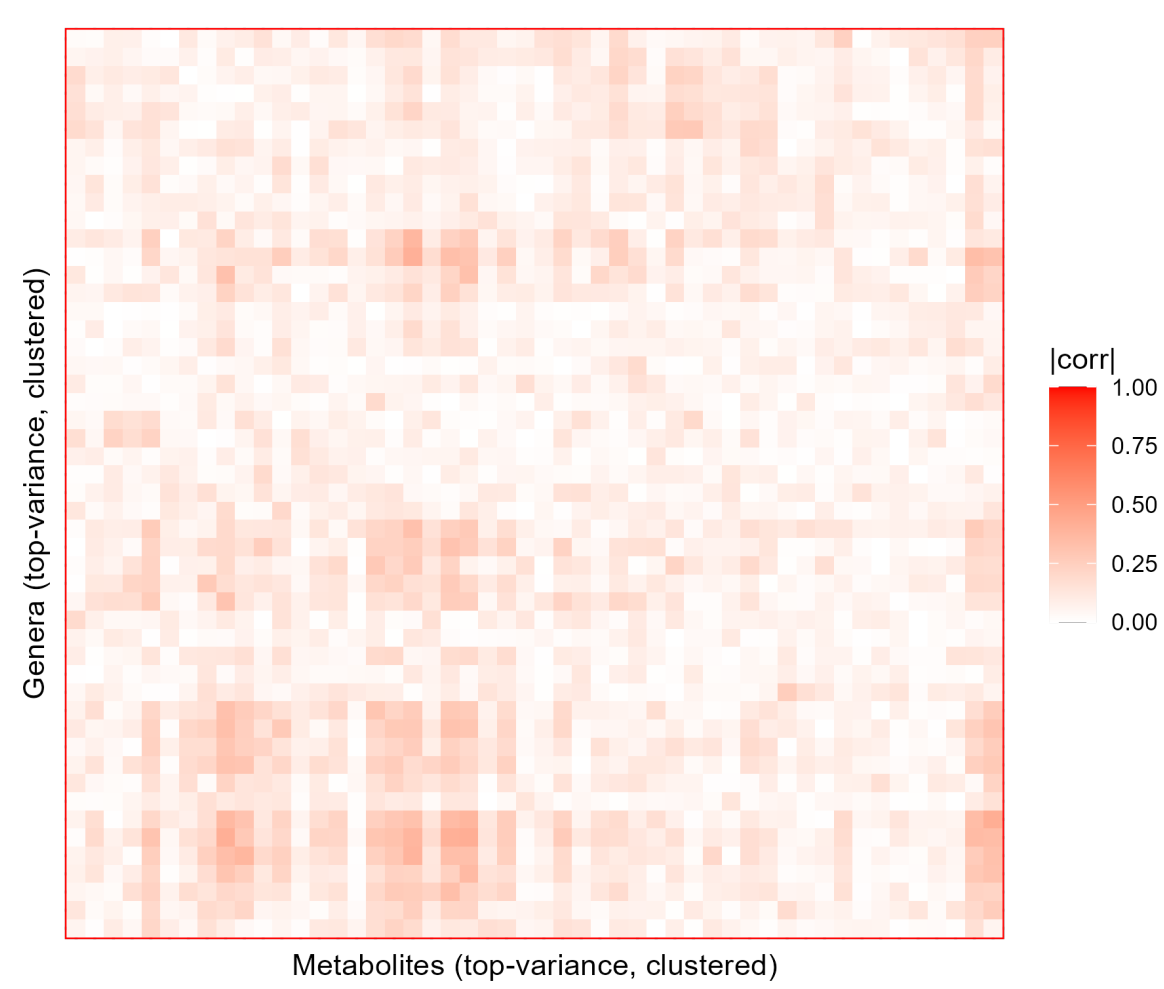

(b) Absolute correlation between the top 50 variance-selected genera (X) and top 50 variance-selected metabolites (Y).

Figure S9: Correlation diagnostics for the CRC dataset. (a) Heatmap of absolute correlations across all metabolites, showing block structures indicative of dependence. (b) Cross-correlation heatmap between the top 50 genera and top 50 metabolites, selected by variance and clustered within each domain. These plots reveal strong dependence both within and across microbiome and metabolome features, motivating a framework capable of handling correlated predictors and responses.

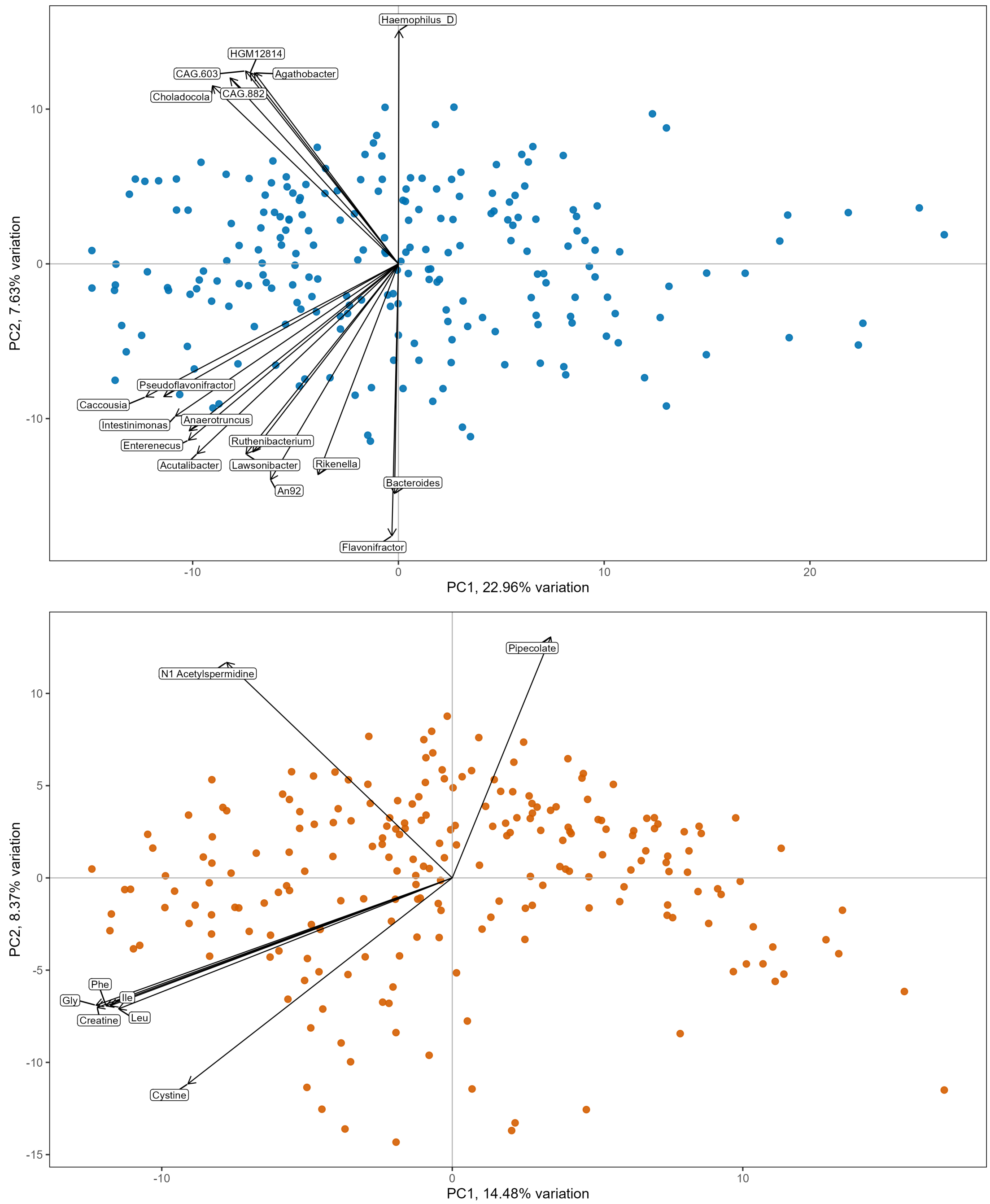

Figure S10: Score plots from Principal Component Analysis (PCA) illustrating the distribution of $P = 287$ microbial genera (top) and $Q = 249$ metabolites (bottom) based on fecal samples collected from $N = 220$ patients.

S5.2 Fractional Influence Score (FIS)

To provide an interpretable measure of how influence is distributed across metabolites, we introduce a *Fractional Influence Score (FIS)* for each microbial genus identified by B-MASTER. For any metabolite outcome Y_q, suppose it is influenced by h_q genera (as determined by the B-MASTER selection step). Instead of attributing a full unit of influence to every selected genus, we allocate equal shares: each contributing genus receives a score of $1/h_q$. Summing these fractional contributions across all metabolites yields the overall score for a genus,

$$\text{FIS}(g) = \sum_{q=1}^{Q} \frac{\mathbf{1}\{g \in \mathcal{I}_q\}}{h_q},$$

where $\mathcal{I}_q$ is the set of influencing genera for metabolite q. Because h_q appears in the denominator, this score naturally distinguishes genera that uniquely regulate a metabolite from those that appear only as part of larger sets of co-predictors. The full distribution of genus-specific Fractional Influence Scores (FIS) across all 287 genera is summarized in Tables S8–S13. These tables were included to provide a comprehensive characterization of the inferred influence hierarchy beyond the subset displayed in the main manuscript figures. In particular, they allow assessment of how influence is distributed across the complete microbiome set, the extent to which high-ranking genera are separated from lower-ranking genera, and the robustness of the inferred rankings under increasingly stringent posterior selection criteria.

Several observations emerge from these summaries. First, although the FIS values decrease gradually rather than exhibiting a single abrupt cutoff, the cumulative FIS percentages indicate meaningful concentration of influence among the top-ranked genera. Specifically, the top 50 genera accounted for approximately 24.85% of the total cumulative FIS despite representing only about 17% of all genera. This enrichment suggests that influence is not uniformly distributed across the microbiome network. Rather, a relatively small subset of genera repeatedly appears across many metabolite-specific posterior selection sets and therefore accumulates disproportionately large influence contributions.

Second, the ranking structure exhibits substantial stability under stricter posterior selection thresholds. Across Tables S8–S13, many of the highest-ranked genera under the default 90% posterior criterion remain highly ranked under the 95% and 99% posterior criteria. For example, several top-ranked genera including *Alistipes_A*, *Ligilactobacillus*,

Veillonella, *Mogibacterium*, and *Massiliomicrobiota* continued to exhibit strong FIS values and relatively stable rankings under stricter posterior thresholds. This stability provides an additional layer of uncertainty quantification for the inferred master predictors and suggests that the dominant signals are not driven by isolated weak posterior selections.

Third, the median Bayesian posterior p-values reported in Tables S8–S13 provide a complementary measure of posterior edge-level support. Genera with high FIS values generally exhibited small median posterior p-values across their selected metabolite associations, indicating that the observed influence structure is supported not only by repeated selection frequency but also by strong posterior evidence at the metabolite-association level. Importantly, the Bayesian p-values summarize uncertainty for the selected microbiome-metabolite relationships, while the multi-threshold FIS summaries (90%, 95%, and 99%) provide an indirect uncertainty assessment for the genus-level influence rankings themselves.

The choice to focus on the top 50 genera in the main manuscript was therefore intended primarily as a visualization and interpretability device rather than as a strict biological cutoff. The full tables demonstrate that the FIS hierarchy extends continuously across all genera, while still revealing a practically meaningful concentration of influence among the highest-ranked genera. Together, these results support the utility of the FIS framework as a quantitative measure for identifying influential microbial master predictors within the microbiome-metabolite network inferred by B-MASTER.

Genus	FIS rank (default; 90% CI based)	FIS (default; 90% CI based)	Cumulative FIS (%)	Num. Selected metabolites	Median Bayesian p-value (default; 90% CI-based)	FIS (95% CI based)	Rank (95% CI based)	FIS (99% CI based)	Rank (99% CI based)
CAG.196	1	1.45	0.58	136	0.009	1.58	3	1.94	4
Choladocola	2	1.42	1.15	133	0.009	1.44	12	1.87	8
Alistipes_A	3	1.4	1.72	131	0.002	1.63	1	2.32	1
Veillonella	4	1.4	2.28	130	0.008	1.5	8	1.93	5
Ligilactobacillus	5	1.38	2.83	129	0.004	1.6	2	2.1	2
Agathobacter	6	1.36	3.38	126	0.009	1.55	4	1.69	17
Mogibacterium	7	1.36	3.92	127	0.009	1.54	5	1.88	6
Massiliomicrobiota	8	1.35	4.46	126	0.007	1.46	10	1.96	3
Caccousia	9	1.34	5	126	0.007	1.39	18	1.73	15
Succinivibrio	10	1.32	5.53	123	0.011	1.45	11	1.65	19
Phil1	11	1.31	6.06	123	0.011	1.41	17	1.68	18
Gemmiger	12	1.31	6.58	123	0.007	1.51	7	1.85	9
Anaerotignum	13	1.31	7.11	123	0.004	1.54	6	1.87	7
Egerieisoma	14	1.3	7.63	121	0.009	1.39	19	1.76	11
UBA3402	15	1.29	8.15	121	0.013	1.35	21	1.53	27
Fimenecus	16	1.28	8.67	120	0.013	1.44	14	1.56	23
Avimicrobium	17	1.28	9.18	120	0.009	1.44	13	1.63	20
Merdisoma	18	1.28	9.7	119	0.009	1.43	15	1.75	13
Enterocloster	19	1.27	10.21	119	0.016	1.32	26	1.43	39
CAG.83	20	1.26	10.72	118	0.009	1.49	9	1.74	14
UBA737	21	1.25	11.22	117	0.011	1.38	20	1.53	26
Gemella	22	1.24	11.72	116	0.018	1.19	51	1.35	51
Pauljensenia	23	1.22	12.21	114	0.007	1.33	24	1.75	12
HGM12814	24	1.22	12.7	114	0.01	1.41	16	1.53	25
Butyricicoccus_A	25	1.21	13.18	113	0.007	1.34	22	1.78	10
CAG.317	26	1.2	13.67	112	0.013	1.25	36	1.37	49
Streptococcus	27	1.2	14.15	112	0.013	1.29	28	1.36	50
Bariatricus	28	1.2	14.63	112	0.009	1.22	45	1.57	22
Anaerotruncus	29	1.19	15.11	111	0.011	1.25	39	1.46	33
Fusobacterium_B	30	1.19	15.59	111	0.013	1.29	30	1.4	43
Faecalimonas	31	1.19	16.06	112	0.007	1.34	23	1.72	16
Onthousia	32	1.19	16.54	110	0.016	1.23	42	1.31	54
Limosilactobacillus	33	1.19	17.02	111	0.013	1.16	57	1.4	41
Amedibacterium	34	1.18	17.49	111	0.009	1.26	35	1.52	29
Mitsuokella	35	1.18	17.97	110	0.014	1.25	37	1.5	30
CAG.95	36	1.18	18.44	110	0.009	1.21	46	1.52	28
Turicibacter	37	1.17	18.91	109	0.016	1.27	32	1.34	52
CAG.177	38	1.16	19.38	108	0.018	1.16	58	1.12	85
Phascolarctobacterium_A	39	1.15	19.84	108	0.009	1.27	34	1.48	31
Eubacterium_F	40	1.15	20.3	107	0.007	1.27	33	1.6	21
Romboutsia	41	1.14	20.76	105	0.018	1.13	63	1.2	72
Butyricicoccus	42	1.14	21.21	107	0.018	1.12	66	1.26	65
Merdicola	43	1.14	21.67	105	0.011	1.22	44	1.4	42
Clostridium_AQ	44	1.14	22.13	106	0.014	1.2	48	1.23	69
Sellimonas	45	1.14	22.58	107	0.011	1.3	27	1.47	32
Bacteroides	46	1.13	23.04	106	0.013	1.18	54	1.3	55
Lawsonibacter	47	1.13	23.49	106	0.011	1.29	29	1.38	47
Alistipes	48	1.13	23.95	105	0.018	1.1	69	1.29	57
Granulicatella	49	1.13	24.4	105	0.016	1.22	43	1.39	46
CAG.267	50	1.13	24.85	105	0.016	1.18	53	1.28	59

Table S8: Top 50 genera ranked by Fractional Influence Score (FIS) under the default 90% posterior selection criterion in the CRC cohort. The table reports genus-specific FIS rankings, cumulative FIS contribution, number of selected metabolites connected to each genus, median Bayesian posterior edge significance, and corresponding FIS/rank summaries obtained under stricter 95% and 99% posterior selection criteria. These results illustrate the dominant CRC-associated master predictors identified by B-MASTER and demonstrate substantial ranking stability across posterior selection thresholds.

Genus	FIS rank (default; 90% CI based)	FIS (default; 90% CI based)	Cumulative FIS (%)	Num. Selected metabolites	Median Bayesian p-value (default; 90% CI-based)	FIS (95% CI based)	Rank (95% CI based)	FIS (99% CI based)	Rank (99% CI based)
Lancefieldella	51	1.13	25.3	105	0.011	1.14	62	1.39	45
Copromonas	52	1.12	25.75	105	0.018	1.17	55	1.28	60
1XD42.69	53	1.12	26.2	105	0.009	1.32	25	1.44	35
Enterococcus	54	1.12	26.65	103	0.009	1.25	40	1.45	34
UMGS872	55	1.12	27.1	105	0.011	1.21	47	1.41	40
Gordonibacter	56	1.11	27.55	104	0.011	1.25	41	1.38	48
Intestinibacter	57	1.11	28	103	0.016	1.14	60	1.29	56
CAG.239.g__51.20	58	1.11	28.44	103	0.009	1.25	38	1.56	24
CAG.603	59	1.11	28.89	104	0.013	1.18	52	1.27	62
Butyribacter	60	1.11	29.33	104	0.013	1.16	56	1.34	53
Slackia_A	61	1.1	29.78	103	0.016	1.15	59	1.19	73
UBA4372	62	1.1	30.22	103	0.013	1.13	64	1.22	71
Oliverpabstia	63	1.09	30.65	101	0.007	1.2	49	1.44	37
Mailhella	64	1.09	31.09	101	0.009	1.28	31	1.44	36
Eubacterium_R	65	1.07	31.52	100	0.02	1.02	90	1.15	78
Porphyromonas	66	1.07	31.95	100	0.018	1.03	88	0.98	105
Ruminococcus_D	67	1.06	32.38	99	0.013	1.14	61	1.26	64
Angelakisella	68	1.06	32.8	99	0.022	1.09	72	1.01	99
An92	69	1.06	33.23	99	0.013	1.11	67	1.25	66
Lachnospiraceae.g__14.2	70	1.06	33.65	99	0.016	1.03	86	1.13	81
CAG.1031	71	1.05	34.07	98	0.014	1.06	83	1.12	83
Acetatifactor	72	1.05	34.49	98	0.018	1.08	73	1.01	98
MWCK01	73	1.04	34.91	97	0.013	1.11	68	1.01	100
Bilophila	74	1.04	35.33	96	0.009	1.19	50	1.4	44
Copromorpha	75	1.03	35.74	97	0.011	1.06	80	1.27	61
Streptomyces	76	1.02	36.15	96	0.018	1.09	70	1.08	90
Oxalobacter	77	1.02	36.56	95	0.02	1.06	82	1.16	76
F23.B02	78	1.01	36.97	95	0.013	1.08	75	1.15	77
Flavonifractor	79	1.01	37.37	94	0.016	1	93	1.05	93
Faecalitalea	80	1	37.78	94	0.028	0.9	123	0.92	117
UMGS856	81	1	38.18	93	0.022	1.03	84	0.98	106
Fimivicinus	82	1	38.58	93	0.02	0.99	100	1	102
Agathobaculum	83	1	38.98	94	0.018	1.07	79	1.13	82
Odoribacter	84	1	39.38	94	0.011	1.09	71	1.23	68
Clostridium	85	1	39.78	93	0.013	1.06	81	1.13	80
CAG.488	86	1	40.19	93	0.022	1.08	77	0.77	148
Eggerthella	87	1	40.59	93	0.011	1.13	65	1.24	67
Lactococcus	88	1	40.99	93	0.018	0.97	103	1.08	89
Intestinimonas	89	0.99	41.38	93	0.013	1.08	74	1.14	79
Blautia_A	90	0.99	41.78	93	0.027	0.93	117	0.8	138
Schaedlerella	91	0.99	42.18	92	0.013	1.03	87	1.03	95
Phil12	92	0.98	42.57	91	0.022	0.91	120	0.78	145
NSJ.51	93	0.98	42.96	91	0.009	1.01	91	1.28	58
NSJ.61	94	0.98	43.36	91	0.016	0.99	97	1.11	86
Evtepia	95	0.97	43.75	91	0.011	0.98	101	1.18	74
Lacrimispora	96	0.97	44.14	91	0.016	0.95	110	0.98	107
CAG.266	97	0.97	44.53	90	0.016	1	94	1.08	91
Holdemania	98	0.96	44.91	90	0.013	1	96	1.05	94
Limisoma	99	0.96	45.3	90	0.016	1.03	85	1.03	96
Dorea	100	0.96	45.69	90	0.007	1.07	78	1.43	38

Table S9: Genera ranked 51–100 by Fractional Influence Score (FIS) under the default 90% posterior selection criterion in the CRC cohort. The table reports genus-specific FIS rankings, cumulative FIS contribution, number of selected metabolites connected to each genus, median Bayesian posterior edge significance, and corresponding FIS/rank summaries obtained under stricter 95% and 99% posterior selection criteria.

Genus	FIS rank (default; 90% CI based)	FIS (default; 90% CI based)	Cumulative FIS (%)	Num. Selected metabolites	Median Bayesian p-value (default; 90% CI-based)	FIS (95% CI based)	Rank (95% CI based)	FIS (99% CI based)	Rank (99% CI based)
Parvimonas	101	0.96	46.07	89	0.029	0.89	125	0.94	116
Tidjanibacter	102	0.96	46.46	89	0.016	1	95	0.96	112
Coprobacter	103	0.95	46.84	89	0.013	1.08	76	1.11	87
CAG.41	104	0.95	47.22	89	0.018	0.95	111	0.88	126
Negativibacillus	105	0.95	47.6	89	0.011	0.95	107	1.22	70
Scatomorpha	106	0.95	47.98	88	0.009	0.99	98	1.27	63
Lachnospira	107	0.94	48.36	88	0.017	0.99	99	0.96	111
Fusobacterium	108	0.94	48.74	88	0.018	0.93	116	0.95	114
UBA1417	109	0.93	49.11	87	0.011	1.02	89	1.12	84
CAG.269	110	0.93	49.49	87	0.024	0.86	137	0.7	165
Rothia	111	0.93	49.86	87	0.016	0.9	121	1.07	92
PeH17	112	0.93	50.23	86	0.014	1.01	92	0.97	110
Megasphaera	113	0.92	50.6	86	0.02	0.95	109	0.84	133
Bulleidia	114	0.92	50.97	86	0.016	0.98	102	0.97	108
Faecousia	115	0.92	51.34	86	0.019	0.96	106	0.86	129
Cryptobacteroides	116	0.92	51.71	86	0.016	0.95	112	1	103
Thauera	117	0.92	52.08	85	0.018	0.96	104	0.9	121
Zag111	118	0.91	52.45	85	0.011	0.89	126	1.02	97
Faecalibacterium	119	0.9	52.81	84	0.02	0.94	113	0.9	119
Faecimorpha	120	0.9	53.17	84	0.022	0.85	141	0.9	118
Pelethomonas	121	0.9	53.53	84	0.021	0.95	108	0.89	124
Acutalibacter	122	0.9	53.9	85	0.024	0.87	135	0.78	146
Clostridium_Q	123	0.9	54.26	85	0.029	0.87	130	0.53	212
UBA3818	124	0.9	54.62	84	0.009	0.96	105	1.17	75
Barnesiella	125	0.9	54.98	84	0.028	0.81	154	0.76	155
Gallimonas	126	0.9	55.34	84	0.029	0.76	171	0.74	161
Aphodousia	127	0.9	55.7	84	0.024	0.85	140	0.66	173
Cloacibacillus	128	0.9	56.06	84	0.013	0.87	136	0.98	104
Eubacterium_I	129	0.89	56.42	82	0.02	0.88	129	0.77	149
Stercorousia	130	0.88	56.77	83	0.033	0.77	166	0.67	169
Faecalicoccus	131	0.88	57.13	83	0.018	0.82	152	0.95	115
Limivivens	132	0.88	57.48	81	0.02	0.83	147	0.77	151
UBA738	133	0.88	57.84	82	0.02	0.86	139	0.89	123
Pseudoruminococcus_A	134	0.88	58.19	81	0.022	0.89	127	0.85	131
Monoglobus	135	0.88	58.54	81	0.016	0.93	114	0.9	120
Lachnoclostridium_B	136	0.88	58.89	82	0.017	0.87	133	0.95	113
CAG.492	137	0.87	59.24	81	0.016	0.92	118	1	101
Anaerobutyricum	138	0.87	59.59	81	0.024	0.9	122	0.71	163
Mediterraneibacter_A	139	0.86	59.94	80	0.018	0.85	142	0.79	141
UMGS1071	140	0.86	60.28	81	0.016	0.9	124	0.89	122
CAG.1427	141	0.86	60.63	80	0.023	0.83	148	0.75	159
Phocaeicola_A	142	0.86	60.97	80	0.036	0.87	134	0.7	164
Emergencia	143	0.86	61.32	80	0.027	0.77	164	0.66	171
Lachnoclostridium_A	144	0.85	61.66	80	0.013	0.93	115	1.08	88
Anaeromassilibacillus	145	0.85	62	80	0.019	0.84	144	0.97	109
UBA11524	146	0.85	62.34	80	0.02	0.87	131	0.82	136
Limadaptatus	147	0.85	62.69	79	0.033	0.74	185	0.62	189
Ventrimonas	148	0.85	63.03	79	0.02	0.86	138	0.76	156
Megamonas	149	0.85	63.37	79	0.036	0.76	176	0.48	222
Dialister	150	0.85	63.71	79	0.022	0.87	132	0.47	226

Table S10: Genera ranked 101–150 by Fractional Influence Score (FIS) under the default 90% posterior selection criterion in the CRC cohort. The table reports genus-specific FIS rankings, cumulative FIS contribution, number of selected metabolites connected to each genus, median Bayesian posterior edge significance, and corresponding FIS/rank summaries obtained under stricter 95% and 99% posterior selection criteria.

Genus	FIS rank (default; 90% CI based)	FIS (default; 90% CI based)	Cumulative FIS (%)	Num. Selected metabolites	Median Bayesian p-value (default; 90% CI-based)	FIS (95% CI based)	Rank (95% CI based)	FIS (99% CI based)	Rank (99% CI based)
Prevotella	151	0.84	64.04	78	0.016	0.92	119	0.87	127
Muricomes	152	0.84	64.38	77	0.022	0.84	145	0.71	162
Choladousia	153	0.84	64.72	79	0.024	0.77	165	0.66	170
Coproplasma	154	0.83	65.05	78	0.029	0.79	160	0.77	147
NSJ.62	155	0.83	65.39	78	0.021	0.81	155	0.8	139
Limiplasma	156	0.83	65.72	77	0.018	0.84	143	0.74	160
CAG.882	157	0.82	66.05	77	0.02	0.79	158	0.87	128
Victivallis	158	0.82	66.38	76	0.031	0.72	189	0.57	205
UMGS775	159	0.82	66.71	77	0.022	0.78	162	0.82	137
Parabacteroides	160	0.82	67.03	76	0.029	0.76	172	0.61	195
Pelethocola	161	0.82	67.36	76	0.019	0.74	184	0.69	167
CAG.170	162	0.82	67.69	77	0.018	0.79	161	0.8	140
ER4	163	0.82	68.02	77	0.018	0.76	175	0.88	125
Mesosutterella	164	0.8	68.34	76	0.022	0.83	149	0.61	191
Clostridium_P	165	0.8	68.66	75	0.016	0.89	128	0.84	134
Catenibacterium	166	0.8	68.98	75	0.027	0.75	179	0.48	220
Sutterella	167	0.8	69.31	75	0.022	0.77	167	0.63	185
Pseudobutyricicoccus	168	0.78	69.62	73	0.029	0.75	180	0.59	200
UBA7173	169	0.78	69.93	73	0.029	0.83	150	0.56	209
Hungatella	170	0.78	70.25	73	0.018	0.76	174	0.77	152
UMGS1375	171	0.78	70.56	73	0.016	0.83	146	0.85	132
Marseille.P3106	172	0.78	70.88	73	0.02	0.8	156	0.83	135
CAG.115	173	0.78	71.19	72	0.027	0.62	224	0.61	194
Pelethousia	174	0.78	71.5	72	0.023	0.81	153	0.79	144
CAG.353	175	0.78	71.81	72	0.023	0.69	201	0.64	180
RUG115	176	0.77	72.12	73	0.02	0.75	178	0.76	154
UBA6398	177	0.77	72.43	72	0.033	0.64	217	0.56	210
CAG.245	178	0.77	72.74	73	0.02	0.82	151	0.79	143
HGM10836	179	0.77	73.05	72	0.027	0.68	205	0.61	190
Pseudoscilispira	180	0.77	73.36	72	0.03	0.71	195	0.57	204
Clostridium_A	181	0.77	73.67	71	0.029	0.68	204	0.63	186
Enterenecus	182	0.76	73.98	71	0.02	0.78	163	0.63	182
Desulfovibrio	183	0.76	74.28	72	0.023	0.73	187	0.45	232
Enterobacter	184	0.76	74.59	71	0.018	0.75	181	0.69	166
Clostridium_AP	185	0.76	74.89	71	0.027	0.76	170	0.66	172
Phascolarctobacterium	186	0.76	75.2	70	0.023	0.69	202	0.65	176
Faecalibacillus	187	0.76	75.5	71	0.027	0.76	173	0.64	181
Collinsella	188	0.76	75.8	71	0.02	0.69	200	0.59	198
Ruthenibacterium	189	0.76	76.11	70	0.021	0.72	188	0.59	199
Haemophilus_D	190	0.75	76.41	70	0.021	0.75	183	0.63	184
Merdimorpha	191	0.75	76.71	70	0.033	0.7	199	0.49	218
Coprococcus	192	0.75	77.01	70	0.019	0.79	159	0.63	183
Brachyspira	193	0.75	77.31	71	0.024	0.76	177	0.61	193
UMGS1370	194	0.75	77.61	70	0.031	0.66	211	0.65	177
Fimisoma	195	0.75	77.91	69	0.018	0.71	191	0.58	202
Anaerostipes	196	0.74	78.21	69	0.018	0.62	222	0.79	142
Eisenbergiella	197	0.74	78.51	69	0.027	0.65	216	0.58	203
Metalachnospira	198	0.74	78.81	69	0.02	0.77	168	0.65	178
Onthomonas	199	0.73	79.1	69	0.027	0.66	210	0.59	201
Scatocola	200	0.73	79.4	69	0.029	0.64	218	0.56	208

Table S11: Genera ranked 151–200 by Fractional Influence Score (FIS) under the default 90% posterior selection criterion in the CRC cohort. The table reports genus-specific FIS rankings, cumulative FIS contribution, number of selected metabolites connected to each genus, median Bayesian posterior edge significance, and corresponding FIS/rank summaries obtained under stricter 95% and 99% posterior selection criteria.

Genus	FIS rank (default; 90% CI based)	FIS (default; 90% CI based)	Cumulative FIS (%)	Num. Selected metabolites	Median Bayesian p-value (default; 90% CI-based)	FIS (95% CI based)	Rank (95% CI based)	FIS (99% CI based)	Rank (99% CI based)
Sodaliphilus	201	0.73	79.69	69	0.031	0.62	220	0.61	192
Peptostreptococcus	202	0.73	79.99	68	0.026	0.71	194	0.6	197
Veillonella_A	203	0.73	80.28	68	0.016	0.67	206	0.75	158
Rikenella	204	0.73	80.57	68	0.017	0.79	157	0.77	150
Fusobacterium_A	205	0.73	80.86	68	0.018	0.7	197	0.61	196
CAZU01	206	0.73	81.15	67	0.024	0.73	186	0.5	217
Fusicatenibacter	207	0.72	81.44	68	0.028	0.68	203	0.48	219
CAG.485	208	0.72	81.73	67	0.02	0.66	209	0.47	225
Niameybacter	209	0.72	82.02	67	0.016	0.71	192	0.65	174
Adlercreutzia	210	0.72	82.31	67	0.02	0.76	169	0.76	157
UBA5026	211	0.72	82.6	67	0.024	0.7	198	0.51	215
Senegalimassilia	212	0.71	82.89	67	0.02	0.71	190	0.65	175
Frisingicoccus	213	0.71	83.17	66	0.031	0.67	207	0.46	228
Akkermansia	214	0.7	83.45	66	0.011	0.75	182	0.86	130
Duodenibacillus	215	0.7	83.74	66	0.027	0.65	213	0.52	214
AM51.8	216	0.7	84.02	65	0.022	0.66	212	0.67	168
Eubacterium_G	217	0.69	84.3	65	0.022	0.7	196	0.64	179
CAG.127	218	0.69	84.57	65	0.038	0.58	232	0.41	242
Roseburia	219	0.69	84.85	65	0.031	0.65	214	0.42	238
Enterococcus_B	220	0.69	85.12	64	0.024	0.71	193	0.44	234
CAG.217	221	0.69	85.4	64	0.034	0.6	227	0.57	207
Aeromonas	222	0.69	85.68	64	0.032	0.65	215	0.48	223
Merdimonas	223	0.68	85.95	64	0.024	0.61	226	0.62	188
Citrobacter_B	224	0.68	86.22	64	0.031	0.63	219	0.46	229
Paraprevotella	225	0.67	86.49	63	0.031	0.53	246	0.48	221
Ruminococcus_B	226	0.67	86.76	63	0.033	0.55	240	0.51	216
Ventrisoma	227	0.67	87.03	62	0.033	0.54	244	0.47	224
Mediterraneibacter	228	0.66	87.3	62	0.024	0.67	208	0.57	206
CAG.873	229	0.66	87.56	62	0.021	0.58	231	0.62	187
Scatavimonas	230	0.66	87.83	62	0.031	0.59	229	0.41	241
Ruminococcus	231	0.66	88.1	62	0.038	0.6	228	0.41	240
CAG.273	232	0.65	88.36	62	0.031	0.56	238	0.44	236
Spyradocola	233	0.65	88.62	61	0.042	0.54	245	0.3	261
Fimivivens	234	0.65	88.88	61	0.033	0.57	236	0.38	245
CAG.349	235	0.65	89.14	61	0.022	0.62	223	0.77	153
UMGS1975	236	0.64	89.4	60	0.032	0.58	230	0.43	237
Butyricimonas	237	0.64	89.66	60	0.033	0.55	241	0.44	233
Dysosmobacter	238	0.64	89.92	60	0.033	0.56	237	0.32	256
Pullichristensenella	239	0.63	90.17	59	0.04	0.49	256	0.46	231
Alcanivorax	240	0.63	90.42	59	0.038	0.49	254	0.3	259
Marvinbryantia	241	0.63	90.67	58	0.027	0.61	225	0.46	230
Faecimonas	242	0.62	90.93	58	0.031	0.62	221	0.34	255
HGM13006	243	0.62	91.18	58	0.037	0.54	242	0.38	246
UMGS946	244	0.61	91.42	58	0.024	0.57	233	0.55	211
COE1	245	0.61	91.67	57	0.027	0.55	239	0.47	227
CAG.590	246	0.6	91.91	56	0.036	0.54	243	0.25	270
Dorea_A	247	0.6	92.15	56	0.034	0.51	251	0.28	264
Erysipelatoclostridium	248	0.6	92.39	56	0.036	0.45	264	0.38	247
Caecibacter	249	0.58	92.62	55	0.04	0.44	265	0.38	244
CAG.103	250	0.58	92.86	54	0.037	0.51	249	0.53	213

Table S12: Genera ranked 201–250 by Fractional Influence Score (FIS) under the default 90% posterior selection criterion in the CRC cohort. The table reports genus-specific FIS rankings, cumulative FIS contribution, number of selected metabolites connected to each genus, median Bayesian posterior edge significance, and corresponding FIS/rank summaries obtained under stricter 95% and 99% posterior selection criteria.

Genus	FIS rank (default; 90% CI based)	FIS (default; 90% CI based)	Cumulative FIS (%)	Num. Selected metabolites	Median Bayesian p-value (default; 90% CI-based)	FIS (95% CI based)	Rank (95% CI based)	FIS (99% CI based)	Rank (99% CI based)
Ruminococcus_E	251	0.58	93.09	54	0.029	0.57	235	0.35	253
CAG.238	252	0.58	93.32	53	0.036	0.48	258	0.36	250
Holdemanella	253	0.57	93.55	54	0.024	0.51	252	0.37	248
Allisonella	254	0.57	93.78	54	0.039	0.44	266	0.24	273
Citrobacter	255	0.57	94.01	53	0.038	0.48	260	0.35	251
Bifidobacterium	256	0.57	94.24	53	0.033	0.53	247	0.42	239
Klebsiella	257	0.56	94.46	53	0.042	0.43	269	0.2	276
Acidaminococcus	258	0.56	94.69	53	0.018	0.57	234	0.44	235
SZUA.378	259	0.56	94.91	52	0.032	0.51	250	0.39	243
SFEL01	260	0.55	95.13	51	0.036	0.45	263	0.2	275
UBA11774	261	0.54	95.35	51	0.049	0.36	276	0.31	258
CAG.302	262	0.54	95.57	50	0.038	0.42	270	0.26	266
Blautia	263	0.54	95.78	50	0.036	0.45	262	0.21	274
Azonexus	264	0.53	95.99	49	0.033	0.52	248	0.29	262
Oribacterium	265	0.53	96.21	50	0.034	0.48	257	0.26	268
UBA1394	266	0.52	96.42	49	0.024	0.5	253	0.29	263
UBA1685	267	0.51	96.62	48	0.03	0.4	274	0.35	252
Scatacola_A	268	0.51	96.83	48	0.027	0.49	255	0.25	272
Parasutterella	269	0.51	97.03	48	0.033	0.43	267	0.34	254
Massilistercora	270	0.5	97.23	47	0.036	0.47	261	0.17	278
Ruminococcus_C	271	0.5	97.44	47	0.04	0.38	275	0.16	279
Phocaeicola	272	0.5	97.64	46	0.034	0.48	259	0.25	271
RUG705	273	0.49	97.83	46	0.059	0.29	281	0.26	267
KLE1615	274	0.47	98.02	44	0.027	0.42	271	0.32	257
Chitinophaga	275	0.46	98.21	43	0.031	0.43	268	0.3	260
Ruminiclostridium_E	276	0.45	98.39	42	0.034	0.4	272	0.28	265
Limivicinus	277	0.43	98.56	40	0.039	0.35	277	0.11	286
Pseudoflavonifractor	278	0.42	98.73	39	0.051	0.27	283	0.26	269
GCA.900066755	279	0.41	98.89	38	0.032	0.33	279	0.16	281
Escherichia	280	0.41	99.06	38	0.032	0.4	273	0.37	249
Anaeroglobus	281	0.4	99.22	37	0.04	0.34	278	0.11	285
Ventricola	282	0.4	99.38	37	0.038	0.33	280	0.2	277
Fournierella	283	0.4	99.54	37	0.053	0.23	285	0.16	282
Butyrivibrio_A	284	0.35	99.68	32	0.034	0.28	282	0.16	280
Coprococcus_A	285	0.34	99.81	31	0.04	0.27	284	0.13	283
Kluyvera	286	0.27	99.92	25	0.055	0.17	286	0.1	287
Alloprevotella	287	0.2	100	19	0.033	0.17	287	0.12	284

Table S13: Genera ranked 251–287 by Fractional Influence Score (FIS) under the default 90% posterior selection criterion in the CRC cohort. The table reports genus-specific FIS rankings, cumulative FIS contribution, number of selected metabolites connected to each genus, median Bayesian posterior edge significance, and corresponding FIS/rank summaries obtained under stricter 95% and 99% posterior selection criteria.

S5.3 Case Study Inference

The B-MASTER analysis provides detailed insights into the associations between microbial genera and metabolites in two biologically relevant subsets. For clarity, we describe each finding with explicit direction and Bayesian p-values, as summarized in Tables S14 and S15. Positive associations are shown in red, negative associations in blue, and non-significant effects ($p > 0.05$) are denoted by "–".

For the abundant metabolite subset (Subset 1), several genera show broad regulatory influence. *Veillonella* demonstrates consistent and statistically significant associations across multiple metabolites. Specifically, it is negatively associated with propionate ($p = 0.033$, negative), butyrate ($p = 0.016$, negative), glutamate ($p < 0.001$, negative), 5-aminopentanoate ($p = 0.004$, negative), lysine ($p < 0.001$, negative), and alanine ($p = 0.036$, negative). In contrast, it exhibits positive associations with dihydrouracil ($p = 0.002$, positive) and urea ($p = 0.013$, positive). These results indicate a mixed regulatory profile in which *Veillonella* simultaneously promotes nitrogen metabolism while suppressing several amino acid pathways.

	Propionate	Butyrate	Dihydrouracil	Glutamate	Urea	Succinate	5-Aminopentanoate	Valerate	Lysine	Alanine
Veillonella	0.033	0.016	0.002	< 0.001	0.013	–	0.004	–	< 0.001	0.036
Oliverpabstia	–	0.031	0.011	< 0.001	< 0.001	–	< 0.001	–	0.004	0.002
Merdicola	–	< 0.001	0.042	< 0.001	0.038	0.022	–	< 0.001	0.011	–
Sellimonas	< 0.001	0.013	0.002	–	–	–	0.013	< 0.001	–	0.002
Avimicrobium	–	–	0.002	< 0.001	–	0.024	0.002	–	< 0.001	0.007
UBA4372	0.020	< 0.001	–	0.002	0.009	0.011	–	< 0.001	–	–
Enterocloster	0.002	–	< 0.001	–	0.022	0.011	–	–	0.018	0.007
Mogibacterium	0.007	–	–	< 0.001	< 0.001	0.047	–	< 0.001	–	0.011
Lancefieldella	–	–	0.018	< 0.001	–	–	< 0.001	0.049	< 0.001	0.002
Turicibacter	0.024	–	< 0.001	–	–	0.038	–	0.018	< 0.001	< 0.001
Intestinibacter	0.031	0.004	0.002	0.002	0.033	–	–	–	0.011	–
Limiplasma	0.002	0.002	0.040	–	0.024	0.007	–	–	–	0.036
Anaerotignum	–	–	–	0.047	0.016	0.049	0.004	–	0.002	< 0.001
Pauljensenia	–	0.049	0.024	0.036	–	–	0.002	< 0.001	–	0.009
An92	0.002	0.042	–	–	0.002	0.042	–	–	0.027	0.009

Table S14: Bayesian p-values for associations between genera (rows) and metabolites (columns) in subset 1 (most abundant metabolites). Values in red indicate positive associations, values in blue indicate negative associations, and "–" denotes $p > 0.05$ or missing. Values equal to 0 are displayed as "< 0.001".

Oliverpabstia is negatively associated with butyrate ($p = 0.031$, negative), glutamate ($p < 0.001$, negative), and lysine ($p = 0.004$, negative), while positively associated with dihydrouracil ($p = 0.011$, positive), urea ($p < 0.001$, positive), and 5-aminopentanoate ($p < 0.001$, positive). These results highlight both SCFA and amino acid regulation. *Merdicola* shows negative associations with butyrate ($p < 0.001$, negative), dihydrouracil ($p = 0.042$,

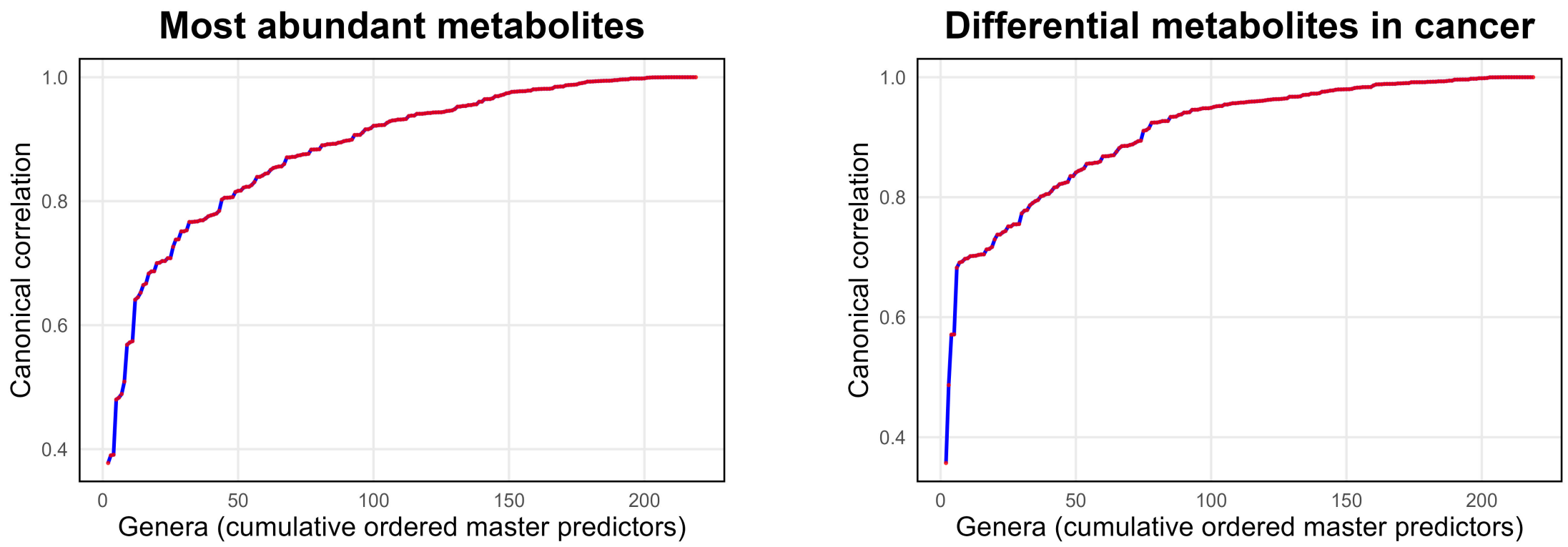

Figure S11: The canonical correlation between metabolites in Subset 1 (left) and Subset 2 (right) and the cumulatively included master predictors, arranged by their rank order, is visually depicted. The ranking of the predictors based on their prevalence is derived separately for each corresponding subset of metabolites.

negative), glutamate ($p < 0.001$, negative), and valerate ($p < 0.001$, negative), while exhibiting positive links with urea ($p = 0.038$, positive), succinate ($p = 0.022$, positive), and lysine ($p = 0.011$, positive). Taken together, *Merdicola* appears to shift metabolism toward nitrogenous compounds at the expense of SCFAs.

Sellimonas is exclusively positively associated: propionate ($p < 0.001$, positive), butyrate ($p = 0.013$, positive), dihydrouracil ($p = 0.002$, positive), 5-aminopentanoate ($p = 0.013$, positive), and valerate ($p < 0.001$, positive), although it is negatively associated with alanine ($p = 0.002$, negative). *Avimicrobium* demonstrates positive effects on dihydrouracil ($p = 0.002$, positive), glutamate ($p < 0.001$, positive), 5-aminopentanoate ($p = 0.002$, positive), lysine ($p < 0.001$, positive), and alanine ($p = 0.007$, positive), while exerting a negative effect on succinate ($p = 0.024$, negative).

UBA4372 exhibits negative associations with propionate ($p = 0.020$, negative), butyrate ($p < 0.001$, negative), glutamate ($p = 0.002$, negative), and valerate ($p < 0.001$, negative), while positively associated with urea ($p = 0.009$, positive) and succinate ($p = 0.011$, positive). Similarly, *Enterocloster* shows positive associations with propionate ($p = 0.002$, positive) and alanine ($p = 0.007$, positive), but negative associations with dihydrouracil ($p < 0.001$, negative), urea ($p = 0.022$, negative), succinate ($p = 0.011$, negative), and lysine ($p = 0.018$, negative). *Mogibacterium* is positively associated with propionate ($p = 0.007$, positive), glutamate ($p < 0.001$, positive), and valerate ($p < 0.001$, positive), while negatively associated with urea ($p < 0.001$, negative), succinate ($p = 0.047$, negative), and alanine ($p = 0.011$, negative).

Lancefieldella contributes strong positive associations with glutamate ($p < 0.001$, positive), 5-aminopentanoate ($p < 0.001$, positive), valerate ($p = 0.049$, positive), lysine ($p < 0.001$, positive), and alanine ($p = 0.002$, positive), along with a negative association with dihydrouracil ($p = 0.018$, negative). *Turicibacter* exhibits predominantly negative associations: propionate ($p = 0.024$, negative), dihydrouracil ($p < 0.001$, negative), succinate ($p = 0.038$, negative), valerate ($p = 0.018$, negative), and alanine ($p < 0.001$, negative), but a positive association with lysine ($p < 0.001$, positive). *Intestinibacter* shows negative associations with propionate ($p = 0.031$, negative), butyrate ($p = 0.004$, negative), glutamate ($p = 0.002$, negative), and lysine ($p = 0.011$, negative), while positively associated with dihydrouracil ($p = 0.002$, positive) and urea ($p = 0.033$, positive). *Limiplasma* is positively associated with propionate ($p = 0.002$, positive), butyrate ($p = 0.002$, positive), dihydrouracil ($p = 0.040$, positive), and alanine ($p = 0.036$, positive), while negatively associated with urea ($p = 0.024$, negative) and succinate ($p = 0.007$, negative).

Anaerotignum shows exclusively negative associations: glutamate ($p = 0.047$, negative), urea ($p = 0.016$, negative), succinate ($p = 0.049$, negative), 5-aminopentanoate ($p = 0.004$, negative), lysine ($p = 0.002$, negative), and alanine ($p < 0.001$, negative). *Pauljensenia* demonstrates mixed effects, including negative associations with butyrate ($p = 0.049$, negative), glutamate ($p = 0.036$, negative), 5-aminopentanoate ($p = 0.002$, negative), valerate ($p < 0.001$, negative), and alanine ($p = 0.009$, negative), but a positive association with dihydrouracil ($p = 0.024$, positive). Finally, *An92* is positively associated with propionate ($p = 0.002$, positive), butyrate ($p = 0.042$, positive), urea ($p = 0.002$, positive), succinate ($p = 0.042$, positive), and lysine ($p = 0.027$, positive), while negatively associated with alanine ($p = 0.009$, negative).

For the differentially abundant metabolite subset (Subset 2), the pattern is more systemic, with many genera influencing nearly all metabolites. *Gemmiger* exhibits negative association with X_{DCA} ($p < 0.001$, negative) but positive associations with glycocholate ($p < 0.001$, positive), taurocholate ($p < 0.001$, positive), L-isoleucine ($p < 0.001$, positive), L-leucine ($p < 0.001$, positive), L-valine ($p < 0.001$, positive), L-phenylalanine ($p < 0.001$, positive), L-tyrosine ($p < 0.001$, positive), L-serine ($p < 0.001$, positive), and glycine ($p < 0.001$, positive). *Ligilactobacillus* shows uniformly positive associations, including glycocholate ($p = 0.002$, positive), taurocholate ($p = 0.022$, positive), isovalerate ($p = 0.011$, positive), L-isoleucine ($p < 0.001$, positive), L-leucine ($p < 0.001$, positive),

	X_DCA	Glycocholate	Taurocholate	Isovalerate	L-Isoleucine	L-Leucine	L-Valine	L-Phenylalanine	L-Tyrosine	L-Serine	Glycine
Gemmiger	< 0.001	< 0.001	< 0.001	–	< 0.001	< 0.001	< 0.001	< 0.001	< 0.001	< 0.001	< 0.001
Ligilactobacillus	–	0.002	0.022	0.011	< 0.001	< 0.001	< 0.001	< 0.001	< 0.001	< 0.001	< 0.001
Faecalimonas	–	0.009	0.009	< 0.001	< 0.001	< 0.001	< 0.001	0.002	0.024	0.009	< 0.001
Bacteroides	–	0.040	0.027	–	< 0.001	< 0.001	< 0.001	< 0.001	< 0.001	0.007	0.002
Agathobacter	0.013	< 0.001	–	0.031	< 0.001	< 0.001	< 0.001	< 0.001	0.002	–	0.036
Caccousia	–	–	0.002	0.002	< 0.001	< 0.001	< 0.001	–	0.002	< 0.001	< 0.001
CAG.83	0.002	–	–	0.009	0.024	< 0.001	0.002	< 0.001	< 0.001	–	0.002
Anaerotignum	–	–	0.002	< 0.001	< 0.001	0.004	< 0.001	0.020	–	< 0.001	0.049
Flavonifractor	–	< 0.001	0.004	–	0.036	0.031	0.009	< 0.001	< 0.001	0.020	–
Amedibacterium	–	0.020	–	0.020	< 0.001	< 0.001	< 0.001	0.004	–	0.044	0.016
1XD42.69	–	–	0.013	–	< 0.001	0.002	0.002	< 0.001	0.002	< 0.001	–
Evtepia	–	–	–	< 0.001	0.009	0.011	0.007	< 0.001	< 0.001	0.004	–
Choladocola	–	0.016	–	< 0.001	0.004	0.002	< 0.001	< 0.001	0.011	–	–
Lachnospira	–	0.016	–	–	< 0.001	< 0.001	< 0.001	0.002	< 0.001	0.042	–
Merdicola	–	< 0.001	0.007	–	0.002	< 0.001	0.007	< 0.001	0.047	–	–

Table S15: Bayesian p-values for associations between top 15 genera (rows) and metabolites (columns) in subset 2 (differentially abundant metabolites in cancer vs control). Values in red indicate positive associations, values in blue indicate negative associations, and "–" denotes $p > 0.05$. Values < 0.001 are displayed as "< 0.001".

and all other amino acids ($p < 0.001$, positive).

Faecalimonas is positively associated with glycocholate ($p = 0.009$, positive), taurocholate ($p = 0.009$, positive), isovalerate ($p < 0.001$, positive), and all seven amino acids ($p \leq 0.024$, positive). In contrast, *Bacteroides* exhibits exclusively negative associations, including glycocholate ($p = 0.040$, negative), taurocholate ($p = 0.027$, negative), and all seven amino acids ($p \leq 0.007$, negative). *Agathobacter* is uniformly positive, including X_{DCA} ($p = 0.013$, positive), glycocholate ($p < 0.001$, positive), isovalerate ($p = 0.031$, positive), and multiple amino acids ($p \leq 0.036$, positive). Similarly, *Caccousia* exhibits positive associations with taurocholate ($p = 0.002$, positive), isovalerate ($p = 0.002$, positive), L-isoleucine ($p < 0.001$, positive), and all subsequent amino acids ($p < 0.001$, positive).

CAG.83 demonstrates consistent negative associations, including X_DCA ($p = 0.002$, negative), isovalerate ($p = 0.009$, negative), L-isoleucine ($p = 0.024$, negative), L-leucine ($p < 0.001$, negative), L-valine ($p = 0.002$, negative), L-phenylalanine ($p < 0.001$, negative), L-tyrosine ($p < 0.001$, negative), and glycine ($p = 0.002$, negative). *Anaerotignum* similarly shows broad negative associations, including taurocholate ($p = 0.002$, negative), isovalerate ($p < 0.001$, negative), L-isoleucine ($p < 0.001$, negative), L-leucine ($p = 0.004$, negative), L-valine ($p < 0.001$, negative), L-phenylalanine ($p = 0.020$, negative), L-serine ($p < 0.001$, negative), and glycine ($p = 0.049$, negative).

Flavonifractor shows mixed directionality, with positive associations for glycocholate ($p < 0.001$, positive), taurocholate ($p = 0.004$, positive), and L-serine ($p = 0.020$, positive), but negative associations for L-isoleucine ($p = 0.036$, negative), L-leucine ($p = 0.031$, negative), L-valine ($p = 0.009$, negative), L-phenylalanine ($p < 0.001$, negative), and L-

tyrosine ($p < 0.001$, negative). Other genera with uniform negative associations include *Amedibacterium* (glycocholate $p = 0.020$, negative; isovalerate $p = 0.020$, negative; and several amino acids $p \leq 0.044$, negative) and *1XD42.69* (taurocholate $p = 0.013$, negative; multiple amino acids $p \leq 0.002$, negative). *Evtepia* is strongly positive across metabolites, including isovalerate ($p < 0.001$, positive), L-isoleucine ($p = 0.009$, positive), L-leucine ($p = 0.011$, positive), L-valine ($p = 0.007$, positive), L-phenylalanine ($p < 0.001$, positive), L-tyrosine ($p < 0.001$, positive), and L-serine ($p = 0.004$, positive).

Choladocola exhibits a predominantly negative profile, including glycocholate ($p = 0.016$, negative), L-isoleucine ($p = 0.004$, negative), L-leucine ($p = 0.002$, negative), L-valine ($p < 0.001$, negative), L-phenylalanine ($p < 0.001$, negative), and L-tyrosine ($p = 0.011$, negative), with only one positive association observed for isovalerate ($p < 0.001$, positive). *Lachnospira* also shows a mixed pattern, with a single positive association to glycocholate ($p = 0.016$, positive) but negative associations with several amino acids, including L-isoleucine ($p < 0.001$, negative), L-leucine ($p < 0.001$, negative), L-valine ($p < 0.001$, negative), L-phenylalanine ($p = 0.002$, negative), L-tyrosine ($p < 0.001$, negative), and L-serine ($p = 0.042$, negative). Finally, *Merdicola* demonstrates exclusively positive associations, including glycocholate ($p < 0.001$, positive), taurocholate ($p = 0.007$, positive), L-isoleucine ($p = 0.002$, positive), L-leucine ($p < 0.001$, positive), L-valine ($p = 0.007$, positive), L-phenylalanine ($p < 0.001$, positive), and L-tyrosine ($p = 0.047$, positive).

In summary, Subset 1 results reveal genera with targeted effects on SCFA and amino acid pathways, while Subset 2 uncovers systemic positive or negative regulators of CRC-linked metabolites. Explicit reporting of Bayesian p-values and directionality highlights the statistical robustness of these associations and provides a foundation for downstream biological validation.

S5.4 Disease-stratified comparison of master predictors

To examine whether analogous master predictors could also be identified among healthy subjects, we performed an additional B-MASTER analysis using the healthy control samples ($n = 127$) while retaining the original CRC-based analysis presented in the main manuscript unchanged. The same microbiome-metabolite modeling framework, posterior selection rule, and Fractional Influence Score (FIS) definition were applied to the healthy cohort. This enabled a direct comparison of genus-level master predictor rankings and

influence structures between CRC and healthy subjects, allowing us to assess whether the inferred microbiome-metabolite influence architecture was shared or disease-specific.

Figure S12 summarizes this comparison. The scatterplot of CRC versus healthy FIS values in Figure S12(a) shows substantial separation between CRC-specific and healthy-specific top-ranked genera. Only three genera were shared between the CRC and healthy top-50 FIS rankings, whereas 47 genera were CRC-specific top-50 genera and 47 were healthy-specific top-50 genera. This corresponds to a Jaccard overlap of only 0.0309, indicating that the highest-ranking master predictors were largely distinct across the two cohorts.

The rank comparison in Figure S12(b) provides a complementary view of this disease-specific reordering. Many CRC-specific top-50 genera had substantially lower ranks in the healthy cohort, and conversely many healthy-specific top-50 genera were ranked much lower in CRC. This pattern supports the interpretation that B-MASTER is not merely identifying genera with uniformly high influence across all subjects, but rather detecting cohort-specific microbiome-metabolite regulatory patterns.

The cumulative FIS curves in Figure S12(c) further suggest that the distribution of influence differs between groups. The top 50 genera accounted for 24.85% of total FIS in the CRC cohort, compared with 41.98% in the healthy cohort. Thus, the healthy cohort exhibited a more concentrated influence structure, whereas the CRC cohort showed a more diffuse distribution of microbiome-metabolite influence across genera. The corresponding top-50 classification counts are shown in Figure S12(d).

Table S16 lists the leading differentially enriched master predictors. Among CRC-enriched genera, *Choladocola*, *Caccousia*, *Mogibacterium*, *Streptococcus*, and *Bacteroides* showed large positive differential FIS values and pronounced upward rank shifts in CRC relative to healthy controls. Conversely, *Oribacterium*, *Faecalicoccus*, *Allisonella*, *Rikenella*, and *Bifidobacterium* showed substantially larger FIS values in healthy subjects. These patterns are also visualized in Figure S12(e)–(f), which display the largest CRC-enriched and healthy-enriched differential FIS values, respectively.

The target-set comparison was defined consistently with the master predictor analysis: for a given genus, its target set consists of the metabolites selected as associated with that genus under the posterior selection rule used for the FIS calculation. Under this definition, differentially enriched master predictors also exhibited marked differences in

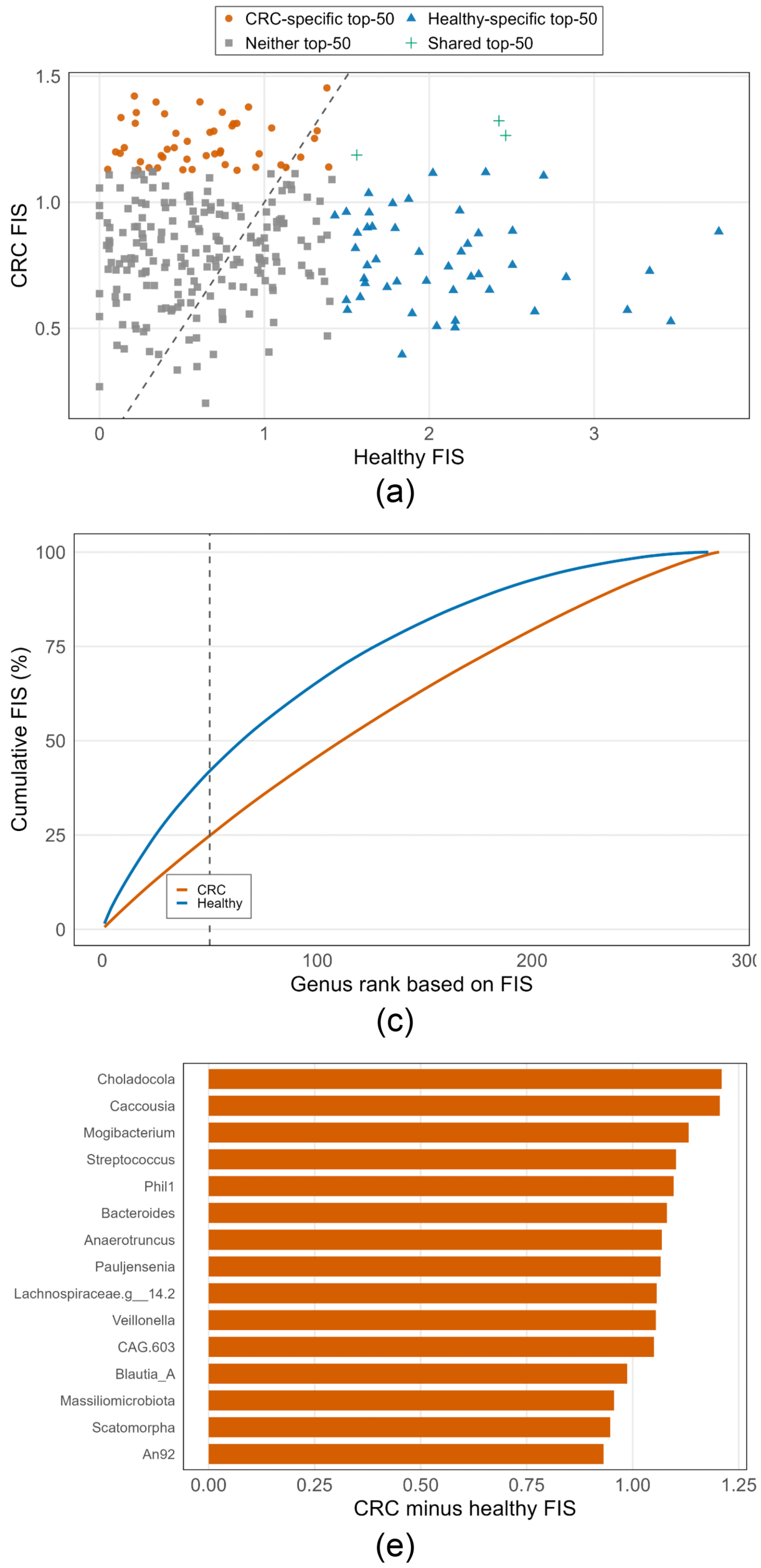

Figure S12: Comparison of master predictor structures identified by B-MASTER in colorectal cancer (CRC) and healthy cohorts. (a) Scatterplot comparing genus-level Fractional Influence Scores (FIS) between healthy and CRC cohorts, highlighting CRC-specific top-50 genera, healthy-specific top-50 genera, shared top-50 genera, and remaining genera. (b) Comparison of genus rankings based on FIS between healthy and CRC cohorts. The diagonal dashed line denotes identical rankings across cohorts. (c) Cumulative FIS concentration curves for CRC and healthy cohorts. The dashed vertical line marks the top-50 genera threshold. (d) Number of genera classified as CRC-specific top-50, healthy-specific top-50, or shared top-50 across cohorts. (e) Top genera exhibiting the largest positive differential influence scores (CRC minus healthy FIS), representing CRC-enriched master predictors. (f) Top genera exhibiting the largest negative differential influence scores (healthy minus CRC FIS), representing healthy-enriched master predictors.

Category	Genus	CRC rank	CRC FIS	Healthy rank	Healthy FIS	ΔFIS (CRC - Healthy)	Percentile shift
CRC-enriched (Top 10)	Choladocola	2	1.42	251	0.21	1.21	87.1
	Caccousia	9	1.34	262	0.13	1.21	88.5
	Mogibacterium	7	1.36	242	0.22	1.13	82.2
	Streptococcus	27	1.20	270	0.10	1.10	85.0
	Phil1	11	1.31	245	0.22	1.10	81.8
	Bacteroides	46	1.13	279	0.05	1.08	81.5
	Anaerotruncus	29	1.19	263	0.13	1.07	81.8
	Pauljensenia	23	1.22	258	0.15	1.07	82.2
	Lachnospiraceae.g_14.2	70	1.06	282	0.00	1.06	74.1
	Veillonella	4	1.40	214	0.34	1.05	73.4
Healthy-enriched (Top 10)	Oribacterium	265	0.53	2	3.47	-2.94	-92.0
	Faecalicoccus	131	0.88	1	3.76	-2.87	-45.5
	Allisonella	254	0.57	4	3.20	-2.63	-87.4
	Rikenella	204	0.73	3	3.34	-2.61	-70.3
	Duodenibacillus	215	0.70	5	2.83	-2.13	-73.4
	Bifidobacterium	256	0.57	7	2.64	-2.07	-87.1
	Merdimorpha	191	0.75	9	2.50	-1.75	-63.6
	Spyradocola	233	0.65	12	2.37	-1.71	-77.3
	Massilistercora	270	0.50	21	2.16	-1.65	-87.1
	Azonexus	264	0.53	20	2.16	-1.63	-85.3
Shared top-50	Succinivibrio	10	1.32	11	2.42	-1.10	0.3
	CAG.83	20	1.26	10	2.46	-1.20	-3.5
	Onthousia	32	1.19	45	1.56	-0.37	4.5

Table S16: Differential comparison of master predictors identified by B-MASTER in colorectal cancer (CRC) and healthy cohorts. The table summarizes the top CRC-enriched genera (largest positive ΔFIS), top healthy-enriched genera (largest negative ΔFIS), and representative genera shared among the top-50 FIS rankings in both cohorts. For each genus, we report the cohort-specific Fractional Influence Score (FIS), corresponding FIS rank, differential FIS ($\Delta\text{FIS} = \text{FIS}_{\text{CRC}} - \text{FIS}_{\text{Healthy}}$), and percentile rank shift between cohorts. Large positive percentile shifts indicate substantially stronger influence in CRC relative to healthy controls, whereas large negative shifts indicate stronger influence in healthy subjects.

target-set breadth. For example, several CRC-enriched genera had high CRC FIS values and broad CRC target sets while having little or no corresponding influence in healthy controls. This provides additional biological support for disease-associated restructuring of microbiome-metabolite relationships.

Overall, this disease-stratified analysis indicates that B-MASTER identifies both CRC-enriched and healthy-enriched master predictors, with limited overlap among the highest-ranking genera. These findings complement the main CRC-focused analysis and suggest that the inferred master predictor structure reflects disease-associated changes in system-level microbiome-metabolite regulation rather than a generic influence pattern shared uniformly across cohorts.

References

Banerjee, S. & Ghosal, S. (2014), 'Posterior convergence rates for estimating large precision matrices using graphical models', *Electronic Journal of Statistics* **8**(2), 2111–2137.

Castillo et al. (2015), 'Bayesian linear regression with sparse priors', *Annals of Statistics* **43**(5), 1986–2018.

Chicco, D. & Jurman, G. (2020), 'The advantages of the matthews correlation coefficient (mcc) over f1 score and accuracy in binary classification evaluation', *BMC Genomics* **21**(6).

Das, P., Peterson, C., Do, K. et al. (2020), 'Bayesian simultaneous network estimation across unequal sample sizes', *Bioinformatics* **36**(3), 798–804.

Deshpande et al. (2019), 'Simultaneous variable and covariance selection with the multivariate spike-and-slab lasso', *J. Comput. Graph.* **28**(4), 921–931.

Friedman, J., Hastie, T. & Tibshirani, R. (2008), 'Regularized paths for generalized linear models via coordinate descent', *Technical report, Dept. Statistics, Stanford Univ* .

Ghosal, S., Ghosh, J. & van der Vaart, A. (2000), 'Convergence rates of posterior distributions', *The Annals of Statistics* **28**(2), 500–531.

Kleijn, B. & van der Vaart, A. (2006), 'Misspecification in infinite-dimensional bayesian statistics', *Annals of Statistics* **34**(2), 837–877.

Kyung, M., Gill, J. & Ghosh, M. (2010), 'Penalized regression, standard errors, and bayesian lasso', *Bayesian Analysis* **5**(2), 369–411.

MathWorks (2024), 'Quick start parallel computing in MATLAB'. Accessed: 2024-11-19. **URL:** *https://www.mathworks.com/help/parallel-computing/*

Park, T. & Casella, G. (2008), 'The Bayesian lasso', *J. Am. Stat. Assoc.* **103**(482), 681–686.

Peng et al. (2009), 'Partial correlation estimation by joint sparse regression models', *J. Am. Stat. Assoc.* **104**(486), 735–746.

Peng et al. (2010), 'Regularized multivariate regression for identifying master predictors with application to integrative genomics study of breast cancer', *Ann. Appl. Stat.* **4**(1), 53–77.

Rockova, V. & George, E. (2018), 'The spike-and-slab lasso', *J. Am. Stat. Assoc.* **113**(521), 431–444.

Roy, V. & Chakraborty, S. (2017), 'Selection of tuning parameters, solution paths, and standard errors for Bayesian lasso', *Bayesian Analysis* **12**(3), 753–778.

Schwarz, G. (1978), 'Estimating the dimension of a model', *Ann. Stat.* **6**(2), 461–464.

Simpson et al. (2017), 'Penalising model component complexity: A principled, practical approach to constructing priors', *Statistical Science* **32**(1), 1–28.

Tibshirani, R. (1996), 'Regression shrinkage and selection via the Lasso', *J. R. Stat. Soc., B: Stat. Methodol.* **58**(1), 267–288.

van der Vaart, A. W. (1998), *Asymptotic Statistics*, Cambridge University Press.

Xu, X. & Ghosh, M. (2015), 'Bayesian variable selection and estimation for group lasso', *Bayesian Analysis* **10**(4), 909–936.

Yachida et al. (2019), 'Metagenomic and metabolomic analyses reveal distinct stage-specific phenotypes of the gut microbiota in colorectal cancer', *Nature Medicine* **25**(6), 968–976.

Zou, H. & Hastie, T. (2005), 'Regularization and variable selection via the elastic net', *J. R. Stat. Soc., B: Stat. Methodol.* **67**(2), 301–320.
